# Explainable Multi-Isoform QSAR, PubChem Concordance, and Applicability-Domain-Guided Prioritization of Selective Human Carbonic Anhydrase I, II, IX, and XII Inhibitors

**DOI:** 10.3390/ph19081322

**Published:** 2026-08-21

**Authors:** Alaa M. Elsayad, Khaled A. Elsayad

**Affiliations:** 1 Biomedical Group, Department of Electrical Engineering, College of Engineering, Prince Sattam Bin Abdulaziz University, Wadi Alddawasir 11991, Saudi Arabia; 2 Pharmacy Department, Cairo University Hospitals, Cairo University, Cairo 11662, Egypt; khaled.al.elsayad@std.pharma.cu.edu.eg

**Keywords:** carbonic anhydrase inhibitors, CA I, CA II, CA IX, CA XII, isoform selectivity, QSAR, H2O.ai AutoML, PubChem screening, experimental concordance, applicability domain, explainable artificial intelligence, SHAP, SwissADME, sulfonamide inhibitors

## Abstract

**Background/Objectives:** Human carbonic anhydrase (CA) isoforms are clinically relevant zinc metalloenzymes, but their highly conserved catalytic Zn^2+^ site makes isoform-selective inhibitor design challenging. This study developed an explainable multi-isoform QSAR workflow for prioritizing selective inhibitors of human CA I, CA II, CA IX, and CA XII, integrating PubChem experimental concordance and applicability-domain (AD) analysis to distinguish model-supported candidates from exploratory extrapolative hypotheses. **Methods:** Curated ChEMBL *K_i_* datasets were standardized to *pK_i_* and modeled in KNIME using 4185 two-dimensional descriptors, Random Forest-based feature selection, and H2O.ai AutoML stacked ensembles. A matched 3200-compound four-isoform matrix supported direct selectivity profiling. Potent ChEMBL inhibitors (*K_i_* < 10 nM) seeded 90% PubChem similarity expansion; analogues were scored across all four isoform-specific models, while PubChem records were filtered to retain only human isoform-specific *K_i_* evidence. AD was calibrated using Morgan fingerprint similarity, descriptor-space distance, and leverage analysis. Candidates were prioritized by predicted potency, selectivity, SAR plausibility, SwissADME profile, structural alerts, and AD membership. **Results:** Models achieved held-out R^2^ values of 0.727, 0.719, 0.652, and 0.607 for CA I, CA II, CA IX, and CA XII, respectively. PubChem concordance identified 1098 prioritized compounds with assay records, including 847 with direct *K_i_* evidence and 428 with complete four-isoform coverage; same-target agreement showed Pearson r > 0.86 and linear-fit R^2^ > 0.74. Test-set error rose from very-high-AD to low/out-of-domain classes. **Conclusions:** The workflow integrates QSAR prediction, PubChem concordance, explainable SAR, ADME triage, and AD-based reliability assessment. CA II and CA XII candidates showed the strongest support, CA IX showed intermediate support, and CA I candidates require cautious interpretation as low-domain exploratory hypotheses.

## 1. Introduction

Carbonic anhydrases (CAs, EC 4.2.1.1) are zinc metalloenzymes that catalyze the reversible hydration of carbon dioxide: CO_2_ + H_2_O ⇌ HCO_3_^−^ + H^+^. Although chemically simple, this reaction is fundamental to pH homeostasis, CO_2_ transport, bicarbonate buffering, ion exchange, fluid secretion, and several disease-associated microenvironmental processes. The CA superfamily spans prokaryotes and eukaryotes and comprises eight genetically distinct families: α, β, γ, δ, ζ, η, θ, and ι. Humans express only α-CAs, represented by 15 isoforms with distinct catalytic activity, tissue distribution, and subcellular localization. Among these, CA I and CA II are abundant cytosolic isoforms, whereas CA IX and CA XII are membrane-associated enzymes with particular relevance to tumor pH regulation, hypoxia adaptation, extracellular acidification, and cancer progression [1,2,3,4]. This biological diversity gives CAs therapeutic relevance across ophthalmology, neurology, nephrology, vascular biology, and oncology, but it also creates a central medicinal-chemistry challenge: potent inhibition must be coupled with isoform selectivity to avoid clinically undesirable off-target activity [4,5,6].

The selectivity problem is especially important for tumor-associated CA IX and CA XII. CA IX is strongly induced under hypoxia and contributes to extracellular acidification and intracellular alkalinization, thereby supporting tumor-cell survival, invasion, metastasis, and therapy resistance [2,3,7]. CA IX-targeted inhibitors, imaging agents, and therapeutic strategies have therefore attracted increasing attention in oncology [2,8,9]. CA XII is also frequently investigated together with CA IX because both isoforms contribute to tumor-associated pH regulation and represent attractive targets for selective inhibitor development [10,11,12]. However, classical CA inhibitors often contain primary sulfonamides or related zinc-binding groups that coordinate the conserved catalytic Zn^2+^ ion. This interaction explains their high potency but also their broad isoform inhibition, especially against the ubiquitous cytosolic isoform CA II [4,5,13]. Accordingly, next-generation CA inhibitor discovery requires not only nanomolar potency, but also quantitative selectivity profiling across CA I, CA II, CA IX, and CA XII [14,15].

At the structural level, isoform selectivity is governed less by the conserved catalytic Zn^2+^ core and more by subtle but exploitable differences in the active-site entrance and rim region. Variations around positions 68/70, 131, and 190–201 modulate pocket volume, hydrophobicity, steric accessibility, hydrogen-bonding capacity, and tail accommodation. These rim differences underpin major CA inhibitor design strategies, including the tail approach, dual-tail and three-tail approaches, entrance-channel plugging, coumarin/sulfocoumarin inhibition, heterocyclic scaffold optimization, and click-chemistry-based inhibitor diversification [4,5,6,11,13,16,17,18]. For example, the relatively narrow and aromatic rim of CA II can favor compact aromatic sulfonamide-bearing scaffolds, whereas the more permissive CA IX entrance may accommodate larger substituents that exploit tumor-associated rim differences. CA XII, by contrast, often tolerates flexible polar–hydrophobic tails because of its more open and solvent-exposed entrance region [1,4,5,16]. These structure–selectivity relationships provide a mechanistic rationale for descriptor-based QSAR modeling, fingerprint similarity analysis, and explainable machine-learning interpretation in the discovery of isoform-selective CA inhibitors [19,20].

Quantitative structure–activity relationship (QSAR) modeling has become a central component of ligand-based drug discovery because it enables activity prediction, chemical-space exploration, virtual screening, and lead prioritization before experimental testing [21,22,23]. Recent progress in molecular descriptors, fingerprints, automated machine learning, and explainable artificial intelligence has expanded the applicability of QSAR workflows to increasingly complex medicinal-chemistry problems, including CA inhibitor design [14,24,25,26,27,28,29]. Nevertheless, important limitations remain. Many CA QSAR studies focus on a single isoform, a narrow chemical series, or binary activity classification rather than continuous *K*_i_ or *pK_i_* prediction. Others provide useful activity prediction but limited multi-isoform selectivity assessment across both therapeutic targets and anti-targets [14,25,27,30]. Furthermore, high-performing machine-learning models such as gradient boosting, neural networks, and ensemble learners can remain mechanistically opaque unless complemented by interpretability methods such as permutation feature importance, SHAP analysis, descriptor interpretation, and applicability-domain diagnostics [31,32,33,34].

A further challenge is prospective generalization. Internal train/test splitting is necessary but insufficient for demonstrating virtual-screening utility, particularly when candidate compounds are close analogues of known inhibitors. Public bioactivity resources now enable a stronger workflow: curated ChEMBL data can be used to construct isoform-specific QSAR models, while PubChem similarity expansion can supply external analogue pools for experimental concordance testing and candidate discovery [35,36,37]. However, PubChem assay-summary data are heterogeneous and may include different CA isoforms, unrelated targets, IC_50_ values, cytotoxicity assays, selectivity-ratio rows, and non-*K_i_* endpoints. Therefore, PubChem-based concordance analysis must be filtered conservatively to distinguish true human isoform-specific *K_i_* evidence from unrelated assay records and from exact ChEMBL structure recovery.

A critical requirement for QSAR-guided virtual screening is not only prediction accuracy, but also an explicit definition of the chemical space within which predictions can be considered reliable. Applicability-domain analysis addresses this issue by identifying whether test compounds or newly prioritized candidates are sufficiently similar to the model’s training chemical space. This is particularly important for CA inhibitor discovery because classical sulfonamide zinc-binding inhibitors, coumarin-like entrance binders, sulfamates, heteroaryl scaffolds, and non-classical carboxylate-containing chemotypes may occupy different descriptor and fingerprint domains. Therefore, candidate ranking based only on predicted *pK_i_* or selectivity index may overstate confidence for structurally novel compounds. In the present work, AD analysis was used as an explicit reliability layer by combining Morgan fingerprint similarity, standardized descriptor-space five-nearest-neighbor distance, and leverage diagnostics. The held-out test subsets were used to calibrate the relationship between AD class and prediction error, and the resulting thresholds were then applied to final PubChem candidates.

In the present study, we developed an explainable, multi-isoform QSAR-guided workflow for the discovery and prioritization of selective CA inhibitors. Four isoform-specific QSAR regression models were built using curated ChEMBL *K_i_* datasets for human CA I, CA II, CA IX, and CA XII. In parallel, a matched 3200-compound four-isoform *K_i_* matrix was constructed to support compound-level selectivity profiling across target and off-target isoforms. Potent ChEMBL inhibitors were then used as seeds for 90% two-dimensional PubChem similarity expansion, and retrieved analogues were evaluated across all four QSAR models. Available PubChem assay records were conservatively filtered to retain only human isoform-specific *K_i_* evidence for external experimental concordance. Finally, candidate prioritization was refined through SAR interpretation, SwissADME profiling, structural-alert review, and applicability-domain analysis calibrated on held-out test subsets. Thus, the novelty of this work lies not only in building four CA isoform-specific QSAR models, but in integrating multi-isoform selectivity prediction, PubChem concordance, explainable descriptor interpretation, AD-based reliability stratification, and chemically informed candidate prioritization into a single reproducible workflow.

### Related Work

Over the past decade, QSAR modeling has evolved from classical linear regression and small-series modeling toward machine-learning-driven workflows supported by large curated bioactivity databases, high-dimensional molecular descriptors, chemical fingerprints, automated model selection, and rigorous validation practices. ChEMBL and PubChem have become foundational resources for bioactivity curation, analogue retrieval, and virtual screening, while descriptor platforms and cheminformatics toolkits support standardized molecular representation and similarity analysis [28,29,35,37]. In parallel, workflow environments such as KNIME and AutoML tools such as H2O.ai AutoML have facilitated reproducible construction, comparison, and deployment of machine-learning models in cheminformatics [38,39]. Recent QSAR platforms such as PoseidonQ further reflect this trend by integrating model development, validation, portability, and applicability-domain assessment into drug-discovery workflows [21].

In parallel, cheminformatics has shifted from purely predictive modeling toward interpretable and explainable machine learning. Explainable artificial intelligence methods, including SHAP, LIME, permutation feature importance, and related post hoc interpretation approaches, are increasingly used to attribute model predictions to molecular features and to translate statistical outputs into medicinal-chemistry hypotheses [31,32,33,34]. However, explanation quality depends on model architecture, descriptor correlation, data coverage, and applicability-domain structure. Therefore, interpretability should be paired with internal validation, external testing, stability assessment, and uncertainty-aware triage rather than treated as a substitute for rigorous model evaluation [32,40,41].

Within the carbonic anhydrase field, QSAR and machine-learning studies have progressed from activity modeling of restricted chemical series toward multi-isoform activity and selectivity prediction. Activity-focused models of pyrazoline, benzenesulfonamide, and related CA inhibitor chemotypes have shown that molecular descriptors can identify structural determinants of potency and support rational optimization [25,27,30]. More broadly, Tinivella et al. demonstrated the feasibility of predicting activity and selectivity profiles of human CA inhibitors across multiple isoforms using machine learning, highlighting the value of multi-output or multi-isoform thinking in CA inhibitor prioritization [14]. Fingerprint-based explainable modeling has also begun to identify substructural patterns associated with selective CA I/II inhibition, providing chemically interpretable cues for ligand optimization [24].

Recent CA IX-focused computational studies have also combined pharmacophore modeling, virtual screening, docking, and molecular simulation to prioritize selective tumor-associated CA IX inhibitors, further supporting the value of integrating ligand-based and structure-based prioritization strategies in this target class [17,42].

Despite this progress, several gaps remain. First, many published CA QSAR studies are limited to single isoforms or narrow scaffold families and do not explicitly rank compounds by selectivity across both tumor-associated targets and cytosolic anti-targets. Second, external validation is often restricted or ambiguous, particularly when analogue-based test sets overlap chemically with the training domain. Third, relatively few workflows combine isoform-specific *K_i_* prediction, PubChem-derived experimental concordance, applicability-domain analysis, and interpretable lead ranking in a single reproducible pipeline. The present work addresses these gaps by integrating ChEMBL-curated multi-isoform QSAR modeling, PubChem evidence filtering, fingerprint-based applicability-domain assessment, selectivity-index ranking, and prioritization for future zinc-aware docking for CA I, CA II, CA IX, and CA XII.

## 2. Results

### 2.1. Data Acquisition, Integration, and Multi-Isoform Analysis Framework

Four curated human carbonic anhydrase datasets were assembled from ChEMBL version 34 for CA I, CA II, CA IX, and CA XII. After endpoint standardization, the isoform-specific datasets contained 7204 CA I inhibitors, 7698 CA II inhibitors, 5631 CA IX inhibitors, and 4416 CA XII inhibitors, each associated with experimentally reported *K_i_* values in nM. These values were converted to *pK_i_* using:

$$pK_{i}=9-{log}_{10}(K_{i}\;[\mathrm{nM}])$$

The full isoform-specific datasets were retained for development of the four individual QSAR models, as they maximized the number of experimentally characterized compounds available for each target.

In parallel, a matched four-isoform activity matrix was constructed by retaining compounds with complete experimental profiles against all four modeled isoforms, yielding 3200 unique molecules with paired CA I, CA II, CA IX, and CA XII activity values. Unlike the larger isoform-specific datasets used for model development, this matched matrix served as a dedicated selectivity-analysis layer, enabling direct compound-level comparison of target and off-target potency within the same chemical structures. Target-specific selectivity indices were calculated as:

$$SI_{t}=\frac{min(K_{i}\;\mathrm{of}\;\mathrm{off-target}\;\mathrm{isoforms})}{K_{i}\;\mathrm{of}\;\mathrm{target}\;\mathrm{isoform}}$$

where *t* denotes the target isoform and the off-target isoforms are the remaining three modeled CAs; higher $SI_{t}$ values indicate stronger preference for the target isoform relative to its most sensitive off-target. Logarithmic selectivity differences were also calculated as:

$$\Delta pK_{i}(t)=pK_{i}(t)-max[pK_{i}(\mathrm{off-target}\;\mathrm{isoforms})]$$

with positive values indicating preferential inhibition of the target isoform.

The study therefore used three connected data layers. First, the full isoform-specific ChEMBL datasets supported CA I, CA II, CA IX, and CA XII QSAR model development. Second, the 3200-compound matched four-isoform matrix supported experimental selectivity profiling, SI and Δ*pK_i_* calculation, limiting off-target identification, and compound-level comparison across targets and anti-targets. Third, PubChem 90% similarity expansions from potent ChEMBL seeds generated external analogue pools for prospective virtual screening and post-ranking experimental concordance analysis.

The complete matched four-isoform matrix is provided as Supplementary Table S1, including ChEMBL identifiers, SMILES, four-isoform *K_i_* and *pK_i_* values, selectivity indices, Δ*pK_i_* endpoints, limiting off-target isoforms, and most-selective isoform assignments. The top-ranked experimentally selective molecules derived from this matrix are summarized in Supplementary Table S2, which provides the compound-level basis for the structural selectivity interpretation in Section 2.2 and Table 1.

The residues listed in Table 1 are not intended to represent the complete ligand-contact set observed in any single crystal structure. Instead, they summarize representative isoform-discriminating rim/entrance-region residues that are commonly used to rationalize CA isoform selectivity and tail-region accommodation. Direct ligand-contact residues may differ depending on the PDB structure, ligand chemotype, protonation state, and crystallographic water network. For example, catalytic and near-catalytic residues such as His94, Thr199, His200, Glu106, Leu198, and Trp209 may contact or stabilize ligands in individual structures, whereas Table 1 focuses on variable entrance/rim positions relevant to isoform selectivity rather than universal active-site contacts.

### 2.2. Structure-Guided Interpretation of the Experimental Selectivity Landscape

Before QSAR modeling, the curated matched four-isoform matrix (Supplementary Table S1) was examined as an experimental selectivity landscape in its own right. Because each molecule carries paired Ki values against CA I, CA II, CA IX, and CA XII, isoform preference could be compared directly at the compound level rather than inferred from disjoint single-target datasets. This paired design allowed the most selective compounds for each isoform to be ranked by target-specific selectivity index (SI) and interpreted in relation to established active-site rim differences at positions 68/70, 131, and 190/201. These rim-region differences modulate pocket size, steric accessibility, hydrophobicity, and tail accommodation, and therefore provide a structural basis for isoform-selective inhibition [4,13]. The resulting structural determinants and dominant design patterns are summarized in Table 1, while the top-20 selective molecules per isoform are provided in Supplementary Table S2.

Two complementary selectivity paradigms emerged from the matched matrix. The first was classical Zn^2+^-binding inhibition, in which a primary sulfonamide, sulfamide, sulfamate, or related zinc-binding group anchors the catalytic Zn^2+^ ion, whereas isoform preference is tuned by scaffold and tail substituents that interact with variable rim residues. This pattern dominated the CA II- and CA XII-selective subsets and is consistent with the established tail, dual-tail, and three-tail approaches for ligand/isoform matching [4,5,11].

The top-ranked CA II-selective ligand was CHEMBL4449193, a benzenesulfonamide oxime-amide with Ki values of 59.3, 0.05, 12,600, and 680 nM against CA I, CA II, CA IX, and CA XII, respectively. This compound reached a CA II SI of 1186.0, limited by CA I. Its profile illustrates the canonical CA II paradigm: strong Zn^2+^ anchoring by the sulfonamide group, combined with an aromatic oxime-amide tail compatible with the narrow CA II pocket and the Phe-131 rim contribution. The broader CA II top-20 set reinforces this interpretation, as most high-SI ligands were sulfonamides bearing compact aromatic, heteroaromatic, oxazole, thiophene, amide, urea, or oxime-amide tails.

CA XII showed an equally strong classical Zn^2+^-binding signature, but with a distinct tail-recognition profile. The top-ranked CA XII representative was CHEMBL3342494, a bis-sulfonamide cyanopyrrole bearing a bromophenyl substituent, with Ki values of 1870, 589, 849, and 0.68 nM against CA I, CA II, CA IX, and CA XII, respectively. This compound reached a CAXII SI of 866.2, limited by CA II. Its selectivity is consistent with compatibility between the appended scaffold and the more open CA XII rim. The Ala-131-permissive, solvent-exposed entrance can accommodate flexible heteroaryl or polar–hydrophobic tails, while Glu-190 may provide additional polar interaction opportunities. The CA XII top-20 set corroborates this interpretation, showing repeated enrichment of sulfonamide/sulfamate scaffolds bearing extended aryl, heteroaryl, sugar-like, urea, or flexible tail regions. This interpretation is also consistent with recent tail-approach studies of benzenesulfonamide CA inhibitors, in which thiadiazole, urea, and related tail modifications were used to tune potency and isoform recognition while retaining the classical sulfonamide zinc-binding pharmacophore [43].

The second paradigm was non-classical entrance- or rim-directed selectivity, governed more by steric fit, entrance topology, and rim complementarity than by direct primary sulfonamide-type Zn^2+^ coordination. This pattern was most prominent among the top CA I-selective compounds. The leading example, CHEMBL2070207, is an indole–pyrazole carboxylic acid with Ki values of 42.0, 1,820,000, 7790, and 7780 nM against CA I, CA II, CA IX, and CA XII, respectively. It reached a CA I SI of 185.2, limited by CA XII. Because this compound lacks a classical zinc-binding sulfonamide motif, it is better interpreted as a compact non-classical scaffold whose selectivity reflects entrance/rim complementarity within the narrower and more rigid CA I pocket. The next-ranked CA I-selective molecules, including CHEMBL568416 and CHEMBL568348, are coumarin-carboxylic acid isomers, further supporting the recurrence of compact carboxylate/coumarin-like chemotypes in the CA I-preferring subset. This interpretation is consistent with the known ability of coumarin-type CA inhibitors to exploit entrance-region interactions rather than canonical Zn^2+^ coordination [18].

CA IX displayed a more dualistic selectivity pattern, combining non-classical entrance-directed scaffolds with classical high-potency sulfonamides. The top CA IX representative was CHEMBL577123, a coumarin-carboxylic acid with Ki values of 6800, 9100, 48, and 8000 nM against CA I, CA II, CA IX, and CA XII, respectively. It reached a CAIX SI of 141.7, limited by CA I, consistent with a non-classical scaffold suited to the wider, more hydrophobic, and more permissive CA IX entrance. Alongside this compound, several highly potent CA IX-selective molecules in the top-20 set, including CHEMBL1836402, CHEMBL1836405, and CHEMBL1836407, were ethynyl aryl sulfonamides with low-nanomolar CA IX Ki values of 1.6, 1.1, and 1.0 nM, respectively. These molecules illustrate an alternative CA IX-selective route in which classical Zn^2+^ anchoring is retained, but hydrophobic/aromatic tail architecture improves compatibility with the CA IX rim while reducing CA I and CA II affinity.

Overall, the matched four-isoform matrix reproduced established CA structure–selectivity principles rather than behaving as an unstructured activity collection. CA II and CA XII selectivity aligned mainly with classical Zn^2+^-binding sulfonamide/sulfamate chemotypes tuned by tail–rim compatibility; CA I selectivity was enriched in compact non-classical carboxylate and coumarin-like scaffolds consistent with its narrow, rigid entrance; and CA IX selectivity was dualistic, spanning both non-classical coumarin-type inhibitors and classical hydrophobic-tailed sulfonamides. Thus, Supplementary Table S1 supplies the quantitative matched-activity foundation, Supplementary Table S2 documents the compound-level selectivity evidence, and Table 1 provides the structural framework against which subsequent QSAR descriptor importance, applicability-domain behavior, and virtual-screening priorities were interpreted.

### 2.3. Descriptor-Level Interpretation of Isoform-Specific Feature Selection

To characterize molecular determinants of carbonic anhydrase inhibition, compounds in the four isoform-specific ChEMBL datasets were encoded using 4185 two-dimensional alvaDesc descriptors. After leakage-controlled preprocessing, correlation filtering, and Random Forest-based feature ranking, the top-100 descriptors per isoform were retained for QSAR modeling and interpretation. The full top-100 descriptor panels are provided in Supplementary Table S3, while the top-ten descriptors for each isoform are summarized in Table 2. Detailed descriptor-generation, preprocessing, and feature-selection procedures are described in Section 4.4, Section 4.5 and Section 4.6.

The RF-ranked descriptors provided an initial data-driven view of isoform-specific ligand-recognition requirements. Across the four isoforms, the selected descriptors were not uniformly distributed, indicating that CA inhibition is not governed by a single universal molecular signature. Instead, activity against CA I, CA II, CA IX, and CA XII was associated with different combinations of molecular topology, electronic distribution, sulfur- and nitrogen-containing fragments, pharmacophore spacing, shape/electrostatic distribution, polar surface properties, and hydrogen-bonding potential. In this context, F03[O-S] denotes an oxygen–sulfur topological atom-pair descriptor at path length 3, not a fluorine-containing fragment.

For CA I, the top-ranked features were dominated by Burden/eigenvalue-type descriptors, including SpMax2_Bh(e), SpMax2_Bh(v), SpMax2_Bh(m), SpMax3_Bh(m), and SpMin8_Bh(i). These descriptors encode weighted topological and electronic properties and are consistent with the CA I selectivity landscape described above, where compact non-classical carboxylate and coumarin-like scaffolds were enriched. The presence of nArCOOH, nPyridines, C-028, and F03[O-S] among the high-ranking descriptors further suggests that aromatic heterocycles, carboxylate-bearing motifs, and oxygen–sulfur topological relationships contribute to CA I recognition.

For CA II, the most important descriptor was CATS2D_02_AP, followed by N-069, SHED_PL, minsNH2, P_VSA_ppp_P, and sulfur/oxygen-containing fragment descriptors such as F03[O-S]. This profile indicates that CA II activity depends strongly on pharmacophore spacing, heteroatom patterning, sulfonamide-related nitrogen features, and shape/electrostatic distribution. These results are consistent with the classical CA II binding paradigm, in which precise placement of a zinc-binding sulfonamide group and compact aromatic or heteroaromatic tails is central to high-affinity recognition.

For CA IX, the descriptor profile was led by CATS2D_02_AP, SHED_PL, B04[C-S], F04[C-S], N-069, GATS4i, and minsNH2. This pattern supports the dual CA IX selectivity landscape observed in Supplementary Table S2. Pharmacophore-spacing and SHED descriptors are consistent with entrance/rim-directed recognition by non-classical scaffolds, whereas carbon–sulfur and sulfonamide-related descriptors support the importance of hydrophobic or aromatic sulfonamide tails in low-nanomolar CA IX inhibitors. Thus, the CA IX descriptor set captures both non-classical coumarin-type selectivity and classical sulfonamide-based potency.

For CA XII, the most important descriptors were SpMax_AEA(dm), minddssS, P_VSA_s_1, SM10_EA(bo), S-110, SpMax_B(s), MAXDN, mindO, GATS5p, and SpMax3_Bh(s). These features emphasize electronic and topological properties, sulfur-containing fragments, polar surface area, and oxygen/nitrogen interaction potential. This is consistent with the CA XII-selective landscape, where many high-SI compounds retained sulfonamide or sulfamate zinc-binding functionality but achieved selectivity through extended, flexible, heteroaryl, polar–hydrophobic, or sugar-like tail regions compatible with the open CA XII rim.

Overall, Table 2 serves not only as a feature-selection summary but also as a bridge between the experimentally observed selectivity landscape and the subsequent QSAR/XAI interpretation. The descriptor patterns provide isoform-specific pharmacophore hypotheses, while the later model-performance, SHAP, and applicability-domain analyses evaluate whether these descriptor-level signals translate into predictive and chemically interpretable models.

### 2.4. Model Optimization and Evaluation

Four complementary machine-learning regressors were optimized for $pK_{i}$ prediction against each carbonic anhydrase isoform: Random Forest (RF), Generalized Linear Model (GLM), Gradient Boosting (GB), and Artificial Neural Network (ANN). These algorithms were selected to cover linear, tree-based, boosting-based, and neural nonlinear learning strategies. To exploit complementary predictive patterns, a two-level stacked ensemble was then constructed, in which a meta-learner was trained on the cross-validated out-of-fold predictions of the base learners. Hyperparameters were tuned by random search using five-fold cross-validation, with mean residual deviance used as the primary optimization criterion.

**Figure 1:**
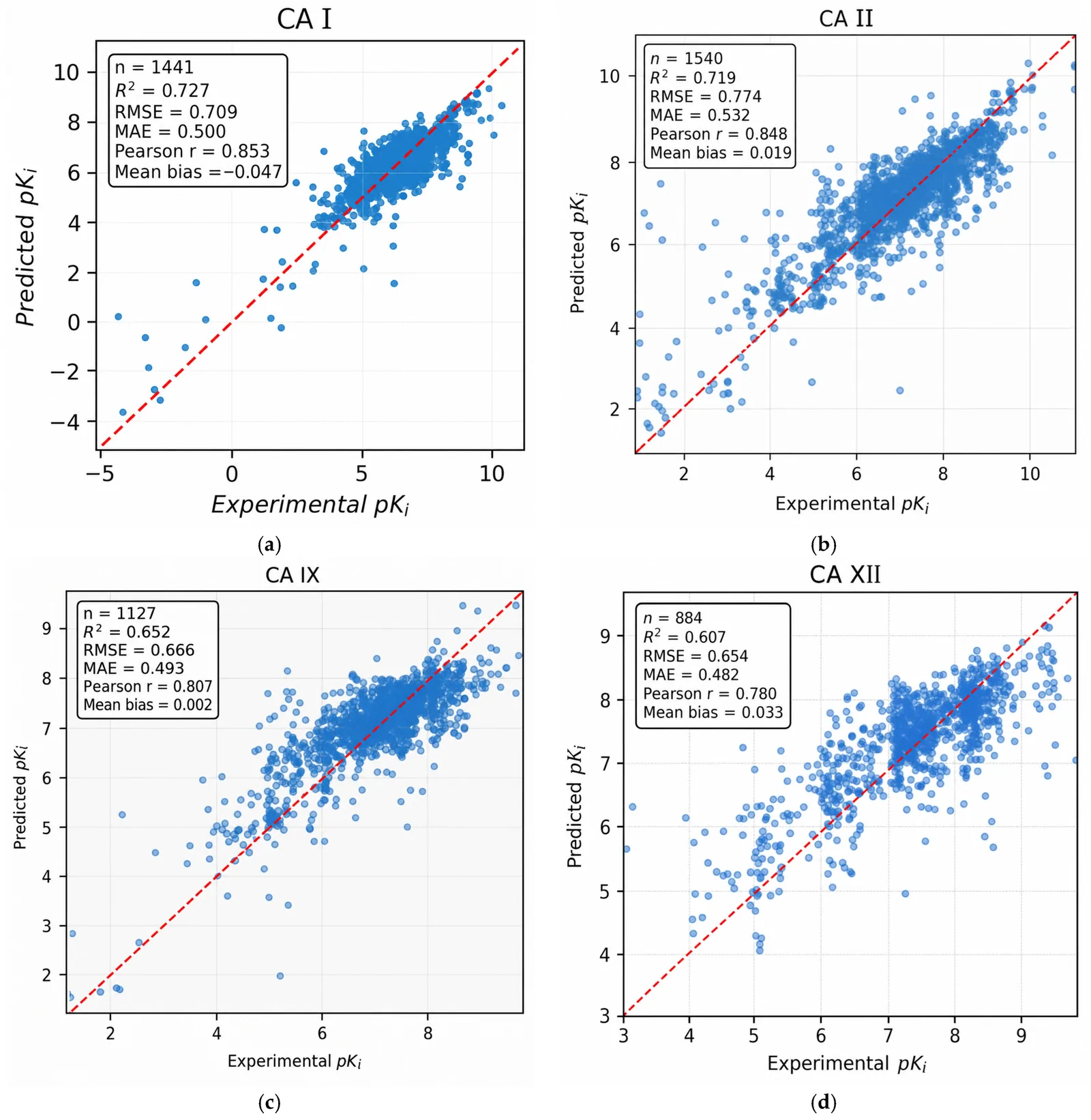
Predicted versus experimental *pK_i_* values for the CA I, CA II, CA IX, and CA XII QSAR test subsets. Scatter plots for (**a**) CA I, (**b**) CA II, (**c**) CA IX, and (**d**) CA XII show predicted versus experimental *pK_i_* values, where $pK_{i}=9-{log}_{10}(K_{i}\;[nM])$. The red dashed line denotes the identity relationship (Predicted = Experimental). Each panel includes the corresponding regression statistics (*n*, R^2^, RMSE, MAE, Pearson’s r, and mean bias) for the held-out test subset.

Model performance was evaluated on the held-out 20% internal test subset for each isoform-specific dataset. Across all four isoforms, the stacked ensemble achieved the best test-set performance among the evaluated models (Table 3, Figure 1). The ensemble reached test-set $R^{2}$ values of 0.727 for CA I, 0.719 for CA II, 0.652 for CA IX, and 0.607 for CA XII, with corresponding RMSE values of 0.709, 0.774, 0.666, and 0.654 $pK_{i}$ units, respectively. These values indicate that the stacked ensemble improved generalization relative to the individual base learners rather than merely reproducing the strongest single model.

Two performance trends were apparent. First, GLM consistently showed the weakest predictive performance, with test-set $R^{2}$ values ranging from 0.242 to 0.382, confirming that the descriptor–activity relationships in these CA inhibitor datasets are substantially nonlinear. In contrast, RF, GB, ANN, and especially the stacked ensemble captured more complex structure–activity patterns. Second, predictive performance varied by isoform. CA I and CA II showed the strongest ensemble performance, consistent with their larger and more structurally populated ChEMBL datasets. CA IX and CA XII showed lower, but still useful, test-set performance, likely reflecting greater chemical heterogeneity, broader scaffold diversity, and the coexistence of classical and non-classical inhibition mechanisms.

The train–test performance gap also warrants careful interpretation. The stacked ensembles showed high training-set $R^{2}$ values, ranging from 0.952 to 0.967, compared with test-set $R^{2}$ values of 0.607 to 0.727. This indicates that the models captured strong structure–activity signals, but also that internal cross-validation and held-out testing are essential to avoid overinterpreting fitted training performance. Therefore, subsequent PubChem experimental-concordance analysis, applicability-domain assessment, SAR/ADME triage, and prioritization for future zinc-aware docking were used as additional post-model safeguards before final candidate selection.

Overall, the stacked ensemble provided the most consistent predictive accuracy across CA I, CA II, CA IX, and CA XII. Its superior internal test-set performance supported its use as the core predictive model for downstream PubChem concordance analysis, virtual screening, selectivity-aware prioritization, applicability-domain stratification, SAR/ADME triage, and future zinc-aware docking and experimental follow-up.

### 2.5. Explainable AI: Global Feature Importance and Local Interpretability

To improve the interpretability of the stacked-ensemble QSAR models, an explainable AI (XAI) framework was applied at two complementary levels: permutation feature importance (PFI) to identify descriptors that most strongly affected model performance at the population level, and SHAP analysis to examine descriptor contributions for the top predicted selective inhibitors at the compound-set level. Together, these analyses tested whether the models relied on chemically meaningful descriptors rather than purely statistical correlations.

#### 2.5.1. Global Feature Importance via Permutation Analysis

**Figure 2:**
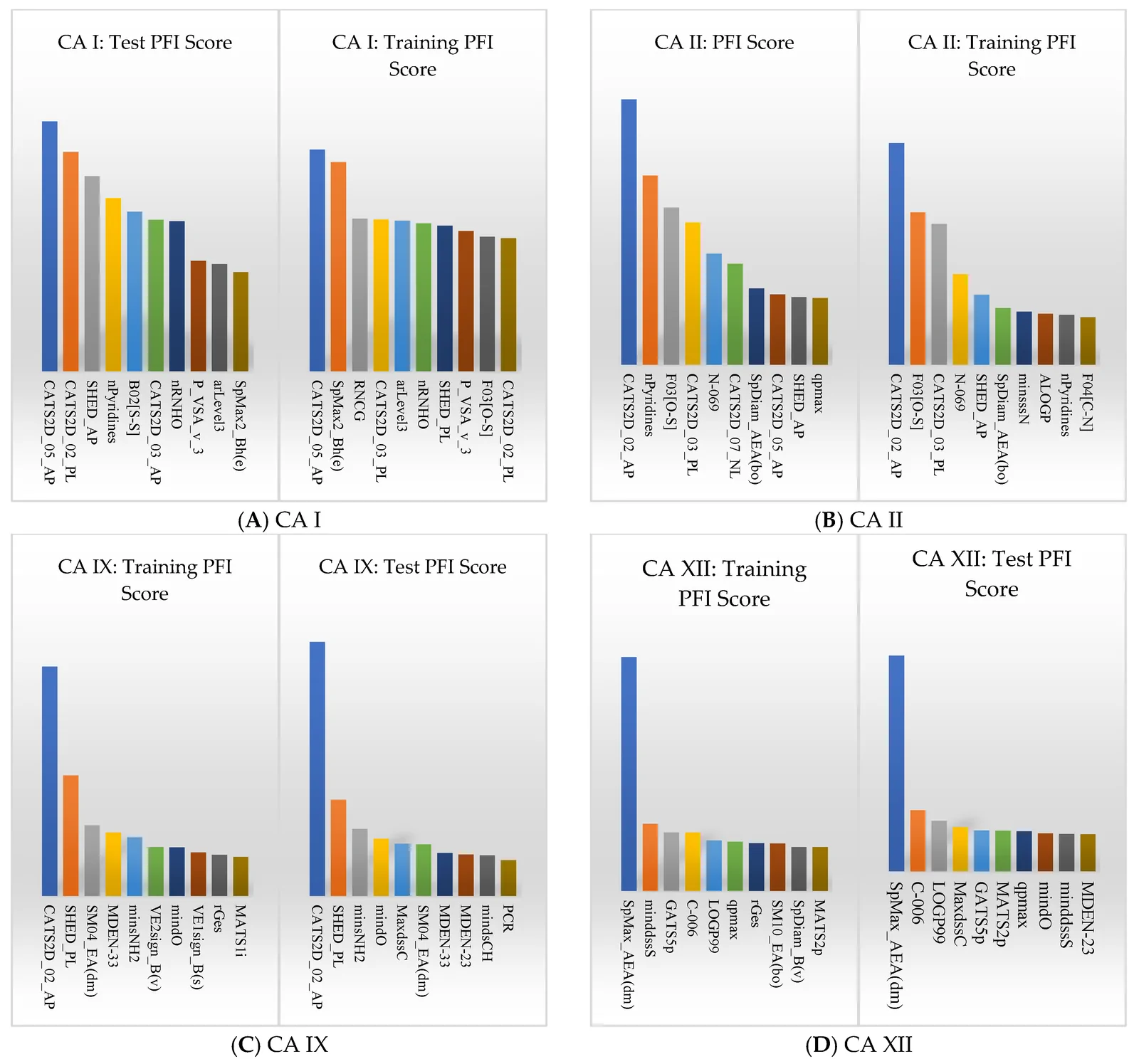
Global permutation feature importance for the CA I, CA II, CA IX, and CA XII models. The top-ten descriptors are shown for each isoform model using both training and held-out test subsets. Comparison of training- and test-subset importance highlights descriptors that were consistently influential during model fitting and generalization. Bar colors are used only for visual clarity and do not denote descriptor classes or additional categorical information.

PFI was calculated for both training and held-out test subsets to distinguish descriptors important for fitted model behavior from those that remained important under generalization—a distinction that matters because training-subset importance alone can reflect overfitting, whereas retained importance in the test subset provides stronger evidence of a generalizable structure–activity signal. The top-ten descriptors per isoform are summarized in Figure 2.

For CA I, CATS2D_05_AP was the most influential descriptor in both subsets (PFI: 0.021 training, 0.039 test), indicating that atom-pair pharmacophore spacing contributed consistently to *pK_i_* prediction. SpMax2_Bh(e) and RNCG were influential in training but shifted in relative importance on the held-out set, suggesting only part of the CA I descriptor signal was fully stable across partitions—consistent with the structurally mixed CA I landscape, which spans both compact non-classical scaffolds and classical inhibitor motifs.

CA II showed stronger train–test consistency: CATS2D_02_AP and F03[O-S] ranked among the most important descriptors in both subsets, supporting a chemically plausible reading in which CA II activity depends on pharmacophore spacing and oxygen–sulfur atom-pair arrangements—features consistent with sulfonamide/sulfamate chemotypes and their precise orientation within the narrow CA II pocket, and directly echoing the canonical Zn^2+^-anchoring/compact-tail pattern established in Section 2.2.

For CA IX, CATS2D_02_AP and SHED_PL were the most stable descriptors, with CATS2D_02_AP scoring highly in both subsets—indicating reliance on pharmacophore geometry and shape/electrostatic distribution, chemically coherent with the dual CA IX selectivity pattern documented in Supplementary Table S2 (non-classical coumarin-like scaffolds needing entrance/rim complementarity, versus classical sulfonamide inhibitors needing hydrophobic or aromatic tails compatible with the wider CA IX rim).

CA XII showed the broadest and most train–test-variable profile: SpMax_AEA(dm) remained consistently important, while C-006 and LOGP99 gained relative weight in the test subset—implying that CA XII prediction draws on a wider combination of topological, electronic, lipophilicity, and atom-centered fragment descriptors, consistent with the open, solvent-exposed CA XII rim accommodating chemically diverse scaffolds with extended polar–hydrophobic or flexible tails.

Overall, PFI confirmed that no single descriptor family dominated across isoforms; each showed a distinct dependence profile broadly consistent with both the structural selectivity landscape (Section 2.2) and the RF-selected descriptor patterns (Table 2).

#### 2.5.2. Local Interpretability Using SHAP Analysis for Top Selective Inhibitors

**Figure 3:**
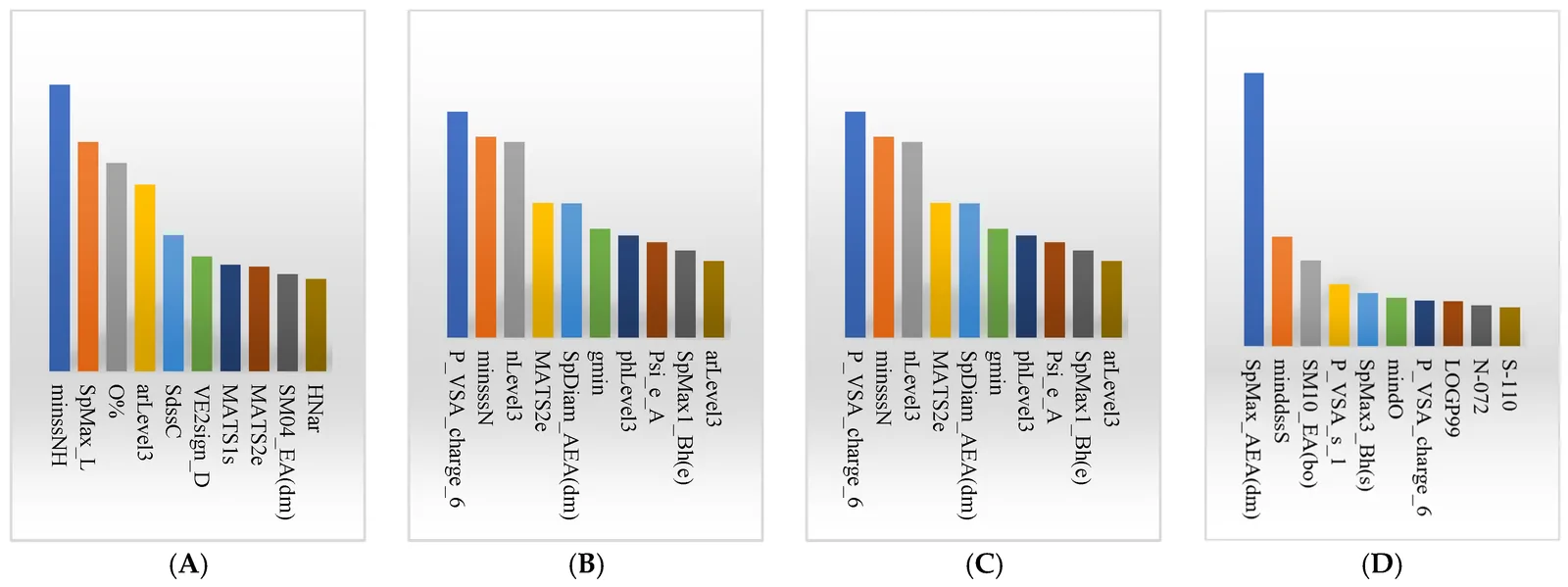
SHAP-based local interpretability analysis for the top-ten predicted CA I-selective inhibitors. Mean absolute SHAP values are shown for the CA I candidate set evaluated across (**A**) the CA I model, (**B**) the CA II model, (**C**) the CA IX model, and (**D**) the CA XII model. Bar colors are used only for visual clarity and do not denote descriptor classes, model categories, or additional quantitative information.

**Figure 4:**
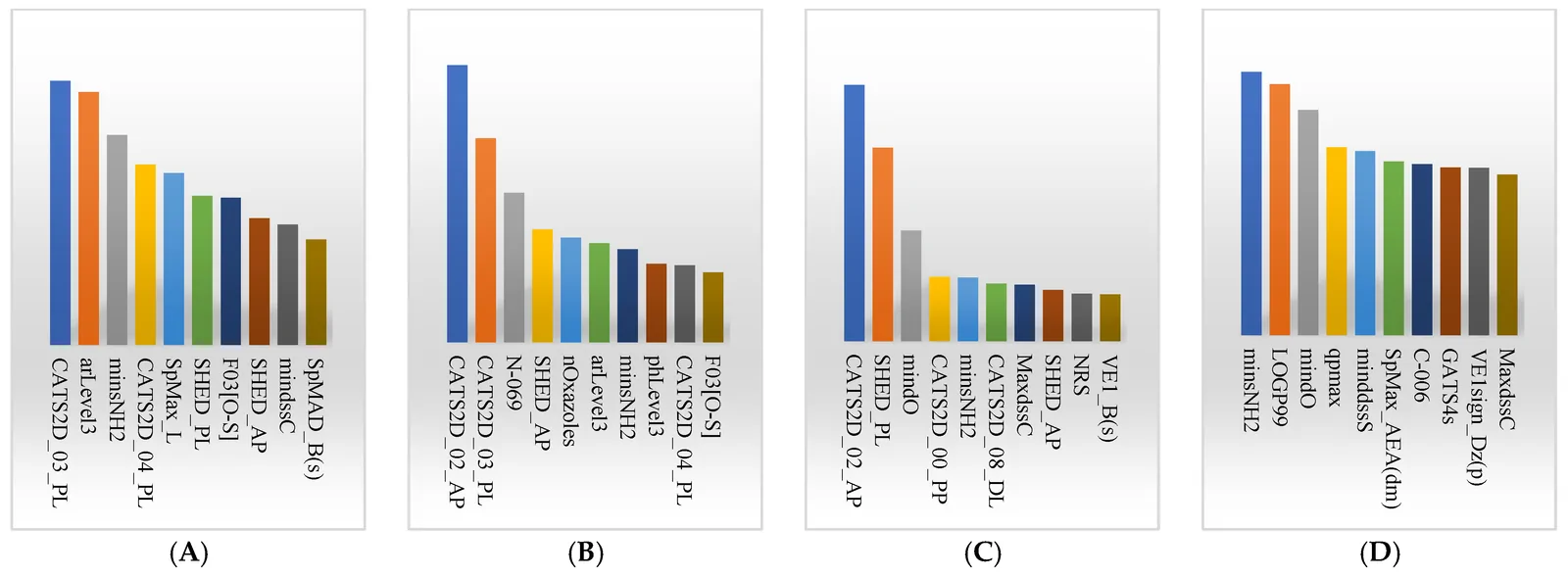
SHAP-based local interpretability analysis for the top-ten predicted CA II-selective inhibitors. Mean absolute SHAP values are shown for the CA II candidate set evaluated across (**A**) the CA I model, (**B**) the CA II model, (**C**) the CA IX model, and (**D**) the CA XII model. Bar colors are used only for visual clarity and do not denote descriptor classes, model categories, or additional quantitative information.

**Figure 5:**
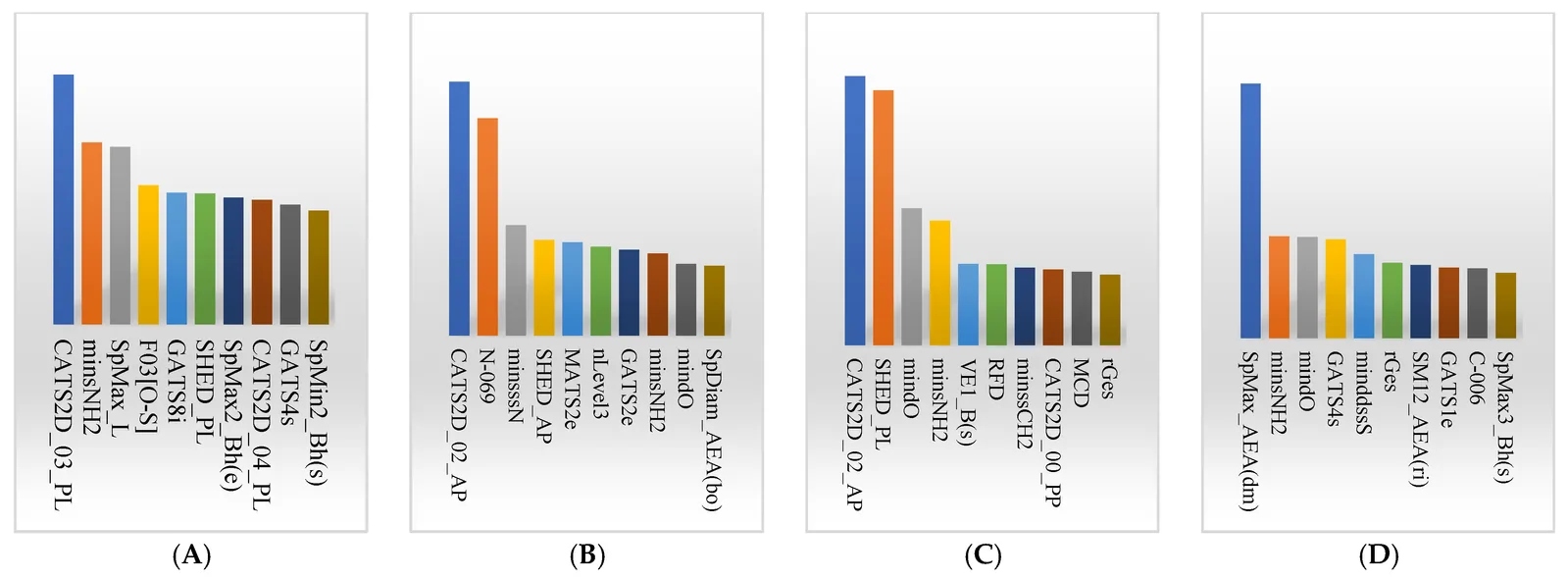
SHAP-based local interpretability analysis for the top-ten predicted CA IX-selective inhibitors. Mean absolute SHAP values are shown for the CA IX candidate set evaluated across (**A**) the CA I model, (**B**) the CA II model, (**C**) the CA IX model, and (**D**) the CA XII model. Bar colors are used only for visual clarity and do not denote descriptor classes, model categories, or additional quantitative information.

**Figure 6:**
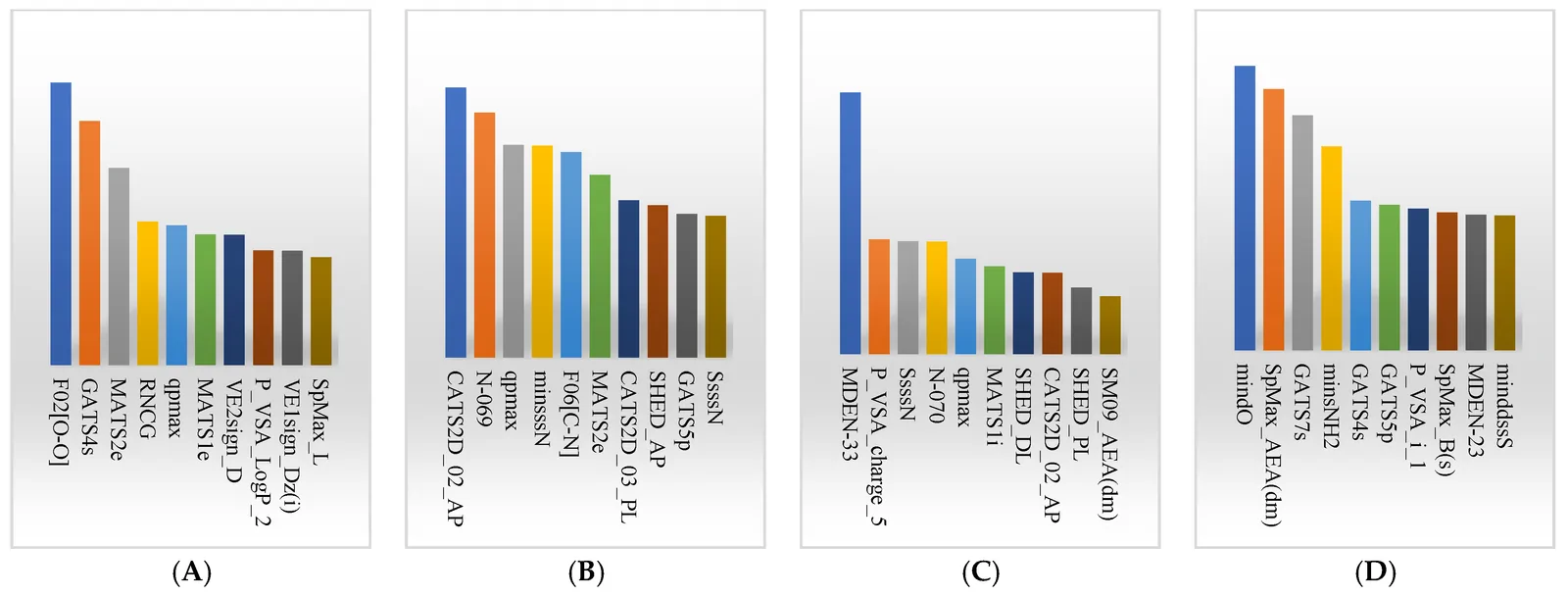
SHAP-based local interpretability analysis for the top-ten predicted CA XII-selective inhibitors. Mean absolute SHAP values are shown for the CA XII candidate set evaluated across (**A**) the CA I model, (**B**) the CA II model, (**C**) the CA IX model, and (**D**) the CA XII model. Bar colors are used only for visual clarity and do not denote descriptor classes, model categories, or additional quantitative information.

SHAP analysis was applied to the top-ten predicted selective inhibitors per CA isoform, with each compound set evaluated against its target model and the three off-target models—enabling direct comparison of which descriptors drove predicted *pK_i_* for the target isoform versus the off-targets. For each isoform-specific set, the top-ten descriptors by mean absolute SHAP value were extracted and visualized in Figure 3, Figure 4, Figure 5 and Figure 6. Because mean absolute SHAP values quantify magnitude of influence only, directional interpretation (whether a descriptor increases or decreases predicted *pK_i_*) requires signed SHAP values, summary plots, or dependence plots—not the magnitude rankings presented here.

For CA I-selective candidates (Figure 3), influential descriptors in the CA I target model included hydrogen-bonding, oxygen-content, and atom-centered features (minssNH, O%), whereas the off-target models, particularly the CA XII model in Figure 3D, emphasized topological/electronic, sulfur-containing, polar-surface, charge, and lipophilicity-related descriptors. This pattern suggests that CA I-selective predictions track compact polar/hydrogen-bonding character, distinct from the broader shape, sulfur/oxygen, charge, and lipophilicity-driven signals used by the off-target models.

For CA II-selective candidates (Figure 4), the CA II model emphasized pharmacophore and heteroatom descriptors (CATS2D_02_AP, N-069), consistent with the classical CA II recognition pattern in which sulfonamide-related nitrogen features and precise atom-pair geometry drive high-affinity binding. Off-target models weighted different descriptor groups for the same compounds, indicating that CA II-optimized chemotypes are read differently by the CA I, CA IX, and CA XII models.

For CA IX-selective candidates (Figure 5), CATS2D_02_AP and SHED_PL were prominent, pointing to pharmacophore spacing and shape/electrostatic distribution as key to predicted CA IX activity—consistent with CA IX’s capacity to accommodate larger, more hydrophobic entrance-directed chemotypes than CA I or CA II. Off-target models emphasized size, charge distribution, and fragment composition instead, reflecting the physicochemical trade-offs underlying CA IX selectivity.

For CA XII-selective candidates (Figure 6), the SHAP profile was broader and less single-motif-dependent than those of CA II and CA IX. Sulfur-containing, polar, lipophilicity-related, and topological descriptors contributed across the target and off-target models, consistent with the open CA XII rim, its broader tail-accommodation capacity, and the greater scaffold diversity observed among CA XII-selective compounds in Supplementary Table S2.

#### 2.5.3. Integrated Isoform-Specific and Chemical Interpretation

Combining global PFI with local SHAP analysis provided a coherent descriptor-level explanation of isoform-selective CA inhibition. The global PFI results identified the descriptors most important for overall model performance, whereas the SHAP analysis showed how descriptor influence varied across the top predicted selective inhibitors and across the corresponding off-target models. Together, these analyses indicate that isoform selectivity is not governed by a single universal descriptor class, but by distinct combinations of topology, polarity, heteroatom patterning, sulfur/oxygen atom-pair relationships, lipophilicity, pharmacophore spacing, and molecular shape.

The resulting interpretation mapped closely onto the rim-region selectivity model described in Section 2.2. CA I predictions were associated with compact topology, oxygen content, hydrogen-bonding descriptors, and non-classical polar features, consistent with the narrower and more rigid CA I active-site entrance and the enrichment of compact carboxylate/coumarin-like chemotypes in the CA I-selective subset. CA II predictions depended strongly on pharmacophore-pair and heteroatom descriptors compatible with sulfonamide-type Zn^2+^ anchoring and compact aromatic or heteroaromatic tail positioning, agreeing with the structurally constrained, Phe131-lined CA II entrance and the dominance of classical sulfonamide chemotypes among its most selective inhibitors. CA IX predictions emphasized pharmacophore spacing, shape/electrostatic descriptors, and hydrophobic or sulfur-containing fragment patterns, consistent with a wider and more hydrophobic entrance able to support both non-classical entrance-directed scaffolds and hydrophobic-tailed sulfonamide inhibitors. CA XII predictions involved sulfur-containing, polar, lipophilicity-related, and topological descriptors, matching the broader tail-accommodation capacity of its open, solvent-exposed, more polar rim.

These results suggest that predicted selectivity arises from two complementary effects. First, certain descriptors support high predicted affinity for the target isoform by encoding favorable pharmacophore geometry, zinc-binding compatibility, tail topology, or entrance/rim complementarity. Second, the same compounds are read differently by the off-target models, whose predictions draw on physicochemical or topological signals poorly matched to those isoforms’ binding environments. Selectivity therefore emerges not only from features that improve target binding, but from features that actively reduce predicted off-target affinity.

An important limitation is that the SHAP analyses in Figure 3, Figure 4, Figure 5 and Figure 6 were summarized using mean absolute SHAP values. They therefore identify the descriptors with the greatest explanatory influence but do not by themselves establish whether each descriptor increases or decreases predicted *pK_i_*. Directional effects require signed SHAP summary plots, dependence plots, or compound-level signed SHAP inspection—analyses that would be particularly valuable at the next stage, for candidate-level SAR refinement where specific substituent modifications could be assessed for predicted effects on target potency and off-target liability.

Overall, the XAI framework provides more than a retrospective explanation of model behavior: it creates a chemically interpretable bridge between the experimental selectivity landscape in Supplementary Table S1, the compound-level selectivity evidence in Supplementary Table S2, the RF-selected descriptor profiles in Table 2 and Supplementary Table S3, and the subsequent virtual-screening prioritization. The results broadly support the established tail-based strategy for CA inhibitor design—most clearly for CA II and CA XII—while also confirming CA IX’s dual character (both tail-optimized classical inhibitors and non-classical entrance-directed scaffolds) and highlighting compact non-classical opportunities specific to CA I. Thus, the integrated XAI analysis provides a transparent computational basis for prioritizing selective CA inhibitor candidates for applicability-domain assessment, docking, and future experimental validation.

### 2.6. Prospective Virtual Screening and PubChem-Based External Experimental Concordance Across Four CA Isoforms

For the CA I-, CA II-, CA IX-, and CA XII-prioritized PubChem shortlists, the top-1000 candidates per isoform were queried against PubChem assay summaries to retrieve available experimental bioactivity records. Returned records were curated by endpoint and target identity. Only numeric *K_i_* records mapped to the corresponding human CA isoform were used for predicted-versus-experimental *pK_i_* concordance. Other endpoints, including *IC*_50_, *K_d_*, potency, selectivity ratios, and screening outcomes, were retained only as supporting bioactivity annotations and were not used for *K_i_* concordance analysis or selectivity-index calculations. The four shortlists comprised 4000 unique prioritized candidates in total, with no shared compounds among the isoform-specific top-1000 lists, as documented in Supplementary Table S4.

Target identity was curated by mapping each PubChem Target_Accession field to the corresponding human isoform: CA I, P00915; CA II, P00918; CA IX, Q16790; and CA XII, O43570. Records against non-human orthologs, unrelated targets, non-CA proteins, non-UniProt structural identifiers, or non-numeric activity annotations were excluded from the concordance analysis, although they were retained in the raw evidence table for transparency. When multiple redundant *K_i_* records were available for the same compound–isoform pair, the median experimental activity value was used as the representative measurement. Experimental *K_i_* values reported in PubChem as µM were converted to *pK_i_* as:

$$pK_{i}=6-{log}_{10}(K_{i}[\mu M])$$

whereas QSAR-predicted *K_i_* values reported in nM were converted as:

$$pK_{i}=9-{log}_{10}(K_{i}[nM])$$

Of the 4000 prioritized candidates queried, 1098 returned at least one PubChem bioassay record of any kind, while 2902 had no returned PubChem bioactivity record. Among the 1098 compounds with returned records, 847 carried at least one numeric direct *K_i_* value against one of the four modeled human CA isoforms and were retained for predicted-versus-experimental concordance analysis. The remaining 251 compounds returned only non-*K_i_* endpoints, non-matching targets, non-human isoform records, or non-numeric activity annotations and were excluded from concordance calculations while remaining available as supporting bioactivity context. Overall, 428 compounds had direct *K_i_* records against all four modeled isoforms, 419 had partial direct *K_i_* coverage against one to three isoforms, and 3153 had no direct *K_i_* record against any of the four modeled CA isoforms. Consequently, 3572 of the 4000 prioritized candidates lacked complete four-isoform experimental *K_i_* coverage and remain priority targets for future experimental characterization.

**Figure 7:**
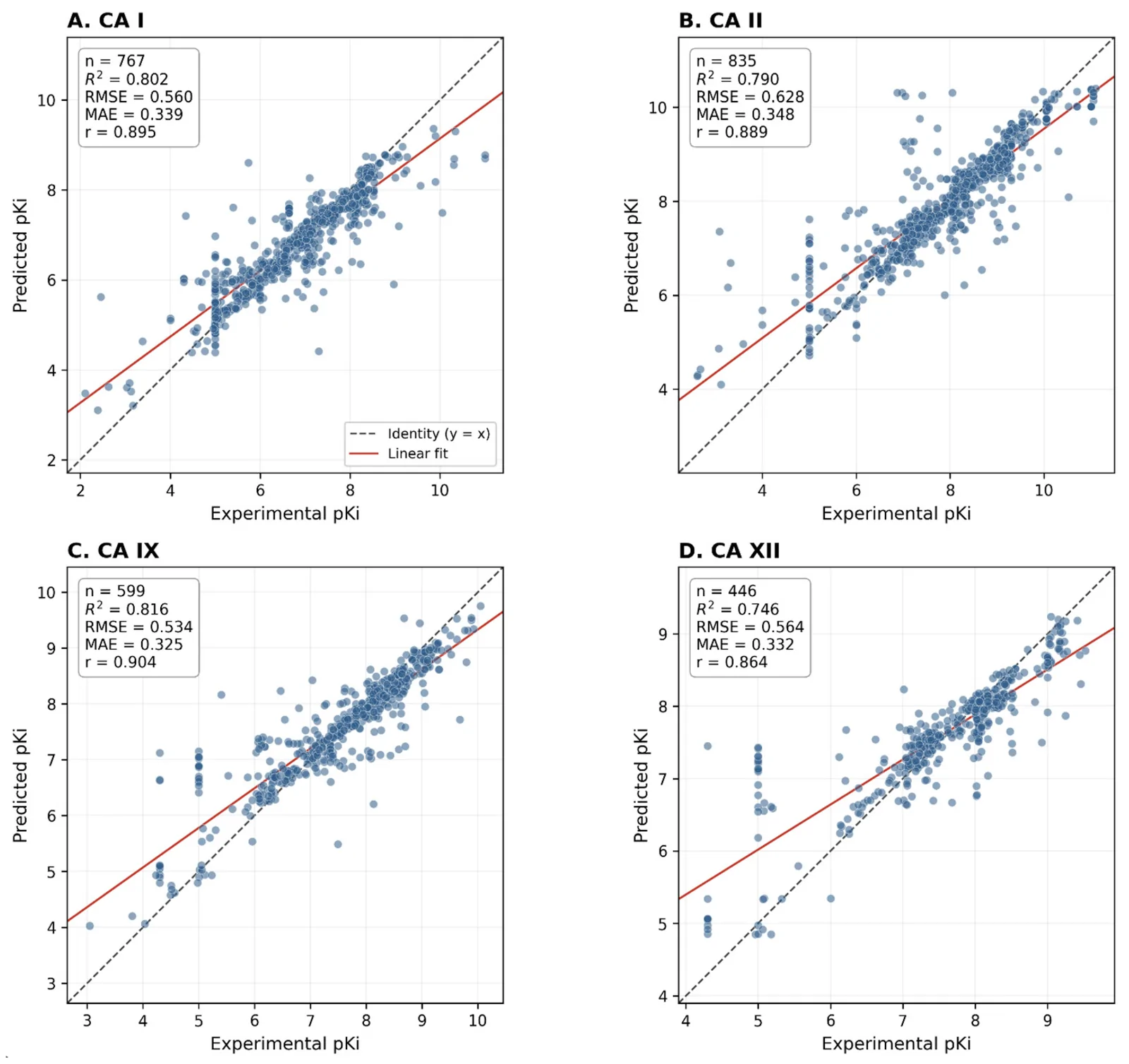
External PubChem experimental concordance of the CA I, CA II, CA IX, and CA XII QSAR models. Predicted versus experimental *pK_i_* plots are shown for (**A**) CA I, (**B**) CA II, (**C**) CA IX, and (**D**) CA XII. Experimental Ki records were retrieved from PubChem and curated to include only numeric *K_i_* values mapped to the corresponding human CA isoform. The dashed line represents the identity relationship between predicted and experimental *pK_i_*, and the solid line represents the linear fit. Each panel reports the number of compounds, R^2^, RMSE, MAE, and Pearson correlation coefficient.

Predicted-versus-experimental concordance was evaluated in *pK_i_* units for matched compound–isoform pairs only. Thus, predicted CA I *pK_i_* values were compared only with experimental CA I *K_i_* records, predicted CA II only with CA II records, predicted CA IX only with CA IX records, and predicted CA XII only with CA XII records. The resulting PubChem-based concordance analysis showed substantial agreement across all four isoforms (Table 4; Figure 7; Supplementary Table S4).

Across all four isoforms, Pearson correlations exceeded 0.86 and linear-fit R^2^ values exceeded 0.74, with MAE values below 0.35 *pK_i_* units. This corresponds to an average error of approximately two-fold in *K_i_*, which is reasonable for independent PubChem-derived bioactivity records aggregated from heterogeneous assay sources. The comparatively lower R^2^ and higher RMSE observed for CA XII are consistent with its broader and more chemically diverse selective-inhibitor landscape described in Section 2.2, which may pose a greater generalization challenge for descriptor-based prediction than the more structurally constrained CA I and CA II chemical spaces. Importantly, PubChem experimental *K_i_* values were used only for external concordance and evidence classification. They were not used to calculate predicted SI, Δ *pK_i_*, or candidate priority ranks. Candidate novelty was therefore assigned at the compound–isoform level. A compound was considered experimentally supported for a given isoform if a direct PubChem *K_i_* record was available for that same human isoform; otherwise, it was considered isoform-level novel for that target, even if experimental records existed for other isoforms or unrelated targets. This distinction preserves the prospective nature of the virtual-screening prioritization and enables downstream selection of novel candidates for SAR interpretation, ADME profiling, docking, and future experimental validation.

### 2.7. SAR Triage and ADME-Guided Prioritization of Novel and Partially Novel Candidates

Building on the experimental-evidence classification established in Section 2.6, the 3572 prioritized candidates lacking complete four-isoform experimental *K_i_* coverage were carried forward for structure–activity and pharmacokinetic triage. This candidate pool comprised 3153 compounds with no direct *K_i_* evidence against any of the four modeled isoforms and 419 compounds with partial *K_i_* coverage against one to three isoforms (Supplementary Table S5). As defined throughout the workflow, experimental PubChem evidence was used only to determine validation and novelty status. Candidate ranking and prioritization were based exclusively on QSAR-predicted potency, predicted selectivity index, and downstream SAR/ADME assessment.

For each isoform, target-novel candidates were defined as compounds prioritized within the corresponding isoform-specific PubChem shortlist but lacking direct experimental *K_i_* evidence for that same isoform. These candidates were ranked by combined predicted potency and predicted selectivity, deduplicated by structure to remove redundant PubChem records representing the same compound, and reduced to the ten highest-priority candidates per isoform (Supplementary Table S6A). RDKit-assisted substructure annotation, followed by medicinal-chemistry SAR review, revealed isoform-specific chemotype patterns consistent with the structural selectivity landscape described in Section 2.2.

Among the CA I top-ten candidates, none carried a primary sulfonamide zinc-binding group. Instead, the CA I shortlist was enriched in non-classical chemotypes, including thiourea/thioamide-containing scaffolds, a carboxylic acid, aryl-hydrazone/polyaryl structures, and heteroaryl/aromatic amide-like motifs. This pattern suggests that the CA I model prioritized compact or entrance-compatible non-classical scaffolds rather than canonical sulfonamide zinc binders. However, because these chemotypes are structurally distinct from much of the classical CA inhibitor training space, their predicted CA I activity should be interpreted as novelty-oriented ligand-based hypotheses rather than definitive evidence of a validated CA I binding mode.

In contrast, all ten CA II and all ten CA IX top candidates carried a primary sulfonamide group, consistent with classical Zn^2+^-anchored CA inhibition. This result is chemically plausible because the CA II and CA IX candidate sets were dominated by sulfonamide-bearing scaffolds in which potency is expected to depend on catalytic Zn^2+^ coordination, while isoform preference is modulated by scaffold architecture and tail compatibility with active-site rim regions. The CA XII candidates were more structurally heterogeneous, combining classical sulfonamide/sulfamate scaffolds with non-classical carboxylate- and amide-containing chemotypes.

SAR review identified two CA XII candidates, PubChem CID 175661362 and CID 57773653, bearing a sulfonyl chloride group rather than a stable sulfonamide or sulfamate zinc-binding group. Sulfonyl chlorides are hydrolytically reactive electrophilic substituents and are unlikely to represent stable final biological hit structures under aqueous or biological conditions. Accordingly, these two compounds were flagged as reactive-liability structures and retained only as analogue-design templates rather than viable biological candidates. A further CA XII candidate, CID 4460361, was retained with a mechanistic caution. Although it showed favorable predicted potency, its putative zinc-binding motif is a non-canonical sulfonate ester/sulfonate-like group rather than a primary sulfonamide or sulfamate. Because this motif lacks the sulfonamide N–H typically involved in canonical CA Zn^2+^ coordination, its predicted activity was treated as provisional pending zinc-aware docking and experimental confirmation.

The 40 SAR-triaged candidates were subsequently profiled using SwissADME to calculate physicochemical descriptors, drug-likeness rule compliance, PAINS and Brenk structural alerts, predicted gastrointestinal absorption, bioavailability score, and synthetic accessibility. These outputs were combined into a heuristic 0–100 medicinal-chemistry score to support developability ranking (Supplementary Table S6B). Importantly, ADME profiling did not override structure-based exclusions. Both sulfonyl chloride-bearing CA XII candidates retained acceptable physicochemical profiles and ADME scores of 75/100 despite their clear chemical-reactivity liability. This confirms that descriptor-based drug-likeness scoring alone cannot substitute for SAR-based structural-alert review.

SwissADME profiling also identified independent developability liabilities that were not apparent from QSAR potency or selectivity ranking alone. Three CA XII candidates, CID 227581, CID 3917216, and CID 4993108, had ranked favorably by predicted potency and selectivity but returned very poor ADME scores of 0–7/100. These low scores were driven by poor predicted gastrointestinal absorption, two Lipinski and two Veber violations, excessive polarity and/or flexibility, predicted P-glycoprotein substrate behavior, and low predicted bioavailability scores of 0.17. These candidates were therefore deprioritized despite favorable predicted binding profiles, illustrating that QSAR/SAR ranking alone is insufficient to identify practical developability liabilities.

Combining QSAR-predicted potency, predicted selectivity, SAR plausibility, structural-alert review, and SwissADME scoring, while excluding the two reactivity-flagged sulfonyl chloride candidates, yielded a final panel of five prioritized candidates per isoform for downstream applicability-domain confirmation and zinc-aware docking validation (Table 5). This integrated triage prevented the workflow from becoming a potency-only ranking exercise and instead produced a chemically more realistic candidate panel for subsequent mechanistic and experimental validation.

The very high predicted CA XII selectivity index for CID 155553321 (*SI* = 9260.5) should be interpreted with caution. In this workflow, SI was calculated entirely from model-predicted *K_i_* values as the ratio between the most sensitive predicted off-target isoform and the predicted target isoform. Therefore, an extreme SI value can arise from the combination of strong predicted CA XII potency and a much weaker predicted *K_i_* against the limiting off-target, rather than from a uniquely validated isoform-discriminating binding profile. Although CID 155553321 showed favorable AD support and was retained as a high-priority CA XII candidate, the absolute magnitude of this SI value should not be treated as an experimentally confirmed selectivity margin. Instead, it indicates a strong model-derived preference for CA XII that warrants direct four-isoform biochemical validation before the reported SI is interpreted quantitatively.

### 2.8. Applicability-Domain Analysis of Test Subsets and Final Prioritized Candidates

Beyond PubChem-based experimental concordance (Section 2.6), the reliability of the four isoform-specific QSAR predictions was further assessed through applicability-domain (AD) analysis. The AD analysis was performed independently for the CA I, CA II, CA IX, and CA XII models and was designed to determine whether both held-out test compounds and final prioritized PubChem candidates fell within the chemical and descriptor space learned by each model.

For each isoform, three complementary AD criteria were combined: Morgan fingerprint similarity to the corresponding training set, standardized descriptor-space distance to the five nearest training compounds, and leverage analysis. Morgan fingerprints were calculated using radius 2 and 2048 bits, and each compound was characterized by its maximum Tanimoto similarity and mean five-nearest-neighbor Tanimoto similarity relative to the training set. Descriptor-space proximity was quantified as *D_5NN_*, defined as the mean standardized Euclidean distance to the five nearest training compounds using the final 100 predictive descriptors for each isoform. Leverage was calculated as:

$$h_{i}=x_{i}^{T}(X^{T}X{)}^{-1}x_{i}$$

with the warning threshold defined as:

$$h^{*}=\frac{3(p+1)}{n}$$

where $x_{i}$ is the descriptor vector of compound i, X is the training descriptor matrix, p is the number of final predictive descriptors, and n is the number of training compounds. This combined AD strategy was used because fingerprint similarity alone may not capture extrapolation in descriptor space, whereas $D_{5NN}$ and leverage provide complementary information on multivariate descriptor-domain membership.

AD thresholds were calibrated separately for each isoform using the held-out test subset. Absolute prediction error was calculated as:

$$AE=\;|pK_{i}^{predicted}-pK_{i}^{experimental}|$$

Low-error test compounds were defined as those with AE≤0.50 *pK_i_*, while acceptable-error compounds were defined as those with AE≤1.00 *pK_i_*. The high-confidence fingerprint threshold was defined as the median maximum Tanimoto similarity among low-error test compounds, whereas the moderate-confidence fingerprint threshold was defined as the 5th percentile among acceptable-error compounds. Descriptor-distance thresholds were derived analogously, using the median $D_{5NN}$ among low-error compounds and the 95th percentile $D_{5NN}$ among acceptable-error compounds. Leverage was interpreted relative to the isoform-specific warning threshold $h^{*}$.

The three normalized AD components were integrated into a composite score:

AD Score=0.45×Fingerprint Score+0.35×Descriptor Distance Score+0.20×Leverage Score

Compounds were classified as very high AD when AD Score≥0.80, high AD when 0.65≤AD Score<0.80, moderate AD when 0.45≤AD Score<0.65, and low/out-of-domain when AD Score<0.45. These classes were used as confidence labels rather than exclusion filters. Thus, low-AD candidates were not automatically discarded, but were retained as extrapolative or novelty-oriented hypotheses requiring stronger downstream validation.

Table 6 reports held-out test-subset prediction error stratified by AD class for each isoform. Across all four models, prediction error increased consistently from the very-high-AD class to the low/out-of-domain class. This trend supports the composite AD score as a useful reliability annotation rather than an arbitrary chemical-space filter. The AD analysis therefore provides an additional interpretive layer for final candidate prioritization by distinguishing model-supported predictions from structurally or descriptor-space extrapolative hypotheses.

Candidate-level AD analysis showed substantial isoform-dependent differences in prediction confidence (Table 7). For CA I, all five final candidates were classified as low/out-of-domain according to the composite AD score. CID 142163499 showed the strongest AD support within the CA I set, but none of the five candidates reached moderate confidence. This pattern is consistent with the CA I shortlist being enriched in non-classical chemotypes, including thiourea/thioamide, hydrazone, heteroaryl, and carboxylate-containing scaffolds, rather than canonical primary sulfonamide zinc binders. These compounds therefore represent novelty-oriented CA I hypotheses, but their predicted potency should be interpreted cautiously because they extrapolate beyond the best-supported region of the CA I training domain.

For CA II, three of the five candidates—CID 110412583, CID 155812825, and CID 9821692—were classified as very high AD, indicating strong support from the CA II training chemical space. Two of these compounds showed a maximum Morgan fingerprint Tanimoto similarity of 1.000 to the training set; this should be interpreted as identity at the selected fingerprint representation level, not necessarily as proof of exact structural identity. CID 21735834 was classified as moderate AD and may be advanced with caution. CID 130448357 was classified as low/out-of-domain, mainly because of high descriptor-space distance ($D_{5NN}=8.564$) and leverage marginally exceeding the CA II warning threshold (h=0.057 versus $h^{*}=0.049$).

For CA IX, AD support was mixed. CID 21051322 and CID 169223349 were classified as very high AD, supported by high fingerprint similarity to CA IX training compounds, with maximum Tanimoto values of 0.830 and 1.000, respectively. These two compounds therefore represent the most model-supported CA IX candidates for downstream validation. CID 114317887 was classified as moderate AD, whereas CID 418959 and CID 419060 were classified as low/out-of-domain despite favorable predicted potency and SAR profiles. Their lower AD classification reflects reduced fingerprint similarity to the CA IX training space and increased descriptor-space extrapolation.

For CA XII, the final candidate panel showed strong overall AD support. Three candidates—CID 155553321, CID 4460361, and CID 155516037—were classified as very high AD, while CID 155567815 and CID 4289218 were classified as moderate AD. CID 4460361 requires specific interpretation: its high AD classification indicates that the ligand is well supported within the learned CA XII chemical space, but this is a ligand-similarity and descriptor-domain statement, not a binding-mode confirmation. Because CID 4460361 contains a previously flagged non-canonical sulfonate ester/sulfonate-like motif rather than a validated primary sulfonamide or sulfamate zinc-binding group (Section 2.7), its predicted activity should not be interpreted as evidence of canonical Zn^2+^ coordination. That mechanistic question requires zinc-aware docking and experimental inhibition testing.

Integrated Interpretation: Across all four isoforms, test-set prediction error increased consistently from very-high AD to low/out-of-domain AD classes, supporting the composite AD score as a useful reliability annotation rather than an arbitrary chemical-space filter. Candidate-level AD support was strongest overall for CA II and CA XII, intermediate for CA IX, and weakest for CA I. The CA II and CA XII panels therefore provide the most robust ligand-based starting points for downstream validation, while the CA IX panel contains two strongly supported candidates and three candidates requiring greater caution. In contrast, the CA I candidates should be framed primarily as exploratory, non-classical design hypotheses rather than high-confidence in-domain predictions. Importantly, AD classification refines confidence but does not establish binding mechanism. A compound may be in-domain according to fingerprint, descriptor-distance, and leverage criteria while still carrying a mechanistic caveat, as illustrated by CID 4460361. Conversely, a low-AD compound may remain chemically interesting as a novelty-oriented scaffold but requires stronger downstream evidence before activity or selectivity claims are made. Thus, AD analysis should be interpreted together with SAR review, SwissADME profiling, zinc-aware docking, molecular dynamics, and experimental *K_i_* validation.

**Figure 8:**
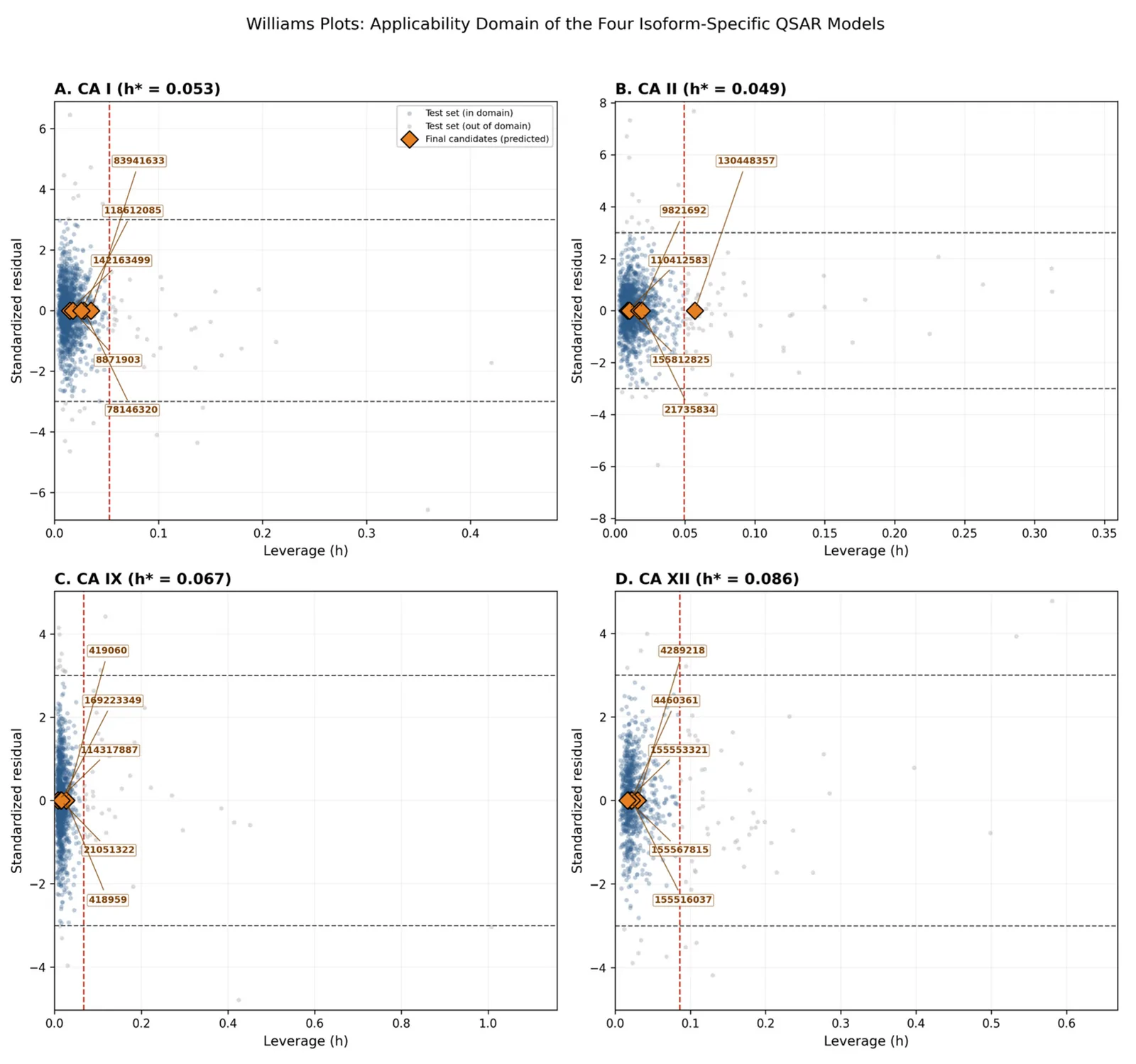
Williams plots of the applicability domain for the four isoform-specific QSAR models. (**A**) CA I, (**B**) CA II, (**C**) CA IX, and (**D**) CA XII. Blue points represent held-out test compounds within the leverage/residual applicability region, whereas grey points represent test compounds outside this region. Dashed horizontal lines indicate the conventional standardized residual limits of ±3, and the dashed red vertical line indicates the isoform-specific leverage warning threshold $h^{*}$. Orange diamonds represent the five final prioritized candidates for each isoform. Because these candidates lack experimental *pK_i_* values, they are plotted at standardized residual = 0 only to visualize their leverage positions; this placement does not represent prediction error. Candidate AD class assignments in Table 7 were based on the full composite AD score, not on Williams-plot leverage alone.

Williams Plot Visualization of the Applicability Domain: To visualize the applicability-domain analysis, Williams plots were constructed for each isoform using leverage versus standardized residuals for the held-out test subset, with the five final prioritized candidates overlaid (Figure 8). These plots provide a useful leverage-based visualization of model-domain coverage, but they should not be interpreted as complete substitutes for the composite AD score. The composite AD class also includes Morgan fingerprint similarity and descriptor-space $D_{5NN}$, which explains why some candidates with acceptable leverage can still be classified as low/out-of-domain.

## 3. Discussion

### 3.1. Value of the Matched Four-Isoform Ki Matrix

The 3200-compound matched matrix, in which every included molecule carries experimentally reported *K_i_* values against CA I, CA II, CA IX, and CA XII simultaneously, is the structural foundation that made isoform-selectivity modeling in this study possible, rather than merely one dataset choice among several. Most published CA inhibitor QSAR studies model a single isoform in isolation or assemble selectivity indices post hoc from disjoint single-target datasets collected under heterogeneous assay conditions [14,15]. Because every compound in the present matrix has a directly paired four-isoform activity profile, selectivity indices and Δ*pK_i_* values could be calculated as true intramolecular comparisons rather than as ratios between compounds tested in different laboratories, under different assay formats, against different isoform constructs. This distinction matters mechanistically: a compound’s own CA II *K_i_*, measured in the same source and often the same assay campaign as its CA IX *K_i_*, is a far more reliable estimate of a real selectivity margin than a ratio built from two independently sourced ChEMBL records. The matched-matrix design therefore enabled explicit separation of potency from selectivity throughout this study (Section 2.2)—a distinction essential given that many of the most potent CA inhibitors reported in the literature are not isoform-selective, and vice versa [5,11,13].

This design choice carries a corresponding limitation that should be stated plainly. Compounds with complete four-isoform profiling are, almost by definition, compounds that have already attracted sustained medicinal-chemistry interest—largely classical sulfonamide and sulfamate chemotypes explored across multiple CA isoforms in structure–activity campaigns [5,13]. Chemotypes profiled against only one or two isoforms, including some genuinely novel scaffolds, were necessarily excluded from the matched matrix and therefore could not contribute a training signal. The matched-matrix approach thus improves selectivity interpretability at the cost of underrepresenting the least-studied regions of CA inhibitor chemical space—a trade-off directly visible in Section 2.7′s finding that the most structurally novel prioritized candidates, particularly for CA I, also showed the weakest applicability-domain support (Section 2.8).

### 3.2. Predictive Performance, Validation Strategy, and Model Selection

The four isoform-specific stacked-ensemble models achieved held-out test-set R^2^ values of 0.727 (CA I), 0.719 (CA II), 0.652 (CA IX), and 0.607 (CA XII), with corresponding MAE/RMSE values of 0.500/0.709, 0.532/0.774, 0.493/0.666, and 0.482/0.654 *pK_i_* units (Section 2.4). These values indicate that the models captured substantial, chemically meaningful QSAR signal while still facing the expected difficulty of extrapolating across the structurally heterogeneous sulfonamide, sulfamate, coumarin-like, heteroaryl, and non-classical chemotypes that constitute the CA inhibitor literature [6,18]. The consistent ranking of stacked-ensemble performance above individual base learners (Section 2.4) indicates that nonlinear and ensemble methods were better suited to this chemistry than purely linear approaches, echoing conclusions from other CA-focused and general QSAR benchmarking studies [14,22,23].

Importantly, held-out cross-validation accuracy alone was not treated as sufficient grounds for model selection. The stacked ensemble was retained not because it was the single highest-scoring configuration on internal cross-validation, but because it offered the most favorable combination of held-out test accuracy, low prediction bias, external PubChem concordance (Section 2.6), interpretable descriptor-level structure (Section 2.5), and well-behaved applicability-domain characteristics (Section 2.8)—criteria that jointly determine whether a model is fit for prospective screening, not merely fit for a training partition [21,23]. This multi-criterion validation strategy is consistent with recent calls in the QSAR literature to weight external, prospective concordance more heavily than internal cross-validation metrics when models are intended to guide real screening decisions [23,36].

### 3.3. Isoform-Specific Chemical Information Learned by the QSAR Models

Descriptor importance and SAR triage of the final prioritized candidates (Section 2.3 and Section 2.7) indicate that the four models learned distinct, isoform-appropriate chemical signatures rather than a single generic CA-inhibition rule.

For CA I, the model favored compact, non-classical, or entrance-compatible motifs over canonical zinc-binding sulfonamides: none of the ten highest-priority novel CA I candidates carried a primary sulfonamide group (Section 2.7). This should not be read as the model having “discovered” a new CA I binding mode—no docking or crystallographic confirmation has yet been obtained—but rather as the model identifying a set of structurally distinct hypotheses that merit downstream mechanistic testing. This finding is consistent with CA I’s comparatively narrow, rigid active-site entrance, proposed to disfavor bulky or rigid zinc-binding architectures relative to CA II [13].

For CA II, the model strongly reflects classical primary-sulfonamide chemistry: all ten highest-priority CA II candidates carried this canonical zinc-binding group. This is chemically unsurprising given that CA II is the most extensively characterized CA isoform and the historical benchmark for sulfonamide-based inhibitor design [4,5]; the model’s convergence on this well-established chemotype is a reassuring internal consistency check rather than a novel finding in itself.

For CA IX, descriptor importance patterns (Section 2.5) point toward features associated with extended hydrophobic and aromatic substitution, consistent with CA IX’s wider active-site entrance and established entrance-region-exploiting design strategies [10,17]. However, this broader landscape-level signature should be distinguished from the specific final candidates prioritized in Section 2.7, all ten of which were themselves classical primary-sulfonamide scaffolds. Taken together, these two observations indicate that while the CA IX model has learned the entrance-region selectivity determinants documented in the broader literature, its highest-confidence novel predictions currently concentrate in the same classical chemical space as CA II, rather than exploiting non-classical entrance-directed chemistry—a distinction worth testing explicitly in future candidate generations.

For CA XII, the model captured a genuinely mixed pattern, combining classical sulfonamide/sulfamate features with larger heteroaryl and polar–hydrophobic architectures (Section 2.7): three of five final candidates were classical zinc-binders, one was a non-classical carboxylate, and one carried a non-canonical sulfonate-ester group requiring mechanistic caution (Section 2.7). This heterogeneity is consistent with CA XII’s comparatively open, solvent-exposed rim, which structurally tolerates a wider range of tail chemistries than CA I or CA II [10,12].

Collectively, the four models did not simply reproduce a single global CA-inhibition potency signal; they captured isoform-dependent chemical signatures broadly consistent with known differences among CA active-site rims and entrance regions, while also surfacing specific discrepancies—most notably for CA IX—between the general literature-derived landscape and the model’s own highest-confidence predictions.

### 3.4. Explainability Analysis Supports Chemically Plausible Isoform-Specific SAR

Descriptor importance and SHAP-based explainability analysis (Section 2.5) converged with established CA inhibitor structure–activity relationships rather than surfacing arbitrary statistical artifacts. Sulfur-, oxygen-, and nitrogen-containing atom-pair descriptors tracked known sulfonamide, sulfamate, sulfonyl, and amide zinc-binding motifs; CATS2D and SHED pharmacophore descriptors captured donor/acceptor and hydrophobic distributions relevant to tail–rim compatibility; and topological/eigenvalue descriptors reflected molecular size, branching, and aromaticity differences consistent with the isoform-specific rim architectures discussed in Section 2.2. The particular prominence of pharmacophore-geometry and heteroatom descriptors for CA II, contrasted with shape- and hydrophobicity-weighted descriptors for CA IX and CA XII, mirrors the classical-versus-entrance-directed distinction already established for these isoforms in the medicinal-chemistry literature [10,12,13].

This concordance should nonetheless be interpreted with an explicit epistemic caveat. Because the SHAP analysis in this study used mean absolute values to rank descriptor importance, it quantifies the magnitude of a descriptor’s contribution to model output, not the direction of that contribution nor a causal mechanism of binding. Descriptor-level explainability was therefore used throughout this study as a chemical-plausibility and prioritization tool—a way of checking that the model’s learned signal is chemically sensible—rather than as direct evidence of binding mechanism, consistent with recent cautions in the pharmaceutical XAI literature against over-interpreting feature-importance outputs as mechanistic proof [31,32,33,34].

### 3.5. PubChem-Based Prospective Screening and External Experimental Concordance

Four independent PubChem similarity campaigns, seeded from potent ChEMBL inhibitors and scored against all four isoform-specific QSAR models, extended model assessment beyond the internal ChEMBL training and test partitions (Section 2.6). Throughout this screening process, candidate ranking was based exclusively on predicted *K_i_*/*pK_i_* values and predicted selectivity indices. PubChem experimental records were used only after ranking to define novelty, prior evidence, and concordance status; they were not used to select, reorder, or calculate the final candidate priorities (Section 2.6 and Section 2.7).

This separation between prediction and evidence checking is important. Among the 4000 prioritized PubChem candidates, 847 compounds carried at least one direct human CA isoform-specific *K_i_* record, and 428 had complete four-isoform *K_i_* coverage across CA I, CA II, CA IX, and CA XII (Section 2.6). These records provided an external experimental concordance layer that internal cross-validation cannot provide, because the PubChem candidates were retrieved through analogue expansion and then evaluated after QSAR ranking rather than being part of the original model-training workflow. Same-target concordance between predicted and experimental *pK_i_* values was strong across the four isoforms, with Pearson correlations > 0.86, linear-fit R^2^ values > 0.74, and MAE values < 0.35 *pK_i_* units. This supports model transferability beyond internal test-set accuracy alone.

However, this assessment should be characterized precisely rather than described as unqualified prospective validation. PubChem bioassay records are heterogeneous in assay format, laboratory conditions, target annotation, and reporting conventions, and some concordant compounds are close analogues of known CA inhibitor chemotypes rather than fully independent chemical matter. Moreover, the 3572 candidates lacking complete four-isoform experimental *K_i_* coverage—the majority of the prioritized set—are best interpreted as novel or partially novel computational hypotheses rather than externally confirmed active compounds. Therefore, the PubChem analysis is most appropriately described as an external experimental concordance assessment supporting model transferability, not as definitive prospective experimental validation of the final prioritized candidates.

### 3.6. SAR, ADME, and Developability of the Prioritized Candidates

Predicted potency and selectivity alone were insufficient to identify chemically and pharmacologically viable candidates, motivating the structure- and ADME-based triage described in Section 2.7. Structure-based SAR review separated classical zinc-binding sulfonamide/sulfamate scaffolds from non-classical or mechanistically uncertain chemotypes. This review also identified two CA XII candidates bearing a sulfonyl chloride group rather than a stable sulfonamide or sulfamate zinc-binding moiety. Because sulfonyl chlorides are hydrolytically reactive electrophilic groups and are unlikely to persist as stable biological hit structures under aqueous or biological conditions, these compounds were excluded from the final candidate set rather than advanced on predicted-potency grounds alone. They may still serve as analogue-design templates after conversion to more stable sulfonamide, sulfamate, or related zinc-binding derivatives.

SwissADME profiling subsequently identified an independent class of developability liability not apparent from predicted potency or selectivity alone. Three CA XII candidates, CID 227581, CID 3917216, and CID 4993108, showed favorable predicted potency and selectivity during QSAR/SAR triage but returned very poor ADME scores of 0–7/100. These liabilities were driven by poor predicted gastrointestinal absorption, two Lipinski and two Veber violations, excessive polarity and/or flexibility, predicted P-glycoprotein substrate behavior, and low predicted bioavailability scores of approximately 0.17. These candidates were therefore deprioritized despite favorable ligand-based activity predictions, illustrating that QSAR/SAR ranking alone is insufficient to identify practical developability liabilities.

CID 4460361 illustrates why SAR, ADME, and AD filters must be interpreted jointly rather than treated as sequential pass/fail gates. This CA XII candidate showed a favorable ADME profile and strong applicability-domain support (Section 2.8), but it contains a non-canonical sulfonate ester/sulfonate-like motif rather than a validated primary sulfonamide or sulfamate zinc-binding group. Neither SwissADME profiling nor applicability-domain analysis can resolve this mechanistic uncertainty, because both operate primarily on ligand properties and similarity to learned chemical space rather than on explicit catalytic-site coordination geometry. Accordingly, CID 4460361 was retained as a prioritized CA XII candidate only with an explicit mechanistic caveat: its predicted activity should not be interpreted as evidence of canonical Zn^2+^ coordination until verified by zinc-aware docking, binding-mode analysis, and experimental inhibition testing.

Taken together, the developability triage prevented the virtual-screening workflow from collapsing into a potency-only ranking exercise. Instead, final prioritization integrated predicted potency, predicted selectivity, SAR plausibility, structural-alert review, SwissADME profile, and applicability-domain support, producing a chemically and pharmacologically more realistic candidate set for downstream validation.

The exclusion of CID 175661362 and CID 57773653 also provides useful guidance for analogue optimization. Both compounds were deprioritized because their sulfonyl chloride motif is inherently reactive and prone to hydrolysis or nonspecific reaction with biological nucleophiles, making them unsuitable as direct biological candidates regardless of predicted potency. However, their broader scaffold and tail features may remain useful as design templates. A reasonable optimization strategy would be to replace the sulfonyl chloride group with a more stable zinc-binding or polar recognition motif, such as a primary sulfonamide, sulfamate, sulfamide, sulfonylurea, sulfonate, or non-classical bioisostere, while preserving the substituent pattern predicted to favor CA XII recognition. The resulting analogues should then be re-evaluated using the same QSAR, SAR/ADME, and applicability-domain workflow, followed by zinc-aware docking, before biochemical testing is undertaken.

### 3.7. Applicability Domain as a Reliability Layer for Candidate Prioritization

Applicability-domain (AD) analysis (Section 2.8) provided a complementary reliability layer distinct from potency, selectivity, SAR plausibility, and ADME/developability. Its purpose was to determine whether each prediction was supported by proximity to the model’s learned chemical space or represented a more extrapolative, novelty-driven hypothesis. Across all four isoforms, held-out test-set prediction error increased from very-high AD to low/out-of-domain classes (Table 6), supporting the composite AD score as a useful reliability annotation rather than an arbitrary post hoc filter.

AD support varied substantially by isoform and therefore carried direct interpretive consequences. CA II and CA XII candidates showed the strongest overall AD support, consistent with their concentration in classical or well-precedented CA inhibitor chemical space. CA IX showed intermediate support, with two very-high-AD candidates and additional candidates requiring caution. In contrast, all five CA I candidates fell into the low/out-of-domain class, reinforcing the interpretation that they are exploratory, non-classical design hypotheses rather than high-confidence in-domain predictions. This distinction is important because novelty and reliability are not equivalent: structurally novel candidates may be scientifically interesting but require stronger downstream validation before potency or selectivity claims are made.

Critically, AD support is a statement about ligand similarity, descriptor-space proximity, and leverage relative to the training domain; it is not a statement about binding mode. CID 4460361 illustrates this distinction. Its strong AD support indicates that the compound lies within the learned CA XII ligand domain, but this does not confirm that its non-canonical sulfonate ester/sulfonate-like group can achieve genuine Zn^2+^ coordination. The same principle applies generally: AD can refine prediction confidence, but it cannot replace SAR review, zinc-aware docking, molecular dynamics, or experimental *K_i_* determination.

Integrating AD with the SAR and ADME triage described in Section 3.6 yields a tiered confidence framework for the twenty final candidates. Very-high-AD candidates with acceptable SAR and ADME profiles represent the most appropriate compounds for immediate zinc-aware docking and experimental prioritization. Moderate-AD candidates should be advanced with explicit caution and interpreted as lower-confidence but still plausible analogues. Low/out-of-domain candidates, particularly the CA I set, are best treated as novelty-oriented scaffolds requiring substantially more supporting evidence before specific activity or selectivity claims are made.

### 3.8. Therapeutic Implications of Isoform-Selective CA Inhibitor Prioritization

Isoform selectivity is not a secondary refinement in carbonic anhydrase inhibitor design, but a central therapeutic requirement. Because the catalytic Zn^2+^ site is highly conserved across human CA isoforms, potent inhibitors can easily display broad cross-isoform activity unless selectivity is deliberately engineered. In this context, CA II inhibition represents a major off-target liability for systemic CA inhibitors and has historically contributed to the tolerability limitations of non-selective sulfonamide-based CA therapeutics [4,5]. Therefore, reducing residual CA II activity while maintaining potency against disease-associated isoforms remains a key medicinal-chemistry objective.

This requirement is especially relevant for CA IX and CA XII. Both isoforms are associated with tumor pH regulation, extracellular acidification, hypoxia adaptation, invasion, metastasis, and treatment resistance across multiple solid tumor contexts [1,2,3,7,8,9]. Selective inhibition of CA IX and/or CA XII while sparing CA II is therefore a clinically motivated design goal rather than a simple potency-maximization exercise. The present workflow directly addresses this challenge by ranking candidates using multi-isoform predicted activity profiles, target-specific selectivity indices, SAR plausibility, ADME/developability filters, and applicability-domain support.

CA I selectivity has a different translational interpretation. Although CA I is less prominent as an oncology target than CA IX or CA XII, it remains relevant to erythrocytic and physiological CA inhibition and can contribute to off-target pharmacology. The CA I candidates identified here should therefore be framed cautiously. Their low applicability-domain support and enrichment in non-classical scaffolds make them useful exploratory design hypotheses, but not high-confidence pharmacological leads.

Within this framework, the twenty final candidates prioritized in this study should be interpreted as biochemical starting points for isoform-selective CA inhibitor development, not as therapeutic leads. None has yet been confirmed by zinc-aware docking, molecular dynamics, or direct experimental *K_i_* determination. Predicted selectivity, even when supported by internal validation, PubChem concordance, SAR/ADME triage, and applicability-domain analysis, is not equivalent to demonstrated pharmacological selectivity in a biological system. The translational value of the present work therefore lies in prioritizing chemically plausible and model-supported candidates for targeted structural and experimental validation.

### 3.9. Limitations

Several limitations should be considered when interpreting these results. First, the ChEMBL and PubChem bioactivity records underlying this study are heterogeneous with respect to assay format, experimental conditions, laboratory source, compound preparation, and reporting convention [35,37]. Although the workflow applied conservative filtering to retain human isoform-specific *K_i_* values where possible, residual experimental noise and inter-assay variability cannot be fully eliminated.

Second, the 3200-compound matched four-isoform matrix provides a strong basis for compound-level selectivity analysis, but it is not an unbiased representation of all possible CA inhibitor chemistry. Because compounds with complete *K_i_* profiles against CA I, CA II, CA IX, and CA XII are more likely to belong to well-studied CA inhibitor classes, the matched matrix may underrepresent genuinely novel or sparsely characterized chemotypes. This limitation is particularly relevant for interpreting non-classical candidates, including the CA I-prioritized compounds.

Third, QSAR models predict statistical relationships between molecular structure and measured activity. They do not directly predict binding modes, zinc-coordination geometry, protonation-state behavior, residence time, or protein conformational response. Therefore, predicted potency and selectivity should not be interpreted as structural proof of target engagement.

Fourth, descriptor-importance and SHAP-based explainability analyses support chemical plausibility but do not establish mechanistic causality. Mean absolute SHAP and feature-importance values indicate the magnitude of descriptor contribution to model predictions, but they do not prove that a given functional group forms a specific interaction in the active site.

Fifth, applicability-domain classification measures prediction reliability relative to the model’s training chemical space. A high AD score increases confidence that a prediction is made within a familiar ligand domain, but it does not guarantee experimental activity or confirm a binding mechanism. Conversely, low/out-of-domain compounds may remain chemically interesting, but they require stronger downstream evidence before activity or selectivity claims can be made.

Sixth, zinc-aware molecular docking and molecular dynamics simulations were intentionally excluded from the present study and reserved for dedicated follow-up work. As a result, no candidate in the present manuscript has structural confirmation of its binding orientation, zinc-binding mode, or active-site interaction pattern. This limitation is especially important for candidates with non-canonical zinc-binding motifs, such as CID 4460361.

Finally, none of the twenty final candidates has yet been experimentally tested against the four CA isoforms under identical assay conditions. All reported potency, selectivity, SAR, ADME, and applicability-domain conclusions should therefore be interpreted as computational prioritization outputs. The present study is best viewed as a ligand-based screening and hypothesis-generation workflow, not as definitive biochemical validation of the proposed candidates.

A further limitation is the absence of direct experimental and structural validation for the final candidates. The present workflow combines ligand-based QSAR prediction, PubChem concordance, SAR/ADME triage, and applicability-domain analysis; therefore, it can prioritize chemically plausible candidates but cannot confirm biochemical activity, true isoform selectivity, Zn^2+^ coordination geometry, or binding orientation. This distinction is particularly important for candidates carrying non-canonical zinc-binding motifs or classified as low/out-of-domain, for which predicted potency and selectivity are least supported by structural or mechanistic evidence. Accordingly, the reported *K_i_*, *pK_i_*, SI, and Δ*pK_i_* values for the final PubChem candidates should be interpreted as model-derived prioritization metrics rather than experimentally validated inhibition constants. Definitive confidence in these candidates will require zinc-aware docking and molecular dynamics to assess mechanistic plausibility, followed by biochemical profiling against CA I, CA II, CA IX, and CA XII under identical assay conditions and, where feasible, structural characterization.

### 3.10. Future Work and Experimental Validation

The logical next stage of this work is structural and experimental confirmation of the twenty prioritized candidates. The immediate priority is zinc-aware molecular docking against validated CA I, CA II, CA IX, and CA XII structures. Docking should be preceded by careful receptor preparation, co-crystal ligand redocking, and benchmarking against known CA inhibitors to confirm that the selected docking protocol can reproduce experimentally observed binding modes. Ligand protonation state, sulfonamide/sulfamate ionization, zinc-coordination geometry, and active-site water interactions should be treated explicitly. General docking engines and workflows such as SwissDock and AutoDock Vina 1.2.0 provide useful platforms for this stage, but their application to CA inhibitors requires target-specific validation because of the catalytic Zn^2+^ center and the importance of zinc-binding-group geometry [44,45].

This step is particularly important for CID 4460361 and any other candidate carrying a non-canonical zinc-binding motif. For such compounds, docking should not be used merely to generate a plausible pose, but to test whether the proposed zinc-binding group can adopt a chemically reasonable coordination geometry within the CA active site. Candidates that fail this mechanistic check should be redesigned or deprioritized, even if their ligand-based QSAR, ADME, and applicability-domain profiles are favorable.

Future docking and molecular-dynamics studies should also explicitly account for active-site water molecules. In carbonic anhydrases, conserved and exchangeable waters can influence Zn^2+^ coordination geometry, hydrogen-bond networks, proton-transfer architecture, ligand orientation, and rim/entrance stabilization. Because the present ligand-based workflow does not model protein-bound water, it cannot evaluate water-mediated binding stability or water-dependent isoform selectivity. This limitation is particularly important for candidates carrying non-canonical zinc-binding motifs and for ligands whose predicted selectivity may depend on subtle entrance-region or tail/rim interactions that cannot be resolved by a static, water-free model. Follow-up structural modeling should therefore compare docking and MD protocols under retained, displaced, and exchangeable active-site water conditions, benchmarking each setting against co-crystallized CA inhibitors to determine which treatment best reproduces known binding geometries before application to the prioritized candidates.

Molecular dynamics simulations should then be applied to the most promising docking-supported candidates, particularly the strongest CA II, CA IX, and CA XII candidates identified by the integrated QSAR/SAR/ADME/AD framework. MD simulations would help evaluate binding-pose stability, active-site water networks, ligand flexibility, tail–rim interactions, and isoform-specific accommodation beyond static docking snapshots. Similar integrated virtual-screening, docking, and molecular-dynamics workflows have previously been applied to human CA II inhibitor discovery, supporting the use of docking and dynamic stability assessment as a logical follow-up to ligand-based QSAR prioritization [46].

Ultimately, direct experimental validation is essential. The highest-priority candidates should be tested in vitro against CA I, CA II, CA IX, and CA XII under identical assay conditions to confirm both potency and selectivity. This would extend the matched-matrix logic used in the present study to the newly prioritized PubChem-derived candidates. Experimental $K_{i}$ profiling across all four isoforms is especially important because single-target activity alone cannot establish isoform selectivity.

In parallel, synthesis or procurement feasibility should be assessed for each prioritized compound. High-AD, developability-clean candidates should be prioritized first for docking and biochemical testing where available, whereas lower-AD or mechanistically uncertain candidates should be used primarily as templates for analogue design. This combined strategy would translate the present computational workflow into a focused medicinal-chemistry program for discovering and validating isoform-selective CA inhibitors.

## 4. Materials and Methods

### 4.1. Data Acquisition and Bioactivity Curation

Bioactivity data were retrieved from ChEMBL version 34 [37] for four human carbonic anhydrase (CA) isoforms selected to represent both major cytosolic off-targets and tumor-associated therapeutic targets: CA I, CA II, CA IX, and CA XII. The corresponding ChEMBL target identifiers were CHEMBL261 for CA I, CHEMBL205 for CA II, CHEMBL3594 for CA IX, and CHEMBL3242 for CA XII. Each target was curated as a single-protein Homo sapiens target.

For each isoform, experimentally reported inhibition constants were extracted and standardized as $K_{i}$ values in nanomolar units. Only records carrying a single, numeric, positive $K_{i}$ value suitable for continuous regression modeling were retained. Entries reporting qualitative activity annotations, inequality-bound values such as $K_{i}>10,000$ nM, or non-$K_{i}$ endpoints, including $IC_{50}$, $K_{d}$, percent inhibition, or activity calls, were excluded at this stage. After bioactivity standardization and compound-level curation, the isoform-specific datasets contained 7204 CA I inhibitors, 7698 CA II inhibitors, 5631 CA IX inhibitors, and 4416 CA XII inhibitors. These four full isoform-specific datasets were used for the development, optimization, and evaluation of the corresponding QSAR models, as described in Section 4.7.

To place all bioactivity values on a comparable logarithmic regression scale, $K_{i}$ values were converted to $pK_{i}$ according to:

$$pK_{i}=9-{log}_{10}(K_{i}[nM])$$

where $K_{i}[nM]$ is the experimentally reported inhibition constant in nanomolar units. This transformation reduces the dominance of extreme nanomolar or micromolar values, improves suitability for continuous regression modeling, and allows predictive performance to be interpreted consistently across all four CA isoforms.

The CA I, CA II, CA IX, and CA XII ChEMBL datasets described in this section served as the primary model-development datasets for isoform-specific $pK_{i}$ prediction. The matched four-isoform activity matrix used for compound-level selectivity analysis was constructed separately and is described methodologically in Section 4.2, with its selectivity patterns reported in Section 2.2.

### 4.2. Construction of the Matched Four-Isoform Ki Matrix

In addition to the full isoform-specific ChEMBL datasets used for QSAR model development (Section 4.1), a separate matched four-isoform activity matrix was constructed to support direct compound-level selectivity analysis. This matrix was generated by retaining only compounds for which complete experimental *K_i_* values were available against all four modeled human CA isoforms—CA I, CA II, CA IX, and CA XII—simultaneously. After matching compounds across the four curated isoform-specific datasets, this procedure yielded 3200 unique molecules with complete four-isoform activity profiles.

The matched matrix was not used as a replacement for the larger isoform-specific QSAR training datasets. Instead, it served as a dedicated selectivity-analysis layer: because every compound in this subset carried paired experimental activity values for all four isoforms, potency could be compared directly across target and off-target isoforms within the same molecule, avoiding the ambiguity that arises when selectivity is inferred from unrelated single-target datasets collected under different assay conditions [14,15].

For each compound in the matched matrix, the standardized *K_i_* and corresponding *pK_i_* values were retained for all four isoforms. Target-specific selectivity indices were then calculated for each isoform. For a given target isoform *t*, the selectivity index was defined as:

$$SI_{t}=\frac{min(K_{i}\;\mathrm{of}\;\mathrm{off-target}\;\mathrm{isoforms})}{K_{i}\;\mathrm{of}\;\mathrm{target}\;\mathrm{isoform}}$$

where the off-target isoforms are the remaining three CA isoforms represented in the matched matrix. Higher $SI_{t}$ values indicate stronger experimental preference for the target isoform relative to its most sensitive off-target isoform. The isoform corresponding to the minimum off-target *K_i_* was recorded as the limiting off-target for that compound.

In parallel, logarithmic selectivity differences were calculated from paired *pK_i_* values. For a given target isoform *t*, the global target-versus-off-target selectivity difference was defined as:

$$\Delta pK_{i}(t)=pK_{i}(t)-max[pK_{i}(\mathrm{off-target}\;\mathrm{isoforms})]$$

Positive Δ*pK_i_* values indicate preferential inhibition of the target isoform, whereas negative values indicate that at least one off-target isoform was more strongly inhibited than the intended target. CA IX-centered Δ*pK_i_* endpoints were additionally calculated to support tumor-associated CA IX selectivity interpretation:

$$\Delta pK_{i}(\mathrm{CA}\;\mathrm{IX}-\mathrm{CA}\;I)=pK_{i}(\mathrm{CA}\;\mathrm{IX})-pK_{i}(\mathrm{CA}\;I)$$

$$\Delta pK_{i}(\mathrm{CA}\;\mathrm{IX}-\mathrm{CA}\;\mathrm{II})=pK_{i}(\mathrm{CA}\;\mathrm{IX})-pK_{i}(\mathrm{CA}\;\mathrm{II})$$

$$\Delta pK_{i}(\mathrm{CA}\;\mathrm{IX}-\mathrm{CA}\;\mathrm{XII})=pK_{i}(\mathrm{CA}\;\mathrm{IX})-pK_{i}(\mathrm{CA}\;\mathrm{XII})$$

$$\Delta pK_{i}(\mathrm{CA}\;\mathrm{IX}-max\;\mathrm{off-target})=pK_{i}(\mathrm{CA}\;\mathrm{IX})-max[pK_{i}(\mathrm{CA}\;I),pK_{i}(\mathrm{CA}\;\mathrm{II}),pK_{i}(\mathrm{CA}\;\mathrm{XII})]$$

Each compound was also assigned a most-selective isoform based on its highest target-specific selectivity index. These derived variables enabled ranking of experimentally characterized CA I-, CA II-, CA IX-, and CA XII-selective inhibitors and supported the structural selectivity interpretation summarized in Section 2.2.

The complete matched matrix—including ChEMBL identifiers, molecular structures, four-isoform *K_i_* and *pK_i_* values, target-specific selectivity indices, limiting off-targets, Δ*pK_i_* endpoints, and most-selective isoform assignments—is provided as Supplementary Table S1. The top-ranked experimentally selective compounds derived from this matrix are summarized separately in Supplementary Table S2.

### 4.3. External Chemical-Space Expansion via PubChem Similarity Search

To expand the modeled chemical space beyond the curated ChEMBL compounds, ligand-based virtual screening was performed using PubChem similarity searching. For each CA isoform, potent ChEMBL inhibitors with experimentally reported *K_i_* < 10 nM were used as seed compounds. These seeds were submitted independently for 90% two-dimensional structural similarity searching against the PubChem compound database [35,36], generating isoform-specific analogue pools for CA I, CA II, CA IX, and CA XII.

Retrieved PubChem analogues were standardized, merged, and deduplicated at the compound-structure level before model scoring. Each unique PubChem candidate was then evaluated across all four isoform-specific QSAR models, irrespective of the isoform from which it was originally retrieved. Thus, every candidate received predicted $K_{i}$ and $pK_{i}$ values for CA I, CA II, CA IX, and CA XII, enabling target-specific selectivity indices and $\Delta pK_{i}$ values to be calculated consistently across the four modeled isoforms.

The PubChem similarity expansion was used to generate external candidate pools for prospective ligand-based prioritization. Experimental PubChem bioactivity records were not used during candidate ranking or QSAR prediction. Instead, they were retrieved and filtered only after prediction to support the independent experimental-evidence and concordance analysis described in Section 4.9. This separation ensured that PubChem-derived experimental information did not influence virtual-screening rank order or predicted selectivity estimates.

### 4.4. Descriptor Generation Using alvaDesc

Molecular structures were represented as canonical SMILES strings and encoded using alvaDesc version 3.0.8 [28]. For each compound, an initial panel of 4185 two-dimensional molecular descriptors was calculated. These descriptors spanned 22 of the 30 alvaDesc descriptor families, specifically those computable directly from the two-dimensional molecular graph. The retained descriptor classes included constitutional, topological, atom-centered, fragment-based, autocorrelation, eigenvalue-based, charge-related, surface-area, and pharmacophore-type descriptors. Descriptor families requiring three-dimensional optimized conformers, including geometrical, 3D-autocorrelation, WHIM, GETAWAY, and 3D-MoRSE descriptors, were excluded by design.

Two-dimensional descriptors were selected because they provide a computationally efficient and conformer-independent representation of molecular structure, making them suitable for large-scale ligand-based QSAR modeling and PubChem virtual screening. In contrast to three-dimensional descriptors, 2D descriptors do not require conformer generation, geometry optimization, or binding-pose assumptions. This reduced the uncertainty associated with conformational sampling [26] and was appropriate for the present workflow, which involved thousands of ChEMBL inhibitors and large PubChem-derived analogue pools.

Descriptor calculation was performed independently for the curated CA I, CA II, CA IX, and CA XII datasets described in Section 4.1. The same descriptor-generation procedure was subsequently applied to PubChem candidate structures described in Section 4.3 to ensure consistency between model development and virtual screening. Descriptor names and definitions followed alvaDesc nomenclature. The final selected isoform-specific descriptor subsets, obtained after preprocessing, filtering, and Random Forest importance ranking, are provided in Supplementary Table S3.

### 4.5. Leakage-Controlled Preprocessing and Feature Filtering

Descriptor preprocessing was performed independently for each isoform-specific dataset to ensure that the CA I, CA II, CA IX, and CA XII models were developed using target-specific chemical information. To minimize information leakage, each curated dataset was first divided into a training subset and a held-out test subset before model-dependent preprocessing and feature selection were finalized. The training subset was used to estimate all preprocessing parameters, and the same fitted transformations were then applied unchanged to the held-out test subset and to PubChem-derived candidate structures.

Descriptors with missing or non-finite values were handled using training-set statistics: missing values were imputed with the corresponding training-set median for each descriptor, and descriptors that remained undefined, constant, or numerically unstable after imputation were removed. This ensured that the same descriptor space could be applied consistently across training compounds, held-out test compounds, and external PubChem candidates.

To remove non-informative variables, descriptors with near-zero variance were excluded using a variance threshold of <0.01, calculated from the training subset only, eliminating descriptors that carried little discriminatory information within the chemical space of the corresponding isoform. Remaining descriptors were then standardized using Z-score normalization:

$$z=\frac{x-\mu_{\mathrm{train}}}{\sigma_{\mathrm{train}}}$$

where $\mu_{\mathrm{train}}$ and $\sigma_{\mathrm{train}}$ are the descriptor mean and standard deviation calculated from the training subset; the same training-derived scaling parameters were subsequently applied to the held-out test subset and PubChem candidates.

To reduce redundancy and multicollinearity, highly correlated descriptors were removed using a Pearson correlation threshold of |r| > 0.95, calculated within the training subset; for each highly correlated descriptor pair or cluster, redundant descriptors were removed while retaining a representative descriptor for subsequent model development. This reduced the dimensionality of the original 4185-descriptor matrix and improved numerical stability, interpretability, and model robustness.

The resulting leakage-controlled descriptor matrices were then passed to the Random Forest importance-ranking step described in Section 4.6. This preprocessing strategy ensured that held-out test performance and PubChem candidate predictions were evaluated using transformations learned only from the training chemical space, reducing the risk of optimistic performance estimates.

### 4.6. Feature Selection by Random Forest Importance Ranking

Following leakage-controlled preprocessing and unsupervised feature filtering (Section 4.5), a supervised feature-selection step was performed using Random Forest (RF) importance ranking. This step was applied independently for each isoform-specific modeling task to identify the descriptors most informative for predicting *pK_i_* values against CA I, CA II, CA IX, and CA XII.

For each isoform, an H2O Random Forest regression model was trained in KNIME [38,39] using only the corresponding training subset, configured with 500 trees and a maximum tree depth of 50. Variable-importance measures generated by the trained RF model were extracted and used to rank the remaining descriptors. This supervised ranking was performed strictly within the training workflow; the held-out test subset was not used for descriptor selection, model tuning, or importance ranking. The selected descriptor list was subsequently applied unchanged to the held-out test subset and to PubChem-derived candidate structures.

For each CA isoform, the top-100 RF-ranked descriptors were retained as the final isoform-specific descriptor panel, subsequently used for QSAR model development, model interpretation, and descriptor-space applicability-domain analysis. Because feature selection was conducted separately for each isoform, the final descriptor sets were allowed to differ across CA I, CA II, CA IX, and CA XII. This isoform-specific design was intentional, reflecting the expectation that each CA isoform may depend on distinct combinations of molecular topology, heteroatom patterning, polarity, sulfur-containing fragments, pharmacophore spacing, and shape-related descriptors.

This RF-based dimensionality-reduction step served three purposes: reducing the descriptor space from the initial 4185 alvaDesc descriptors to a compact, model-relevant set of 100 descriptors per isoform; reducing the risk of overfitting associated with high-dimensional QSAR modeling; and improving downstream interpretability [41] by focusing subsequent modeling and applicability-domain analysis on descriptor families most strongly associated with isoform-specific CA inhibition.

The RF importance ranking described here was used as a feature-selection procedure, not as the final explainability analysis. Global and local model-interpretability analyses were performed separately using permutation feature importance and SHAP analysis, as described in Section 4.8; this distinction prevents the RF feature-selection step from being overinterpreted as mechanistic evidence. The complete lists of final selected descriptors and their RF importance rankings are provided in Supplementary Table S3.

### 4.7. QSAR Model Development, Optimization, and Evaluation

Four isoform-specific QSAR regression models were developed to predict *pK_i_* values for CA I, CA II, CA IX, and CA XII. Model development was performed independently for each isoform using the final 100 descriptors selected for that isoform (Section 4.6). All machine-learning workflows were implemented in the KNIME Analytics Platform using the H2O.ai machine-learning framework [38,39], enabling reproducible model training, cross-validation, hyperparameter optimization, and prediction of external PubChem-derived candidates.

For each isoform, four complementary regression algorithms were evaluated as base learners: Random Forest (RF), Generalized Linear Model (GLM), Gradient Boosting Machine (GBM), and Artificial Neural Network (ANN). These algorithms were selected to cover linear, bagging-based, boosting-based, and neural nonlinear learning strategies, allowing the workflow to compare simpler interpretable models with more flexible nonlinear models capable of capturing complex descriptor–activity relationships.

Model optimization was performed within the training subset using five-fold cross-validation. H2O AutoML tuned model hyperparameters by random search, with mean residual deviance as the primary optimization criterion. For RF and GBM models, tuned hyperparameters included tree number, tree depth, sampling parameters, and split-related controls; for GLM models, regularization parameters controlling the balance between L1 and L2 penalties were optimized; for ANN models, network architecture and learning parameters were optimized automatically within the H2O framework.

To exploit the complementary predictive strengths of the individual learners, a stacked ensemble was constructed for each isoform. During five-fold cross-validation, out-of-fold predictions from the base learners were generated and used to train the ensemble meta-learner—reducing information leakage, since the meta-learner was trained only on predictions for compounds not used to fit the corresponding base-learner fold. The stacked ensemble thereby combined the linear contribution of GLM, the bagging-based robustness of RF, the sequential error-correction capacity of GBM, and the nonlinear pattern-recognition capacity of ANN [22,23].

Final model performance was evaluated using an independent held-out test subset (20% of each isoform-specific dataset). Primary evaluation metrics were the coefficient of determination (R^2^), mean absolute error (MAE), root mean squared error (RMSE), mean squared error (MSE), Pearson correlation coefficient, prediction bias, and residual deviance.

The stacked-ensemble models were selected for downstream PubChem screening not on held-out test accuracy alone, but on the most favorable combination of held-out test performance, low prediction bias, external PubChem experimental concordance (Section 4.9), interpretable descriptor-level structure (Section 4.8), and well-behaved applicability-domain characteristics (Section 4.11)—criteria that jointly determine fitness for prospective screening rather than fitness for a single training partition (Section 3.2).

This modeling strategy provided four independently optimized isoform-specific predictors. Each PubChem candidate was subsequently scored by all four models, generating predicted *pK_i_* and back-transformed *K_i_* values for CA I, CA II, CA IX, and CA XII. These predictions formed the basis for downstream selectivity-index calculation, PubChem concordance analysis, SAR/ADME triage, and applicability-domain-guided prioritization.

### 4.8. Model Interpretability: Permutation Feature Importance and SHAP Analysis

To support chemical interpretation of the isoform-specific QSAR models, two complementary explainable artificial intelligence (XAI) approaches were applied: permutation feature importance (PFI) and SHapley Additive exPlanations (SHAP) [31,32,33,34]. PFI evaluated global descriptor relevance at the model level, while SHAP analysis examined descriptor contributions to selected candidate predictions. Together, these analyses assessed whether the models relied on chemically interpretable descriptor patterns rather than purely statistical correlations.

PFI was calculated for each isoform-specific stacked-ensemble model (Section 4.7) by measuring the reduction in predictive performance following random permutation of individual descriptor values; descriptors whose permutation produced larger performance decreases were considered more influential. PFI was calculated separately for the training and held-out test subsets to distinguish descriptors important for fitted model behavior from those retaining importance under test-set generalization—a distinction that matters because training-set importance alone may reflect overfitting, whereas descriptors remaining important in the test subset provide stronger evidence of generalizable structure–activity signal.

SHAP analysis then provided local and compound-set-level interpretation of model predictions, estimating the contribution of each descriptor to the deviation of an individual prediction from the model’s average output. SHAP analysis was applied to prioritized candidate sets to identify descriptors contributing most strongly to predicted activity and to relate these descriptors to recognizable chemical features, including sulfur-containing motifs, hydrogen-bonding capacity, pharmacophore spacing, molecular shape, polarity, and aromatic or heteroatom-rich scaffold patterns.

Because both PFI and SHAP can be affected by descriptor correlation, redundancy, and training-domain structure, the resulting explanations were interpreted cautiously. PFI was treated as a global relevance measure rather than proof of causal molecular interaction. Similarly, mean absolute SHAP values were used to quantify the magnitude of descriptor contribution, not its direction or mechanistic origin [24,40]. XAI outputs were accordingly used as chemical-plausibility and prioritization evidence—complementary to SAR review, PubChem concordance, ADME profiling, and applicability-domain analysis (Section 4.9, Section 4.10 and Section 4.11)—rather than as direct proof of binding mechanism or isoform selectivity.

### 4.9. PubChem Experimental-Evidence Filtering and Concordance Analysis

After QSAR scoring and selectivity-based prioritization of PubChem-derived candidates (Section 4.3), available PubChem bioactivity records were retrieved [35] to evaluate whether any prioritized compounds had independent experimental evidence against the modeled human CA isoforms. This evidence-retrieval step was performed after virtual-screening ranking, ensuring that PubChem experimental records did not influence predicted potency, predicted selectivity index, or candidate prioritization at any stage.

PubChem assay records were filtered conservatively to retain only direct, human isoform-specific *K_i_* measurements for the four modeled targets: CA I, CA II, CA IX, and CA XII. Records were excluded from concordance analysis if they corresponded to unrelated targets, non-human enzymes, non-modeled CA isoforms, non-*K_i_* endpoints (IC_50_, *K_d_*, percent inhibition, cytotoxicity assays, selectivity-ratio rows), or qualitative activity annotations. This filtering was necessary because PubChem assay summaries are heterogeneous [36,37] and may contain multiple endpoint types, target annotations, and assay formats not directly comparable to the ChEMBL *K_i_*-based QSAR models.

Retained PubChem *K_i_* values were standardized to *pK_i_* using the same unit-specific transformations applied during model development. Values reported in nM were converted according to:

$$pK_{i}=9-{log}_{10}(K_{i}\;[\mathrm{nM}])$$

whereas values reported in µM were converted as:

$$pK_{i}=6-{log}_{10}(K_{i}\;[\mu M])$$

For concordance analysis, experimental PubChem *pK_i_* values were compared only with the QSAR prediction for the same compound and the same CA isoform—a CA II experimental *K_i_* value was compared only with the CA II predicted *pK_i_*, never with predictions for CA I, CA IX, or CA XII. This same-target comparison rule prevented unrelated or cross-isoform records from inflating apparent validation performance.

Each prioritized PubChem compound was assigned an experimental-evidence status based on the filtered records: compounds with direct *K_i_* evidence for all four modeled isoforms were classified as having complete four-isoform experimental coverage; compounds with direct *K_i_* evidence for one to three isoforms were classified as partially covered; and compounds lacking direct human CA *K_i_* evidence for any of the four isoforms were classified as having no direct modeled-isoform *K_i_* evidence. This classification distinguished experimentally supported compounds from target-novel or partially novel candidates.

Exact structure matches between PubChem candidates and ChEMBL compounds used in model development were identified and annotated separately. Such matches provide useful recovery and consistency information but were not treated as fully independent prospective validation, since the same chemical structures may already be represented in the ChEMBL-derived modeling space. PubChem-based evaluation was therefore interpreted as an external experimental concordance assessment, not as unqualified prospective experimental validation.

Model concordance was assessed by comparing predicted *pK_i_* values with retained PubChem experimental *pK_i_* values for matched compound–isoform pairs, summarized using Pearson correlation coefficient, linear-fit R^2^, mean absolute error, and root mean squared error, calculated separately for CA I, CA II, CA IX, and CA XII to evaluate isoform-specific transferability of the QSAR predictions. The resulting concordance tables and evidence classifications are provided in Supplementary Table S4, while the candidate novelty classifications derived from this filtering process are provided in Supplementary Table S5.

### 4.10. SAR Triage and SwissADME-Based Developability Assessment

To support early-stage developability assessment prior to structural and experimental follow-up, target-novel and partially novel PubChem candidates (Section 4.9) were subjected to structure–activity relationship (SAR) triage and SwissADME-based pharmacokinetic profiling [47,48]. For each isoform, the ten highest-priority candidates selected after QSAR-based potency and selectivity ranking were evaluated, yielding 40 candidates across CA I, CA II, CA IX, and CA XII.

SAR triage was performed using RDKit-assisted substructure annotation [49] followed by medicinal-chemistry review. Candidate structures were screened for classical carbonic anhydrase zinc-binding motifs—primary sulfonamides, sulfamates, and related sulfonamide-like groups—as well as non-classical or mechanistically uncertain motifs, including carboxylates, thiourea/thioamide groups, hydrazones, heteroaryl amides, sulfonate ester/sulfonate-like groups, and sulfonyl chlorides. This distinguished candidates with plausible classical Zn^2+^-anchored inhibition profiles from those requiring mechanistic caution; reactive or chemically unstable motifs were flagged before final prioritization. Sulfonyl chloride-bearing candidates, in particular, were treated as reactive-liability structures and retained only as analogue-design templates rather than final biological candidates.

SwissADME profiling [47] was then used to evaluate the physicochemical and pharmacokinetic properties of the SAR-triaged candidates. Canonical SMILES strings were submitted to calculate molecular weight, topological polar surface area, hydrogen-bond donors and acceptors, rotatable bonds, consensus logP, predicted gastrointestinal absorption, blood–brain barrier permeability, P-glycoprotein substrate propensity, drug-likeness rule compliance, PAINS alerts, Brenk alerts, bioavailability score, and synthetic accessibility. CYP inhibition fields were returned as “n/d” and were therefore not used in scoring, ranking, or prioritization. CYP inhibition alerts were not used in the final scoring because this output was no longer available from SwissADME after the May 2026 platform modification.

These descriptors identified candidates with unfavorable polarity, excessive molecular size or flexibility, poor predicted absorption, potential transporter or metabolism liabilities, structural-alert burden, or reduced medicinal-chemistry tractability.

SwissADME outputs were integrated into a heuristic 0–100 developability score to support candidate ranking. This score was not treated as an absolute pharmacokinetic prediction, but as a practical triage metric combining drug-likeness, absorption-related properties, structural-alert burden, bioavailability, and synthetic accessibility. Critically, ADME scoring did not override SAR-based chemical alerts: candidates containing reactive or mechanistically problematic motifs were flagged even when their SwissADME profiles appeared acceptable, while candidates with favorable predicted potency and selectivity were deprioritized when SwissADME indicated severe developability liabilities—poor gastrointestinal absorption, multiple Lipinski or Veber violations, high polarity or flexibility, P-glycoprotein substrate prediction, or low predicted bioavailability.

Final prioritization combined QSAR-predicted potency, predicted selectivity index, SAR plausibility, structural-alert review, SwissADME profile, and medicinal-chemistry developability, selecting five final candidates per isoform for downstream applicability-domain confirmation and future Zn^2+^-aware docking. SwissADME-based properties and final developability rankings are provided in Supplementary Table S6B.

### 4.11. Applicability-Domain Methodology

Applicability-domain (AD) analysis was performed independently for each isoform-specific QSAR model to estimate whether held-out test compounds and final prioritized PubChem candidates were located within the chemical and descriptor space represented by the corresponding training set. This step was included because QSAR predictions are most reliable when applied to compounds sufficiently similar to the training domain, and recent QSAR guidance emphasizes explicit reliability and domain assessment as part of model interpretation and prediction reporting [23,50].

Three complementary AD criteria were used: Morgan fingerprint similarity [29,49], descriptor-space distance, and leverage. Morgan fingerprints were calculated with radius 2 and 2048 bits, and each compound was characterized by its maximum Tanimoto similarity and mean five-nearest-neighbor Tanimoto similarity relative to the corresponding isoform training set. Descriptor-space proximity was quantified as $D_{5NN}$, the mean standardized Euclidean distance to the five nearest training compounds using the final 100 selected descriptors (Section 4.6). Leverage was calculated as:

$$h_{i}=x_{i}^{T}(X^{T}X{)}^{-1}x_{i}$$

with warning threshold:

$$h^{*}=\frac{3(p+1)}{n}$$

where $x_{i}$ is the descriptor vector of compound *i*, X is the training descriptor matrix, *p* is the number of selected descriptors, and *n* is the number of training compounds.

For each isoform, AD thresholds were calibrated using the held-out test subset by relating AD metrics to absolute prediction error:

$$AE=|pK_{i,\mathrm{predicted}}-pK_{i,\mathrm{experimental}}|$$

Low-error test compounds were defined as those with AE ≤ 0.50 *pK_i_*, and acceptable-error compounds as those with AE ≤ 1.00 *pK_i_*. The high-confidence fingerprint threshold was defined as the median maximum Tanimoto similarity among low-error compounds, and the moderate-confidence threshold as the 5th percentile among acceptable-error compounds. Descriptor-distance thresholds were derived analogously using $D_{5NN}$, with lower distances indicating stronger domain support. Leverage was interpreted relative to the isoform-specific $h^{*}$ threshold.

The normalized fingerprint, descriptor-distance, and leverage components were combined into a composite AD score:

AD Score=0.45×Fingerprint Score+0.35×Descriptor Distance Score+0.20×Leverage Score

The composite AD weights were selected as a transparent heuristic rather than as literature-fixed constants. Fingerprint similarity was assigned the largest weight because scaffold and substructure proximity to the training set is the most direct indicator of local chemical-space support in ligand-based virtual screening. Descriptor-space distance was assigned the second-largest weight because the QSAR models were trained on a specific set of selected molecular descriptors and therefore require proximity to the training compounds within the same standardized descriptor space. Leverage was assigned a lower, non-zero weight because it identifies statistical extrapolation within the descriptor matrix but, when used alone, can miss substructural novelty that fingerprint similarity captures directly. The class thresholds were calibrated empirically against the held-out test subsets by relating each AD metric to absolute prediction error and were therefore applied as confidence annotations rather than universal exclusion cutoffs.

Compounds were classified as very high AD (AD Score ≥ 0.80), high AD (0.65 ≤ AD Score < 0.80), moderate AD (0.45 ≤ AD Score < 0.65), or low/out-of-domain (AD Score < 0.45). These classes were used as confidence annotations rather than strict exclusion rules—low-AD candidates were retained but flagged as extrapolative hypotheses requiring stronger downstream validation (Section 2.8 and Section 3.7).

Williams plots were generated for each isoform by plotting leverage against standardized residuals for the held-out test subset, with final candidates overlaid according to their computed leverage. Because prioritized candidates lack experimental *pK_i_* values, they were plotted at standardized residual = 0 to indicate leverage position only, not prediction accuracy; candidate AD classes were assigned from the full composite AD score described above, never from Williams-plot leverage position alone.

### 4.12. Software, Computational Environment, and Data Availability

All cheminformatics, machine-learning, model-interpretability, and data-processing steps were performed using a reproducible workflow integrating KNIME Analytics Platform version 5.8.2 LTS (KNIME AG, Zurich, Switzerland), H2O.ai AutoML/H2O-3 through the KNIME H2O Machine Learning Integration version 5.6.0.v202507151410 (H2O.ai, Mountain View, CA, USA), alvaDesc version 3.0.8 (Alvascience, Lecco, Italy), RDKit Binaries and Chemistry Type Definitions KNIME extension version 5.2.1.v202507231837 (RDKit open-source cheminformatics toolkit), PubChem (National Center for Biotechnology Information, National Library of Medicine, National Institutes of Health, Bethesda, MD, USA), ChEMBL version 34 (European Molecular Biology Laboratory–European Bioinformatics Institute, Hinxton, UK), SwissADME (Molecular Modelling Group, SIB Swiss Institute of Bioinformatics and University of Lausanne, Lausanne, Switzerland) [28,35,37,38,39,47,49], and spreadsheet-based supplementary outputs. No chemicals, reagents, devices, instruments, commercial cell lines, samples, or experimental materials were used because the study was fully computational.

Molecular descriptors were calculated using alvaDesc. Workflow construction, preprocessing, feature filtering, model training, prediction, model interpretation, and result integration were performed in KNIME Analytics Platform. Machine-learning model development used the H2O.ai AutoML framework through the KNIME H2O Machine Learning Integration. The evaluated regression model classes included Random Forest, Generalized Linear Model, Gradient Boosting Machine, Artificial Neural Network, and stacked-ensemble models.

RDKit functionality was accessed through the RDKit Binaries and Chemistry Type Definitions KNIME extension [49], and used for molecular standardization, canonical SMILES handling, Morgan fingerprint calculation, Tanimoto similarity analysis, and RDKit-assisted substructure annotation. Because the KNIME RDKit extension version is not necessarily identical to the underlying RDKit library release, the underlying RDKit release is reported separately where available from the KNIME installation or execution logs.

Model interpretability analyses were implemented within the KNIME workflow using dedicated XAI/interpretability nodes. Permutation feature importance (PFI) was used for global descriptor-importance analysis, and SHapley Additive exPlanations (SHAP) were used for local and compound-level interpretation of model predictions; both were treated as interpretability approaches integrated into the KNIME workflow rather than as independently versioned external software packages.

SwissADME was accessed [47,48] on 15 July 2026 and used for physicochemical, drug-likeness, medicinal-chemistry, BOILED-Egg, gastrointestinal absorption, blood–brain barrier permeability, P-glycoprotein substrate, PAINS, Brenk alert, bioavailability, and synthetic-accessibility profiling. CYP inhibition outputs were not used because they were unavailable after the May 2026 SwissADME platform modification. Applicability-domain calculations were performed using Morgan fingerprints, descriptor-space nearest-neighbor distance, leverage analysis, and test-subset-calibrated composite AD scoring, as described in Section 4.11.

All primary supplementary datasets generated in this study are provided as Excel workbooks. Supplementary Table S1 contains the matched four-isoform ChEMBL matrix. Supplementary Table S2 reports the top isoform-selective ChEMBL inhibitors. Supplementary Table S3 provides the Random Forest-selected descriptor panels. Supplementary Table S4 contains PubChem experimental concordance and evidence-classification results. Supplementary Table S5 reports new and partially new PubChem candidate classifications. Supplementary Table S6A provides the QSAR/SAR-ranked top-ten novel candidates per isoform, and Supplementary Table S6B reports SwissADME-based final prioritization. Applicability-domain outputs, including test-subset AD stratification, candidate AD metrics, and Williams plots, are reported in Section 2.8 as Table 6 and Table 7, and Figure 8.

The complete workflow was designed to preserve strict separation between training, held-out testing, PubChem candidate prediction, experimental-evidence checking, SAR/ADME triage, and applicability-domain classification. PubChem experimental records were used only for concordance and novelty annotation after QSAR-based ranking. They were not used to train the models, calculate predicted selectivity indices, or reorder candidates.

## 5. Conclusions

This study presents an explainable, multi-isoform QSAR framework integrating ligand-based potency and selectivity prediction, PubChem similarity-based virtual screening, PubChem experimental-concordance assessment, XAI-supported interpretation, SAR/ADME-guided developability triage, and applicability-domain calibration into a single traceable pipeline for prioritizing isoform-selective human carbonic anhydrase (CA) I, CA II, CA IX, and CA XII inhibitors. Four isoform-specific stacked-ensemble models showed consistent held-out test-set performance, with $R^{2}$ values ranging from 0.607 to 0.727. Same-target PubChem concordance further supported model transferability, with Pearson correlations > 0.86, linear-fit $R^{2}$ values > 0.74, and MAE values < 0.35 $pK_{i}$ units across the four isoforms.

Descriptor-level interpretation, permutation feature importance, and SHAP-based analysis indicated that the models captured chemically plausible isoform-dependent structure–activity patterns rather than relying solely on opaque statistical correlations. CA II and CA IX prioritization was concentrated mainly in classical sulfonamide zinc-binding chemical space, whereas the CA I candidates were enriched in structurally distinct, non-classical scaffolds. Because all final CA I candidates were classified as low/out-of-domain, however, they should be interpreted as exploratory design hypotheses rather than high-confidence in-domain predictions.

SAR review and SwissADME profiling identified structural and developability liabilities that predicted potency and selectivity alone could not detect, including reactive sulfonyl chloride motifs and candidates with poor predicted absorption, multiple rule violations, and low bioavailability. Applicability-domain analysis provided an additional test-calibrated reliability layer, distinguishing model-supported candidates from extrapolative hypotheses. Integration of predicted activity, PubChem concordance, SAR/ADME developability, and AD support yielded twenty final candidates, five per isoform. The strongest overall support was observed for the CA II and CA XII panels, intermediate support for CA IX, and lower-confidence novelty-oriented support for CA I.

CID 4460361 illustrates the value of this multi-layer prioritization strategy. Although this CA XII candidate showed favorable predicted potency, selectivity, ADME profile, and applicability-domain support, its non-canonical sulfonate ester/sulfonate-like motif requires explicit mechanistic confirmation. This case emphasizes that ligand-based QSAR, ADME profiling, and applicability-domain membership can refine candidate confidence but cannot independently confirm Zn^2+^ coordination or binding mode.

As scoped, this study does not include zinc-aware molecular docking, molecular dynamics simulation, or experimental inhibition testing. These remain necessary next steps for structural and biochemical confirmation of the prioritized candidates. Overall, the workflow, datasets, and candidate-prioritization framework developed here provide a reusable and transparent foundation for isoform-selective CA inhibitor discovery and a broader template for combining predictive modeling with explicit reliability and developability assessment in ligand-based drug discovery.

## Figures and Tables

**Table 1:** Structural and ligand-based determinants of isoform-selective carbonic anhydrase inhibition. Representative most-selective inhibitors from the curated matched four-isoform ChEMBL dataset are shown with representative selectivity-associated rim/entrance residues, inferred rim environment, matched *K_i_* profile, selectivity index (SI) and its limiting off-target, and the dominant design strategy.

Isoform	Structural Exemplar	Representative Selectivity-Associated Rim/Entrance Residues	Rim Environment	Dominant Selective Chemotype/Pattern	Representative Example from Matched Matrix	Main Design Implication
CA I	1AZM	Val-68; Leu-131; Pro-201	Narrow, rigid, sterically constrained entrance	Compact non-classical scaffolds, especially carboxylate/coumarin-like chemotypes	CHEMBL2070207; indole–pyrazole carboxylic acid; CA I SI = 185.2	CA I selectivity can arise from compact entrance/rim complementarity rather than classical sulfonamide Zn^2+^ coordination
CA II	3HS4	Val-68; Phe-131; Pro-201	Narrow pocket with aromatic rim wall	Classical primary sulfonamides with compact aromatic, heteroaromatic, oxime-amide, or amide tails	CHEMBL4449193; benzenesulfonamide oxime-amide; CA II SI = 1186.0	CA II potency is strongly driven by Zn^2+^ anchoring plus favorable aromatic-tail accommodation; CA II remains the major sulfonamide off-target liability
CA IX	3IAI	Leu-68; Val-131; Glu-195	Wide, permissive, hydrophobic, topologically extended entrance	Non-classical coumarin/entrance-directed scaffolds and hydrophobic tail-tuned sulfonamides	CHEMBL577123; coumarin-carboxylic acid; CA IX SI = 141.7	CA IX selectivity can be achieved by exploiting its wider rim and reduced steric restriction while minimizing CA I/II inhibition
CA XII	1JCZ	Leu-70; Ala-131; Glu-190	Open, solvent-exposed, mixed polar–hydrophobic rim	Classical sulfonamide/sulfamate scaffolds with flexible heteroaryl or polar–hydrophobic tails	CHEMBL3342494; bis-sulfonamide cyanopyrrole; CA XII SI = 866.2	CA XII selectivity is favored by tail/rim compatibility within an open, Ala-131-permissive entrance region

Note. SI was calculated as: $SI_{target}=\frac{min(K_{i}\;of\;of\mathrm{f-t}arget\;isoforms)}{K_{i}\;of\;target\;isoform}$. Higher SI indicates stronger target preference within the matched CA I/CA II/CA IX/CA XII activity matrix. The full top-20 selective compounds for each isoform, including SMILES, *K_i_* profiles, limiting off-targets, and chemotype annotations, are provided in Supplementary Table S2. Structural interpretations are consistent with the rim/tail approach and non-classical CA inhibition literature, including Bonardi et al. [5], Mancuso et al. [13], Supuran [4,18], Kumar et al. [11], and Moi et al. [12].

**Table 2:** Top-ten molecular descriptors selected by Random Forest feature importance for each carbonic anhydrase isoform.

CA I		CA II		CA IX		CA XII	
Descriptor	RF Score	Descriptor	RF Score	Descriptor	RF Score	Descriptor	RF Score
SpMax2_Bh(e)	1	CATS2D_02_AP	1	CATS2D_02_AP	1	SpMax_AEA(dm)	1
SpMax2_Bh(v)	0.55	N-069	0.64	SHED_PL	0.58	minddssS	0.55
SpMax2_Bh(m)	0.48	SHED_PL	0.44	B04[C-S]	0.4	P_VSA_s_1	0.38
C-028	0.34	minsNH2	0.32	F04[C-S]	0.35	SM10_EA(bo)	0.22
SpMax3_Bh(m)	0.33	P_VSA_ppp_P	0.16	N-069	0.16	S-110	0.21
SHED_PL	0.28	SpMax3_Bh(m)	0.15	GATS4i	0.15	SpMax_B(s)	0.11
SpMin8_Bh(i)	0.28	F03[O-S]	0.13	minsNH2	0.15	MAXDN	0.1
nPyridines	0.26	SHED_AP	0.12	SpMin2_Bh(m)	0.14	mindO	0.09
nArCOOH	0.23	SpMax5_Bh(m)	0.12	SpMax3_Bh(m)	0.12	GATS5p	0.08
F03[O-S]	0.22	SpMax4_Bh(m)	0.1	minddssS	0.11	SpMax3_Bh(s)	0.08

Note. RF scores are normalized within each isoform-specific dataset, with the top-ranked descriptor assigned a score of 1.00. Descriptor abbreviations follow alvaDesc nomenclature. The selected descriptor profiles were used for model development and as an initial descriptor-level interpretation of isoform-specific ligand recognition. Full top-100 descriptor sets for each isoform are provided in Supplementary Table S3.

**Table 3:** Training- and held-out test-set performance of machine-learning models for $pK_{i}$ prediction across CA I, CA II, CA IX, and CA XII.

Dataset	Metric	RF Train	GLM Train	GB Train	ANN Train	Stacked Train	RF Test	GLM Test	GB Test	ANN Test	Stacked Test
CA I	MSE	0.177	1.41	0.114	0.352	0.097	0.711	1.398	0.674	0.625	0.503
	R^2^	0.912	0.296	0.943	0.824	0.952	0.615	0.242	0.635	0.661	0.727
	RMSE	0.42	1.187	0.337	0.593	0.311	0.843	1.182	0.821	0.791	0.709
CA II	MSE	0.179	1.121	0.353	0.384	0.071	0.664	1.317	0.759	0.742	0.599
	R^2^	0.908	0.421	0.817	0.802	0.963	0.688	0.382	0.644	0.652	0.719
	RMSE	0.423	1.059	0.594	0.62	0.267	0.815	1.148	0.871	0.861	0.774
CA IX	MSE	0.085	0.801	0.279	0.338	0.042	0.47	0.867	0.542	0.562	0.443
	R^2^	0.934	0.379	0.784	0.738	0.967	0.63	0.319	0.574	0.558	0.652
	RMSE	0.292	0.895	0.528	0.582	0.206	0.686	0.931	0.736	0.749	0.666
CA XII	MSE	0.089	0.716	0.12	0.3	0.039	0.45	0.743	0.468	0.605	0.427
	R^2^	0.916	0.32	0.886	0.715	0.963	0.586	0.317	0.57	0.444	0.607
	RMSE	0.298	0.846	0.347	0.548	0.198	0.671	0.862	0.684	0.778	0.654

Note. RF, Random Forest; GLM, Generalized Linear Model; GB, Gradient Boosting; ANN, Artificial Neural Network; Stacked, stacked ensemble. MSE, mean squared error; $R^{2}$, coefficient of determination; RMSE, root mean squared error. For Gaussian regression in H2O, residual deviance is numerically equivalent to MSE. Bold values indicate the best held-out test-set performance for each isoform and metric.

**Table 4:** External PubChem *K_i_* concordance of the four CA isoform-specific QSAR models.

Isoform	*N*	Pearson R	R^2^_Linear Fit	RMSE (*PK_I_*)	MAE (*PK_I_*)
**CA I**	767	0.895	0.802	0.560	0.339
**CA II**	835	0.889	0.790	0.628	0.348
**CA IX**	599	0.904	0.816	0.534	0.325
**CA XII**	446	0.864	0.746	0.564	0.332

**Table 5:** (**A**) Final top-5 prioritized new/partially novel candidates per CA isoform following SAR and SwissADME triage (CA I). (**B**) Final top-5 prioritized new/partially novel candidates per CA isoform following SAR and SwissADME triage (CA II). (**C**) Final top-5 prioritized new/partially novel candidates per CA isoform following SAR and SwissADME triage (CA IX). (**D**) Final top-5 prioritized new/partially novel candidates per CA isoform following SAR and SwissADME triage (CA XII).

(**A**)							
**Isoform**	**PubChem CID/Name/SMILES**	**Predicted *K_i_* nM**	**Predicted *pK_i_***	**Predicted SI**	**Δ*pK_i_***	**Chemotype**	**ADME Score**
CA I	142163499, methylpropyl 6-methyl-4-[4-(2-methylpropyl)phenyl]-2-sulfanylidene-3,4-dihydro-1H-pyrimidine-5-carboxylate, 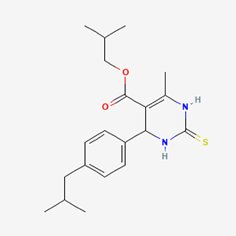	8.84	8.05	16.2	1.21	Thiourea/thioamide non-classical	92
CA I	83941633, 2-[2-(2-Ethyl-4-methoxyphenyl)ethyl]cyclopropane-1-carboxylic acid, 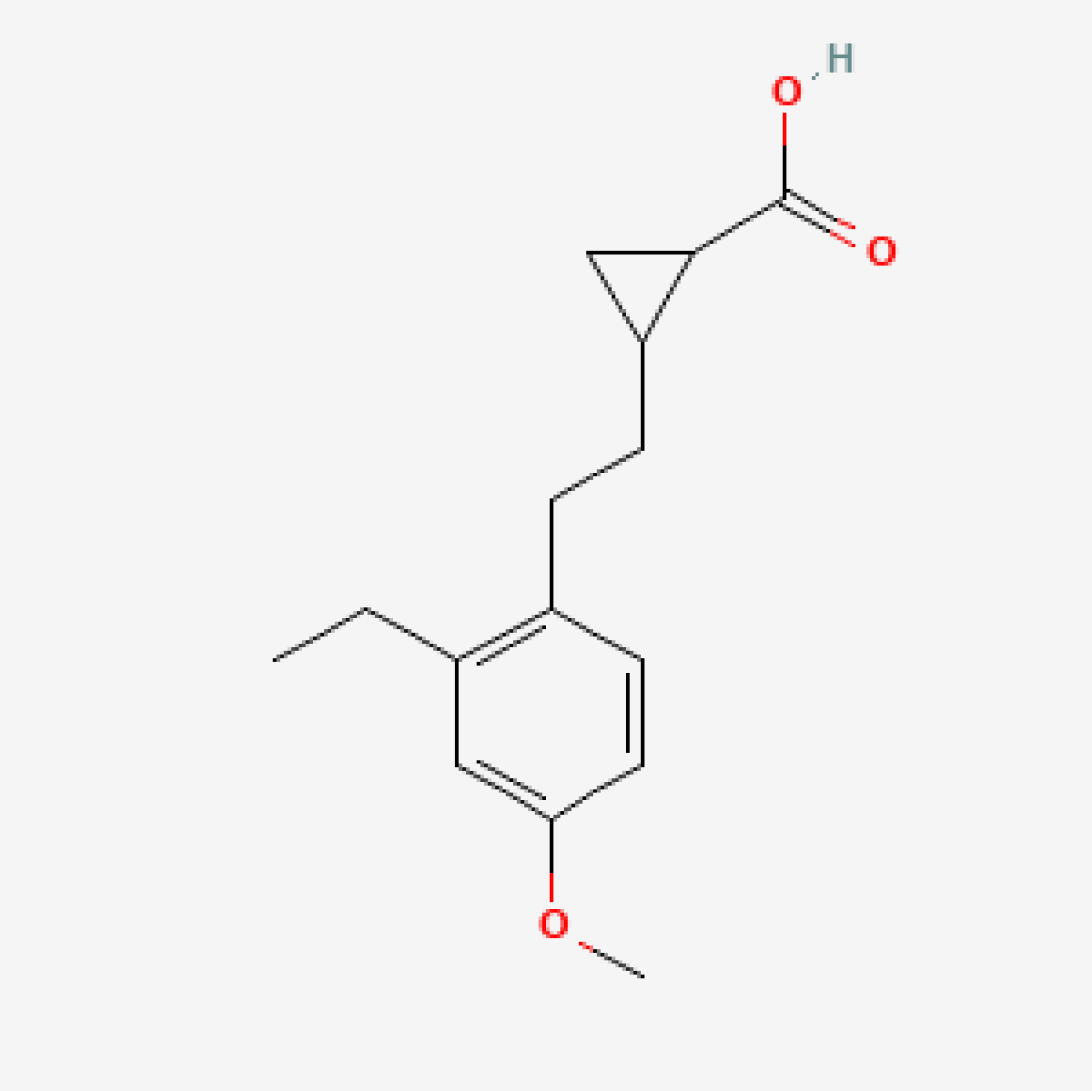	11.98	7.92	11.3	1.05	Non-classical carboxylate/acid	92
CA I	118612085, N-[4-[[2-[1-(4-chlorobenzoyl)-5-methoxy-2-methylindol-3-yl]acetyl]amino]butyl]cyclopenta-1,3-diene-1-carboxamide 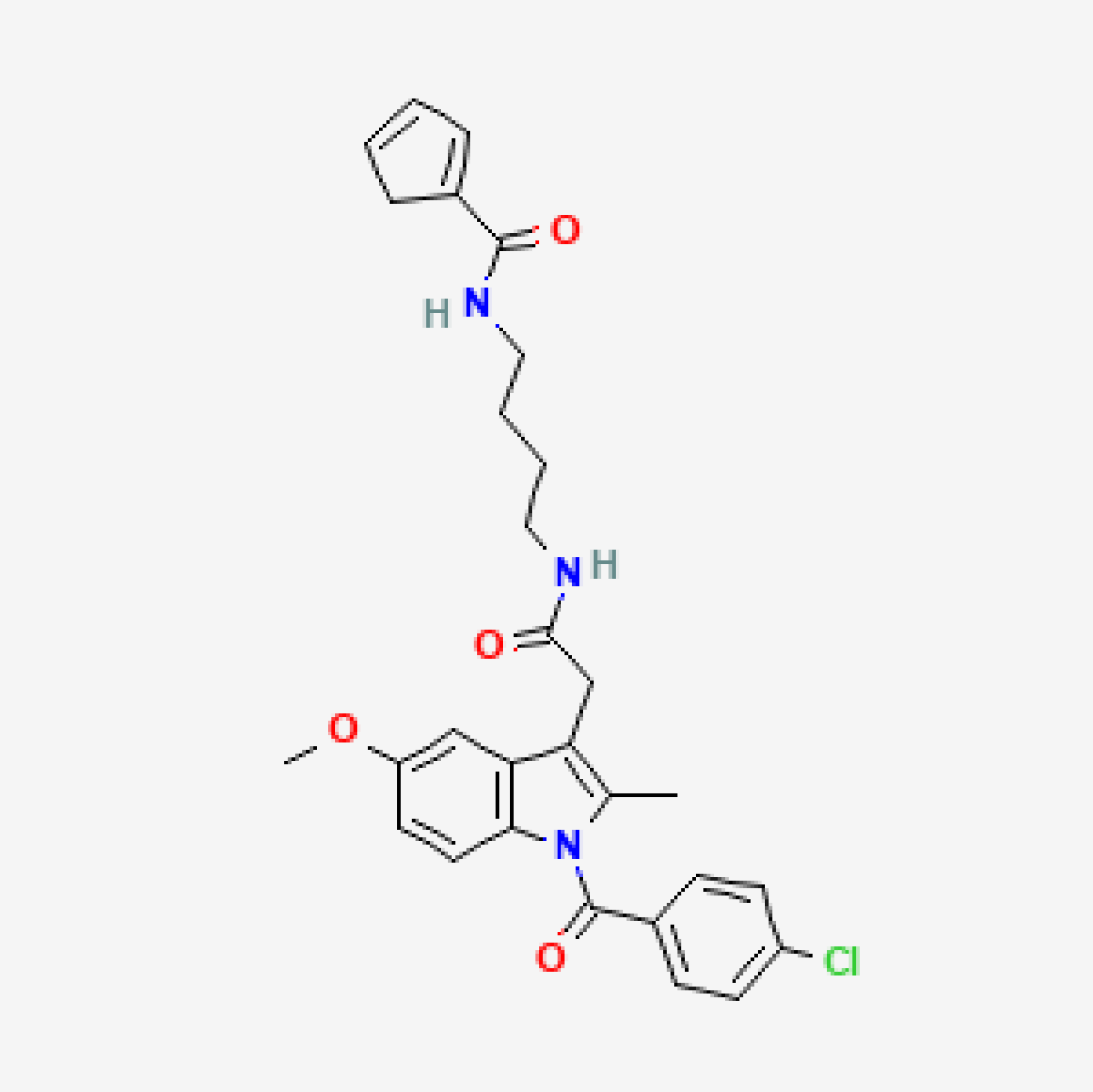	11.31	7.95	15.2	1.18	Heteroaryl/aromatic non-classical	64
CA I	8871903, 2-[4-[(4R)-5-acetyl-6-phenyl-2-sulfanylidene-3,4-dihydro-1H-pyrimidin-4-yl]phenoxy]acetonitrile, 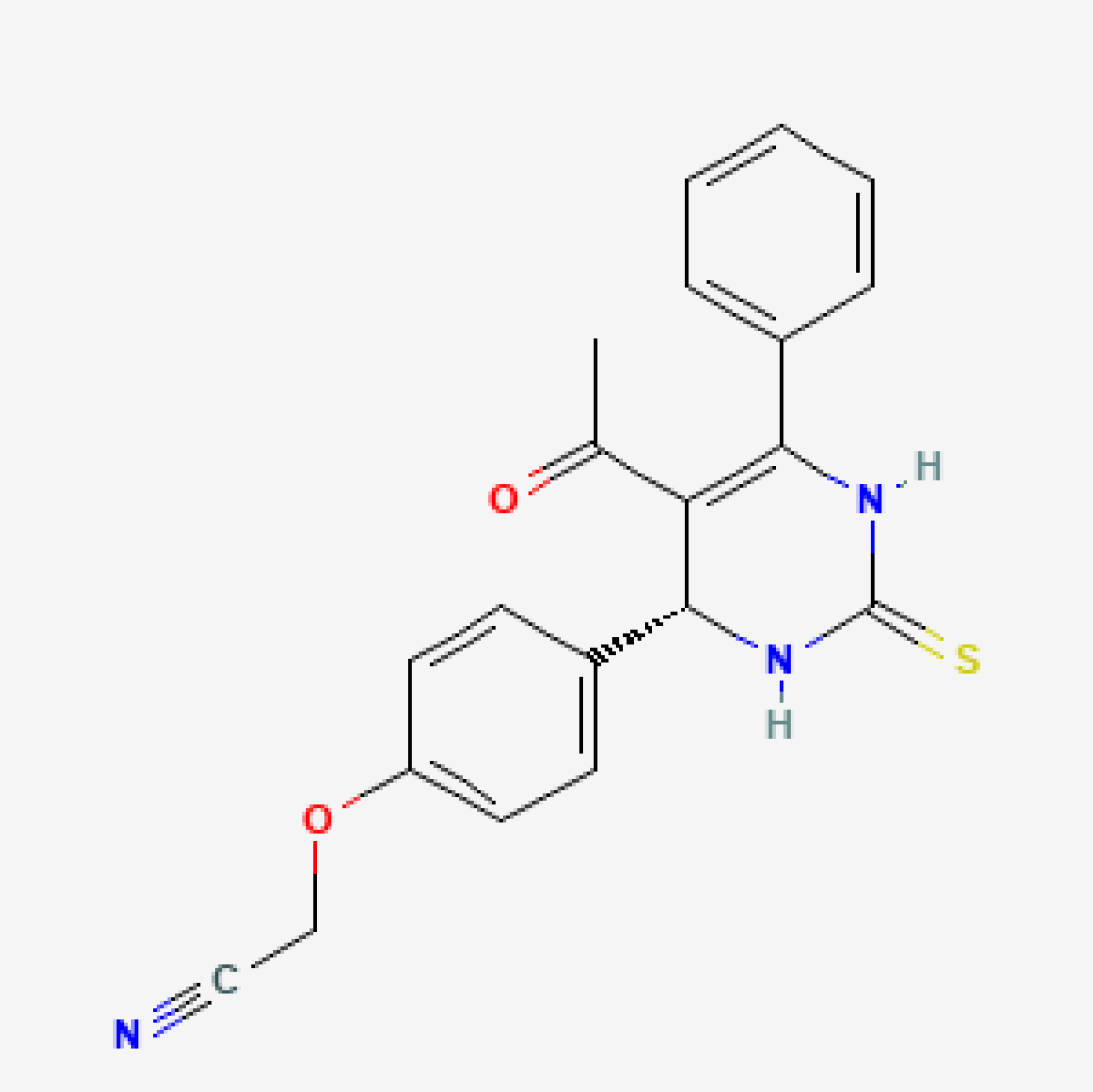	11.64	7.93	8.8	0.95	Thiourea/thioamide non-classical	87
CA I	78146320, 4-bromo-N-[[(4-bromophenyl)-phenylmethylidene]amino]aniline, 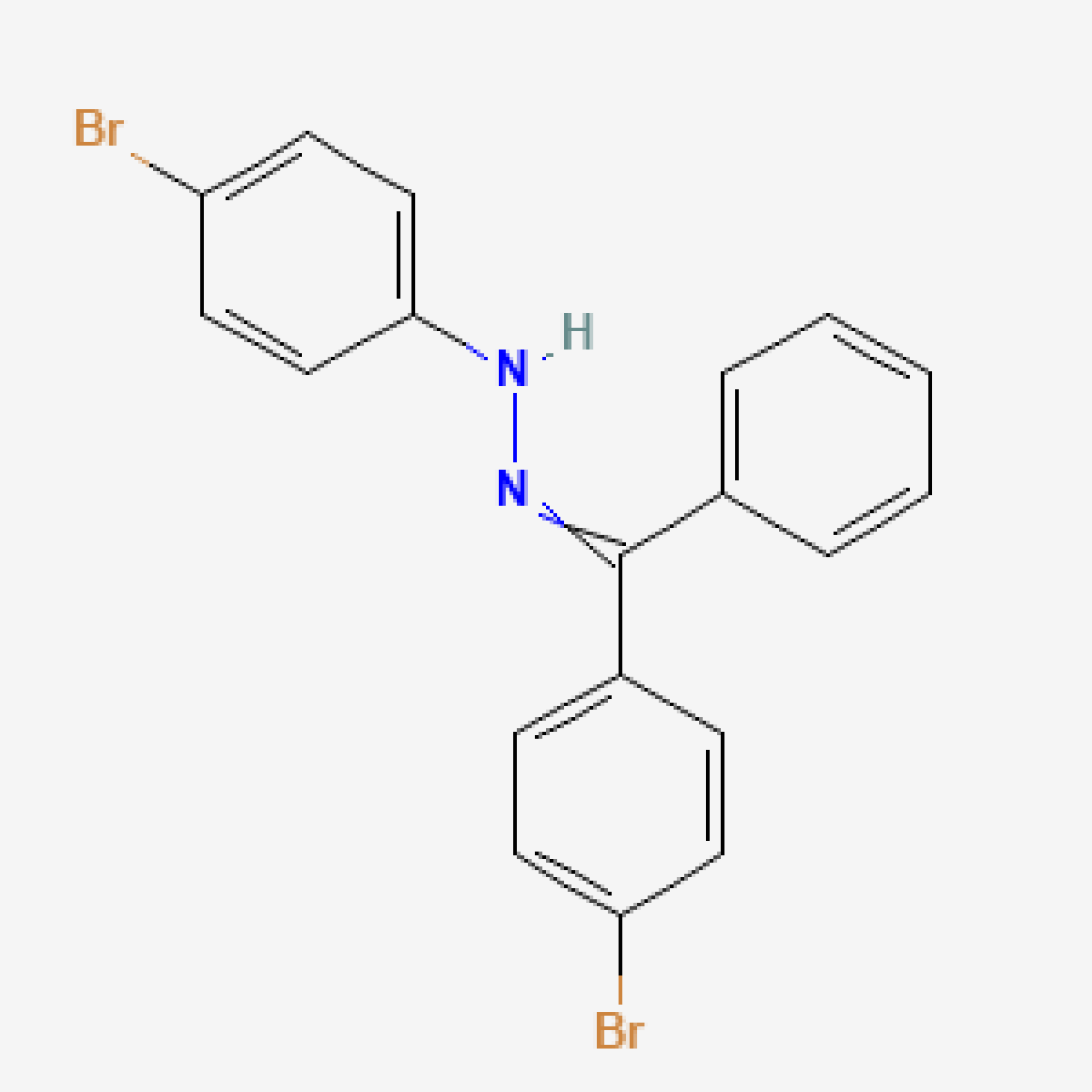	15.1	7.82	22.3	1.35	Hydrazone/polyaryl non-classical	52
(**B**)							
**Isoform**	**PubChem CID/Name/SMILES**	**Predicted *K_i_* nM**	**Predicted *pK_i_***	**Predicted SI**	**Δ*pK_i_***	**Chemotype**	**ADME Score**
CA II	110412583, tert-butyl N-[3-methyl-1-oxo-1-[2-(4-sulfamoylphenyl)ethylamino]butan-2-yl]carbamate, 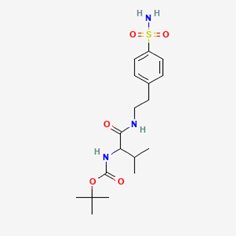	0.22	9.65	162.2	2.21	Classical primary sulfonamide	76
CA II	155812825, 4-[3-(4-Chlorophenyl)-5-(3,4,5-trimethoxyphenyl)-3,4-dihydropyrazol-2-yl]benzenesulfonamide, 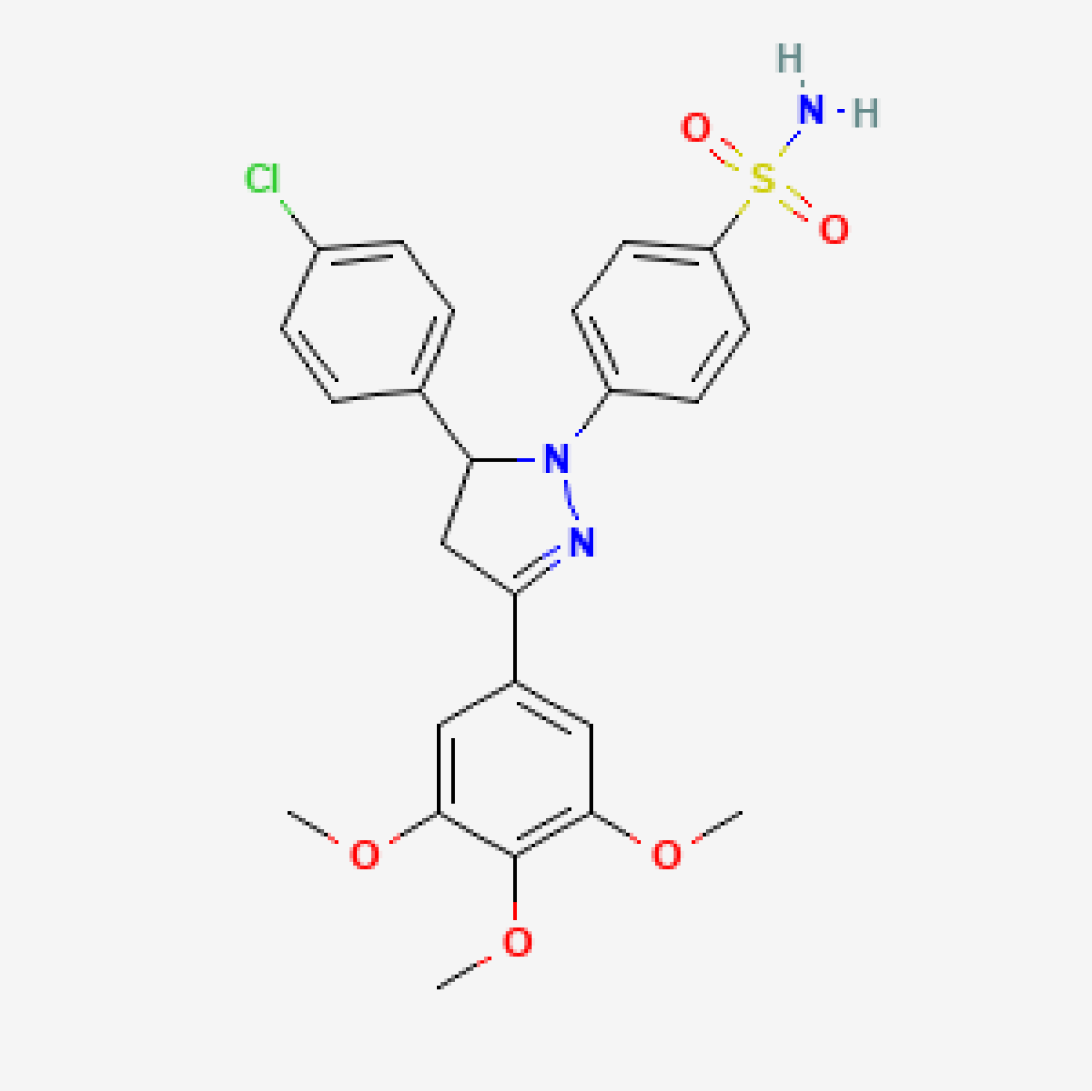	0.26	9.58	93.7	1.97	Classical primary sulfonamide	90
CA II	9821692, 4-(ethylamino)-7,7-dioxo-6-propyl-5,6-dihydro-4H-thieno [2,3-b]thiopyran-2-sulfonamide, 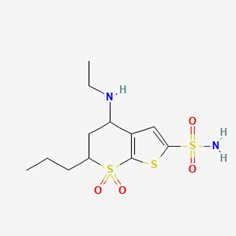	0.2	9.69	94.2	1.97	Classical primary sulfonamide	69
CA II	130448357, 4-[(1R,2R)-2-(5-phenyl-1,3-oxazol-2-yl)cyclopropyl]benzenesulfonamide, 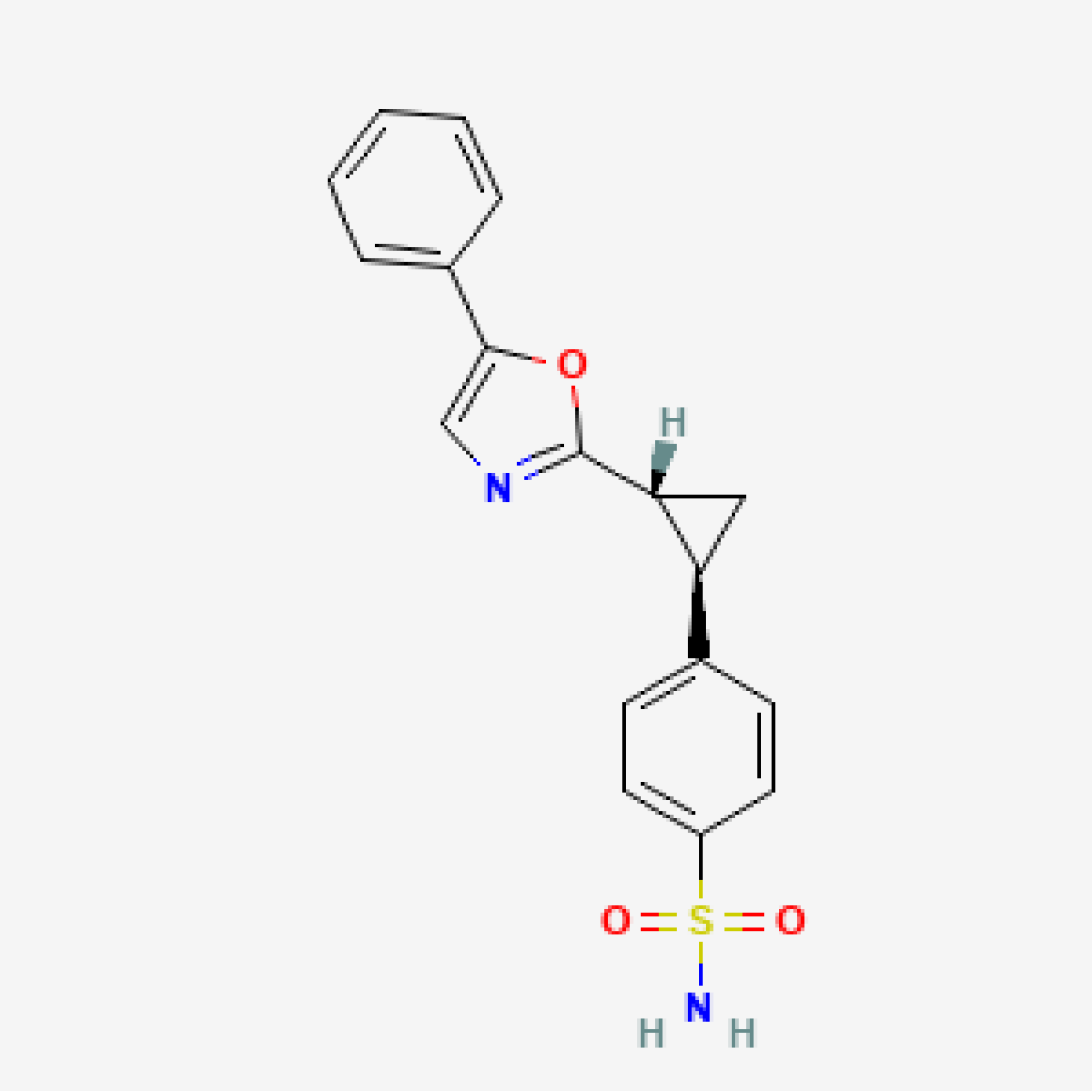	0.28	9.55	89	1.95	Classical primary sulfonamide	95
CA II	21735834, 1,1-Dioxo-2-propyl-3-(propylamino)-2,3-dihydrothieno [2,3-b]thiophene-5-sulfonamide, 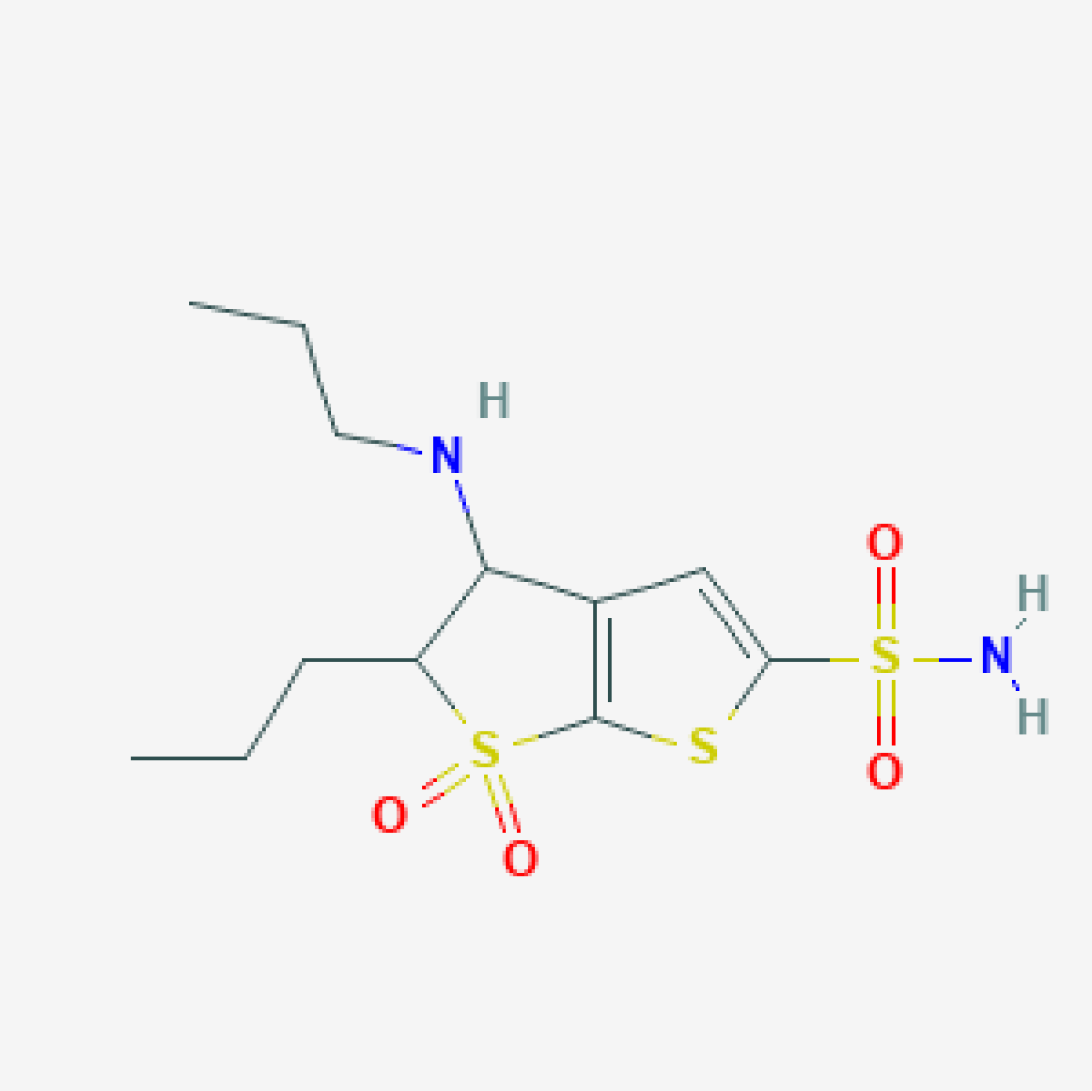	0.27	9.57	94.7	1.98	Classical primary sulfonamide	69
(**C**)							
**Isoform**	**PubChem CID/Name/SMILES**	**Predicted *K_i_* nM**	**Predicted *pK_i_***	**Predicted SI**	**Δ*pK_i_***	**Chemotype**	**ADME Score**
CA IX	169223349, N-methyl-N-(4-methyl-5-sulfamoyl-1,3-thiazol-2-yl)-2-[4-(2,3,4,5,6-pentadeuteriophenyl)phenyl]acetamide, 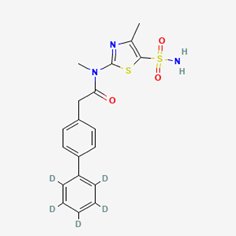	1.34	8.87	7.8	0.89	Classical primary sulfonamide	92
CA IX	418959, 4-[[(6-Amino-3-methylpyrido [2,3-b]pyrazin-8-yl)amino]methyl]benzenesulfonamide, 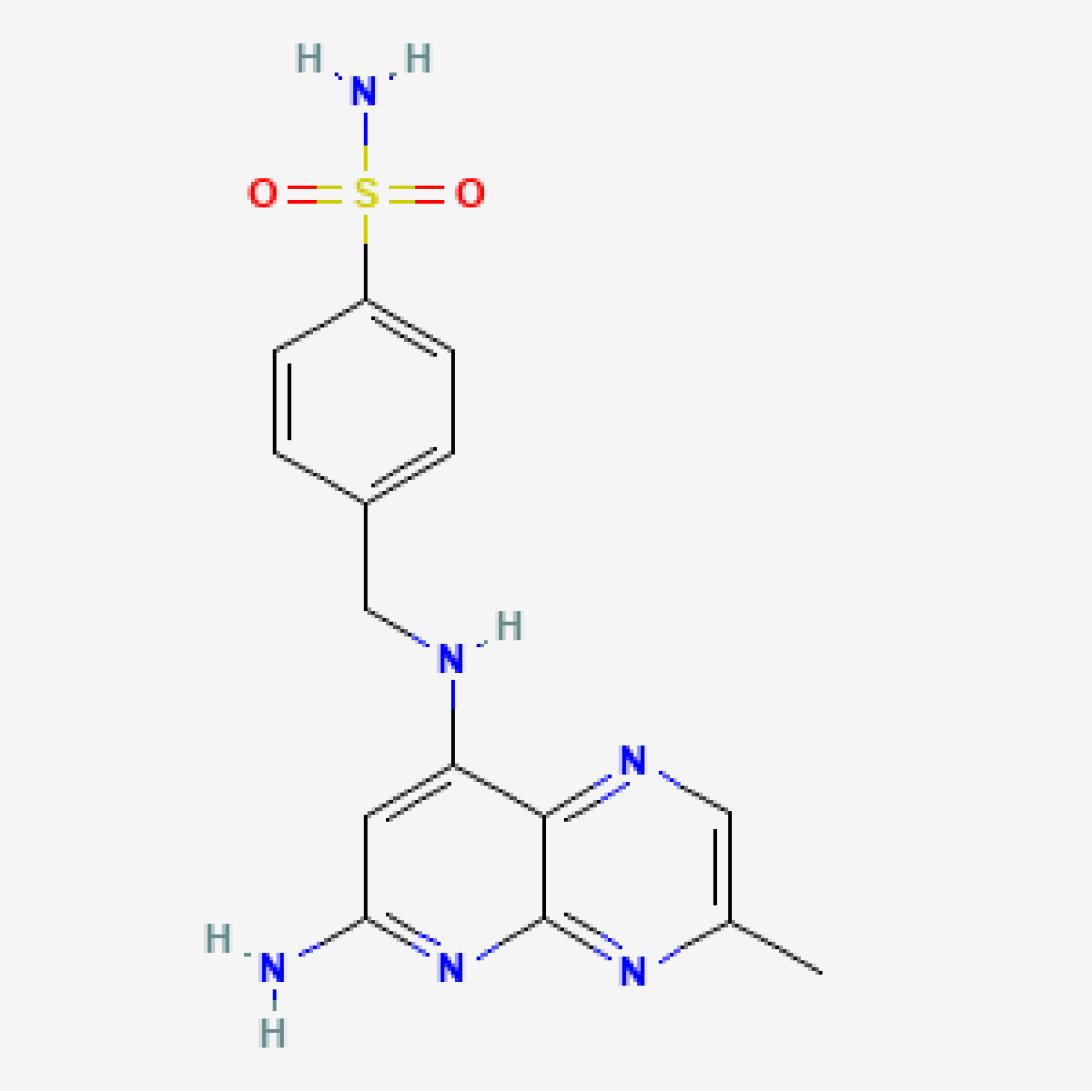	4.76	8.32	6.9	0.84	Classical primary sulfonamide	68
CA IX	419060, 4-[2-[(6-Aminopyrido [2,3-b]pyrazin-8-yl)amino]ethyl]benzenesulfonamide 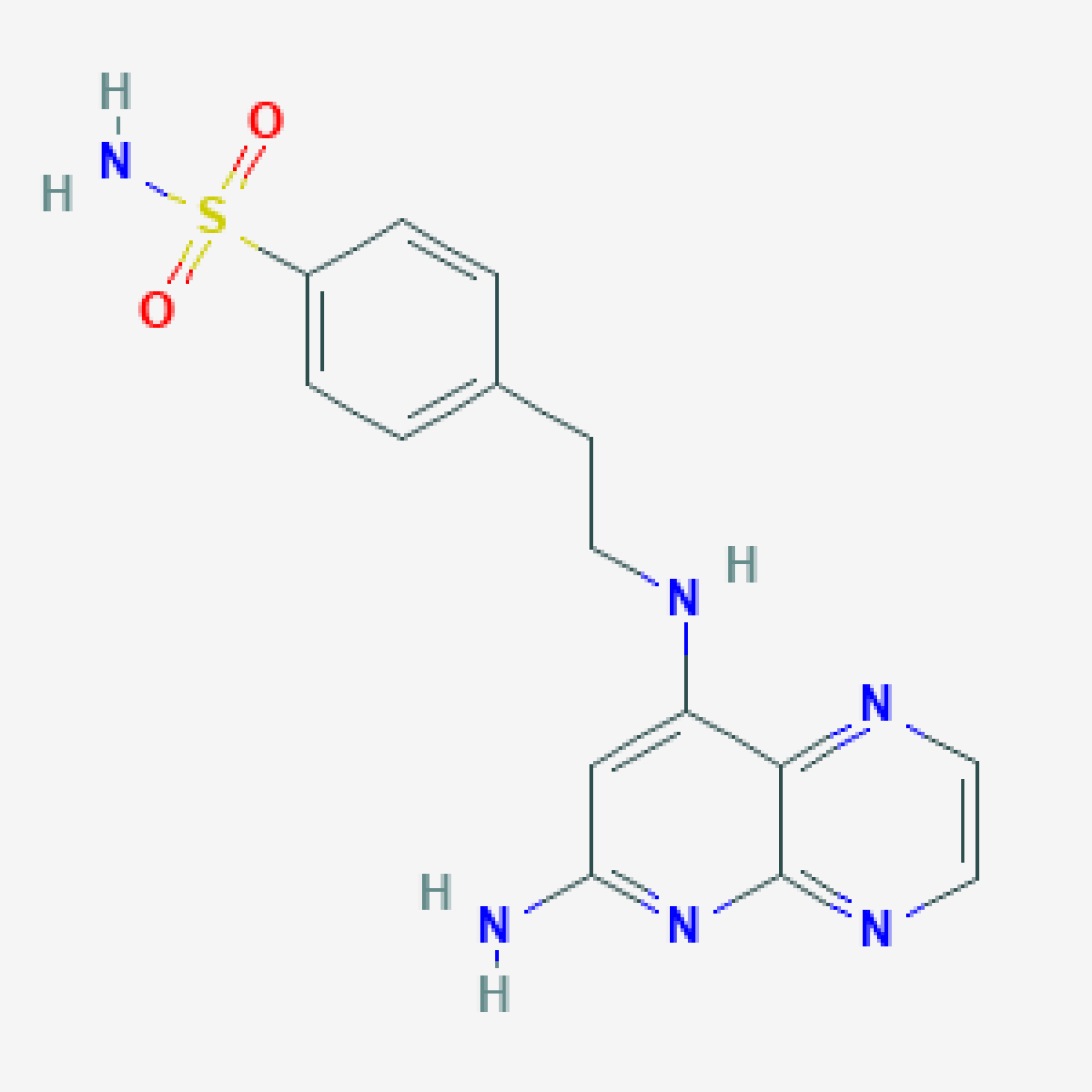	5.34	8.27	8	0.9	Classical primary sulfonamide	68
CA IX	114317887, 4-[(2-Chloro-6-hydroxyphenyl)methylamino]benzenesulfonamide, 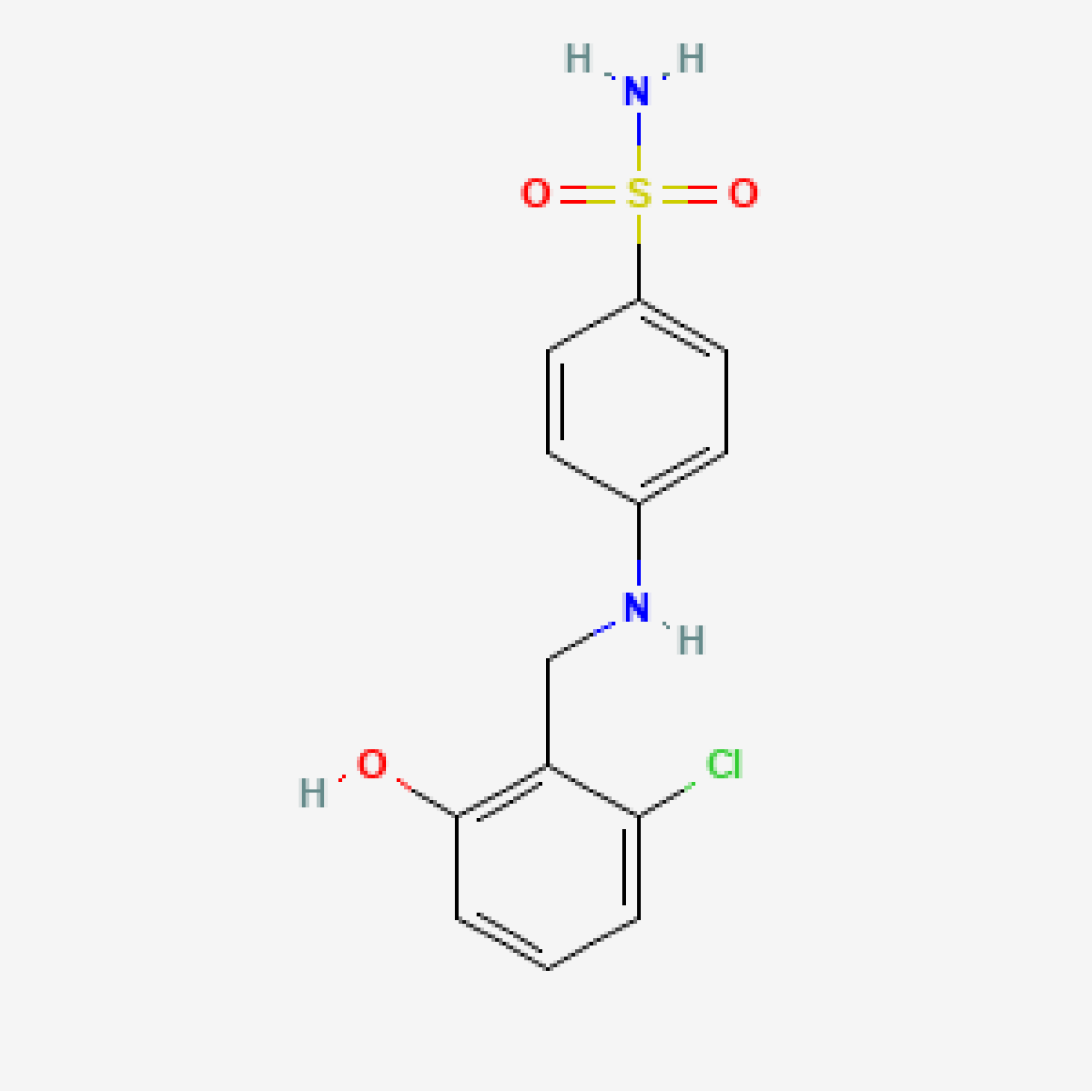	3.45	8.46	5.4	0.73	Classical primary sulfonamide	85
CA IX	21051322, N-(4-methyl-5-sulfamoyl-1,3-thiazol-2-yl)-2-(4-phenylphenyl)-N-propylacetamide, 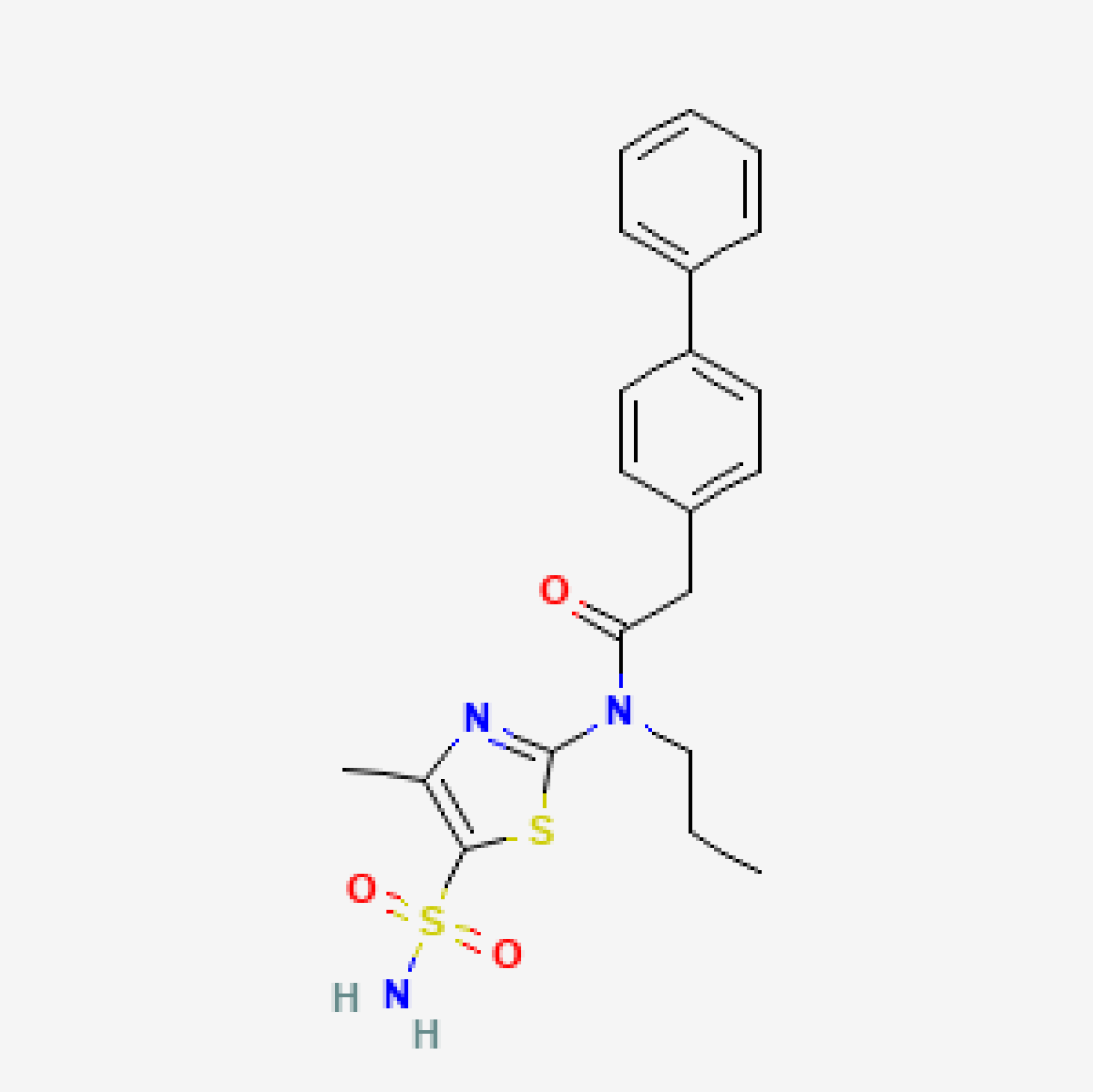	5.36	8.27	7.1	0.85	Classical primary sulfonamide	92
(**D**)							
**Isoform**	**PubChem CID/Name/SMILES**	**Predicted *K_i_* nM**	**Predicted *pK_i_***	**Predicted SI**	**Δ*pK_i_***	**Chemotype**	**ADME Score**
CA XII	4289218, 4-(2,5-Dioxopyrrolidin-1-yl)benzoate, 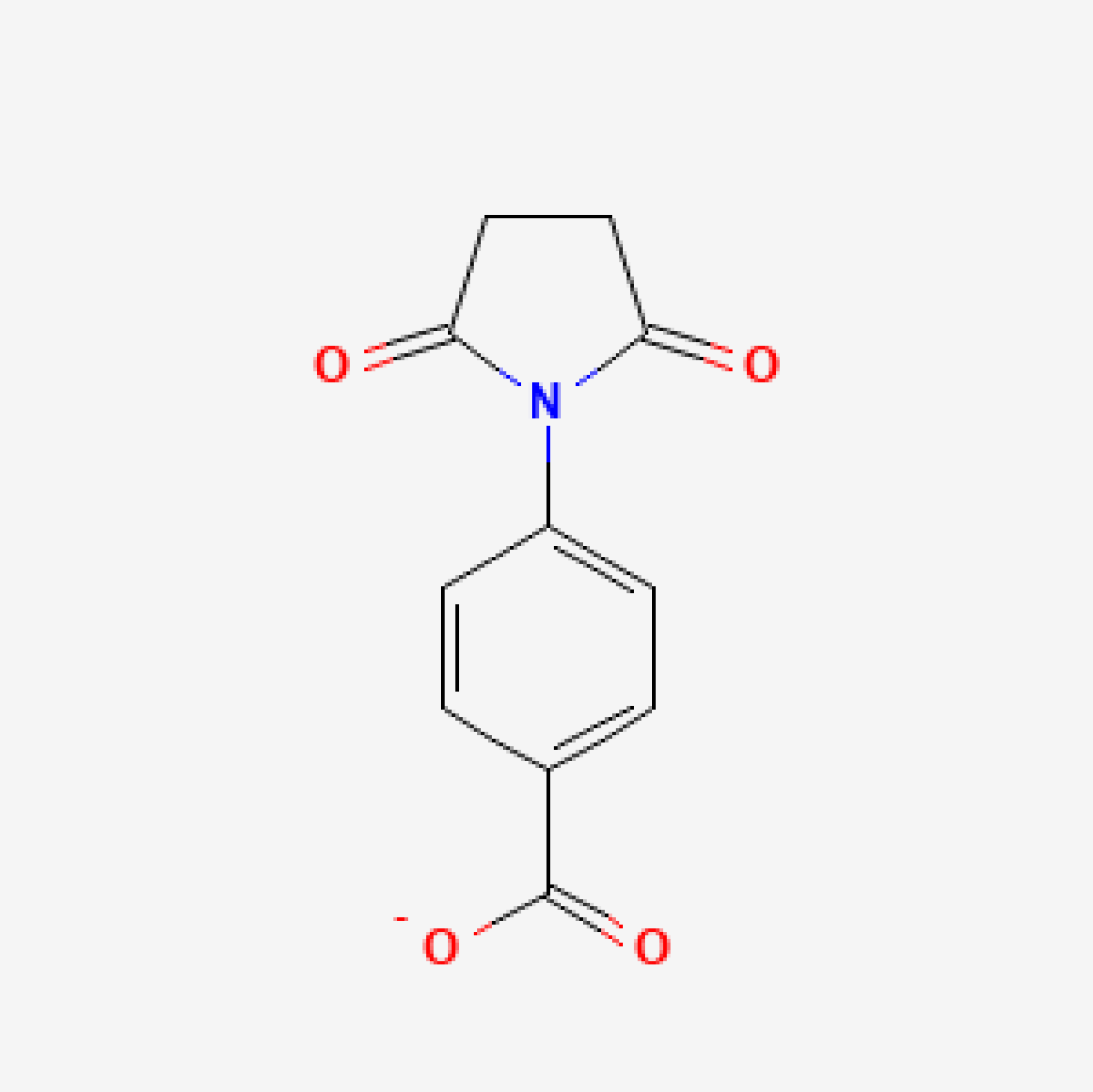	2.83	8.55	150.6	2.18	Non-classical carboxylate/acid	84
CA XII	155553321 ‡, [4-[[4-(4-Phenylpyrimidin-2-yl)piperazine-1-carbonyl]amino]phenyl] sulfamate, 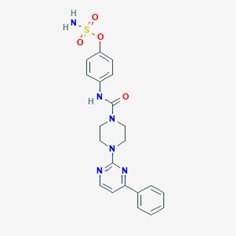	5.36	8.27	9260.5	3.97	Classical sulfamate	90
CA XII	155567815, [4-[(4-Heptylpiperazine-1-carbonyl)amino]phenyl] sulfamate, 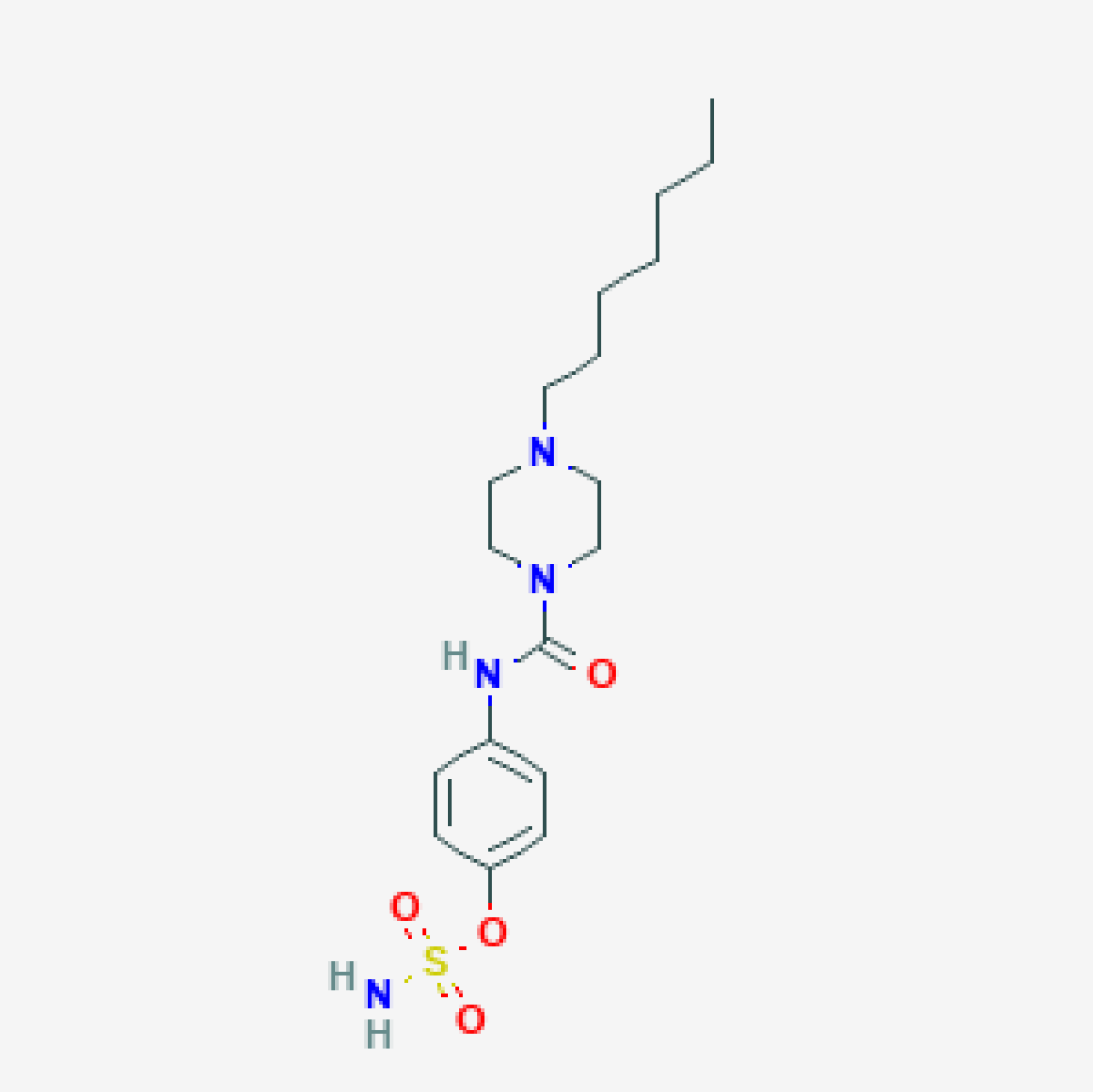	5.69	8.25	292.8	2.47	Classical sulfamate	81
CA XII	4460361 † 4-[[2-(4-Methoxyphenyl)acetyl]amino]benzenesulfonate, 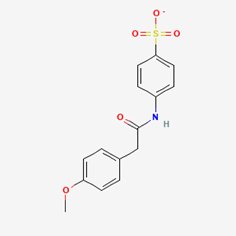	4.69	8.33	162.1	2.21	Sulfonate/sulfonate-like motif; mechanism caution	82
CA XII	155516037, [4-[[4-[4-[3-(Trifluoromethyl)phenyl]pyrimidin-2-yl]piperazine-1-carbonyl]amino]phenyl] sulfamate 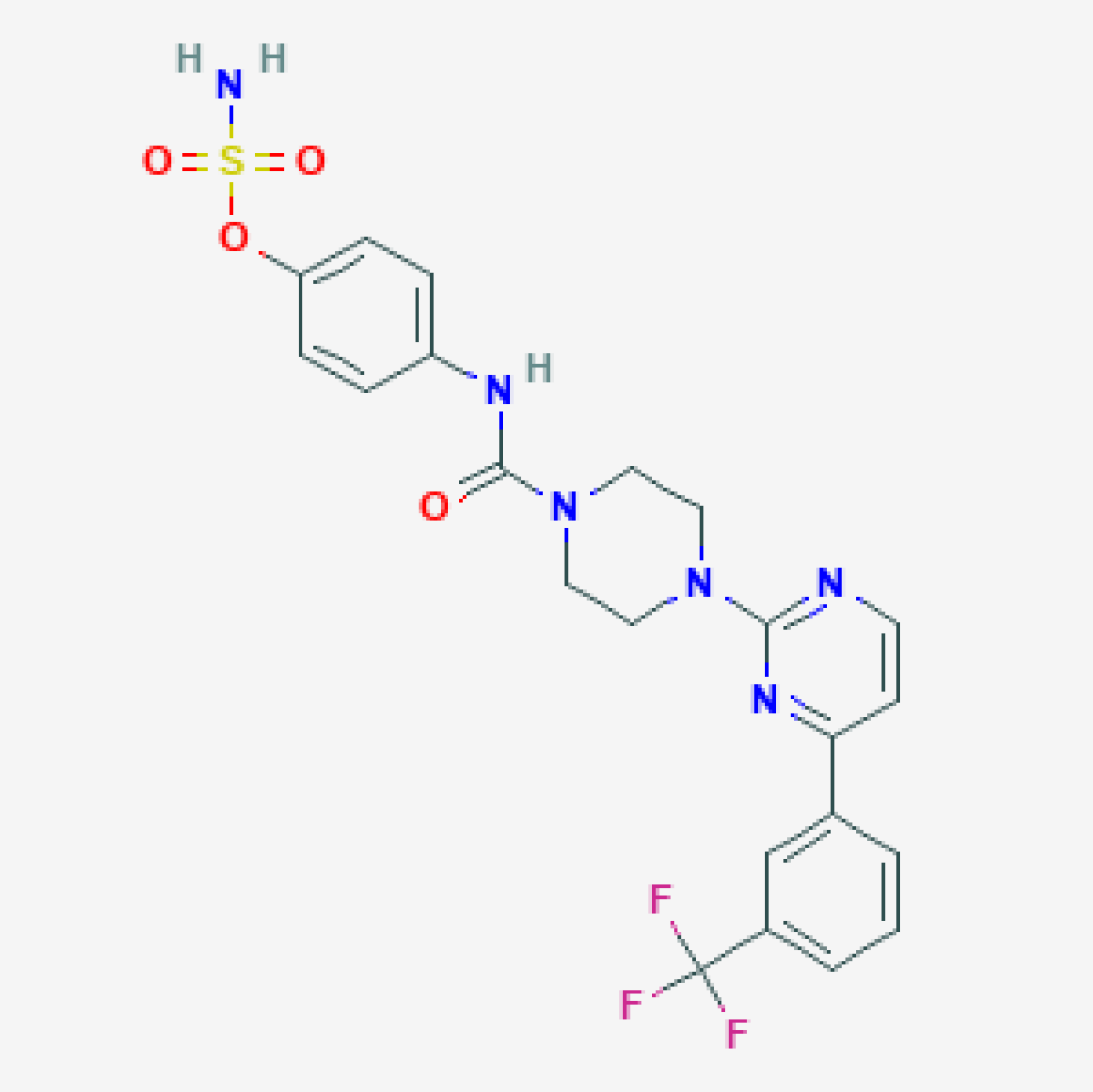	5.86	8.23	407.4	2.61	Classical sulfamate	72

† CID 4460361 is retained as a non-reactive CA XII candidate with acceptable ADME properties, but its sulfonate ester/sulfonate-like motif lacks the sulfonamide or sulfamate N–H generally required for canonical CA Zn^2+^ coordination. Its predicted binding mode should therefore be treated as provisional pending docking confirmation. ‡ CID 155553321 shows an unusually high predicted SI. This reflects an extreme model-derived separation between predicted CA XII potency and predicted off-target inactivity, and should be interpreted as a prioritization signal rather than confirmed quantitative selectivity.

**Table 6:** Test-subset prediction error stratified by applicability-domain (AD) class across the four carbonic anhydrase (CA) isoforms. n, number of held-out test compounds in the corresponding AD class; MAE and RMSE reported in *pK_i_* units.

Isoform	AD Class	n	MAE (*pK_i_*)	RMSE (*pK_i_*)
CA I	Very high	827	0.438	0.627
CA I	High	238	0.512	0.681
CA I	Moderate	188	0.568	0.779
CA I	Low/out-of-domain	188	0.691	0.968
CA II	Very high	879	0.465	0.685
CA II	High	260	0.497	0.692
CA II	Moderate	176	0.573	0.860
CA II	Low/out-of-domain	225	0.804	1.064
CA IX	Very high	645	0.440	0.587
CA IX	High	157	0.478	0.665
CA IX	Moderate	163	0.532	0.706
CA IX	Low/out-of-domain	162	0.682	0.883
CA XII	Very high	511	0.413	0.550
CA XII	High	145	0.489	0.615
CA XII	Moderate	100	0.563	0.759
CA XII	Low/out-of-domain	128	0.688	0.929

**Table 7:** Applicability-domain metrics for the twenty final prioritized candidates, five per isoform. Maximum Tanimoto and mean-5-nearest-neighbor similarity were computed using Morgan fingerprints with radius 2 and 2048 bits. $D_{5NN}$ is the mean standardized Euclidean distance to the five nearest training compounds in descriptor space. Leverage warning thresholds were $h^{*}=0.053$ for CA I, $h^{*}=0.049$ for CA II, $h^{*}=0.067$ for CA IX, and $h^{*}=0.086$ for CA XII. AD class was assigned using the composite-score thresholds defined above. A maximum Tanimoto value of 1.000 indicates identity under the selected fingerprint representation and should not automatically be interpreted as exact structural identity. Candidates are listed according to their final QSAR/SAR/ADME prioritization order, not by AD score.

Isoform	PubChem CID	Max. Tanimoto	Mean-5NN	D5NN	Leverage	AD Score	AD Class
CA I	142163499	0.554	0.520	4.805	0.015	0.403	Low/out-of-domain
CA I	83941633	0.440	0.417	7.814	0.035	0.066	Low/out-of-domain
CA I	118612085	0.675	0.483	10.652	0.026	0.339	Low/out-of-domain
CA I	8871903	0.492	0.459	6.478	0.018	0.235	Low/out-of-domain
CA I	78146320	0.458	0.399	8.173	0.028	0.092	Low/out-of-domain
CA II	110412583	1.000	0.764	2.582	0.009	0.963	Very high
CA II	155812825	0.855	0.750	1.469	0.010	0.958	Very high
CA II	9821692	1.000	0.840	1.981	0.017	0.929	Very high
CA II	130448357	0.596	0.491	8.564	0.057	0.097	Low/out-of-domain
CA II	21735834	0.564	0.526	2.882	0.019	0.511	Moderate
CA IX	169223349	1.000	0.813	2.323	0.016	0.952	Very high
CA IX	418959	0.471	0.456	6.302	0.027	0.175	Low/out-of-domain
CA IX	419060	0.520	0.496	6.458	0.027	0.159	Low/out-of-domain
CA IX	114317887	0.610	0.547	2.519	0.008	0.648	Moderate
CA IX	21051322	0.830	0.700	2.873	0.014	0.957	Very high
CA XII	4289218	0.690	0.441	7.097	0.030	0.545	Moderate
CA XII	155553321	0.790	0.656	4.925	0.016	0.896	Very high
CA XII	155567815	0.636	0.584	5.103	0.018	0.616	Moderate
CA XII	4460361	0.780	0.598	4.812	0.021	0.893	Very high
CA XII	155516037	0.818	0.607	5.136	0.022	0.867	Very high

Note: AD class was assigned using the unrounded AD score. CID 114317887 had an AD score of approximately 0.648 and therefore remained classified as moderate because it did not reach the high-AD threshold of 0.65.

## Data Availability

The original contributions presented in this study are included in the article and Supplementary Materials. Further inquiries can be directed to the corresponding author.

## Supplementary Materials

The following supporting information can be downloaded at: https://www.mdpi.com/article/10.3390/ph19081322/s1, Supplementary Table S1: matched four-isoform ChEMBL matrix; Supplementary Table S2: top isoform-selective ChEMBL inhibitors; Supplementary Table S3: Random Forest-selected descriptor panels; Supplementary Table S4: PubChem experimental concordance and evidence-classification results; Supplementary Table S5: new and partially new PubChem candidate classifications; Supplementary Table S6: (A) QSAR/SAR-ranked top-ten novel candidates per isoform; (B) SwissADME-based final prioritization.
